# A Phase 3, randomized, non-inferiority study of a heterologous booster dose of SARS CoV-2 recombinant spike protein vaccine in adults

**DOI:** 10.1038/s41598-023-43578-w

**Published:** 2023-10-03

**Authors:** Prasad S. Kulkarni, Bhagwat Gunale, Sunil Kohli, Sanjay Lalwani, Srikanth Tripathy, Sonali Kar, Sidram Raut, Praveen Kulkarni, Aditi Apte, Ashish Bavdekar, Hira Lal Bhalla, Joyce S. Plested, Shane Cloney-Clark, MingZhu Zhu, Raj Kalkeri, Melinda Pryor, Stephanie Hamilton, Madhuri Thakar, Ranga S. Sannidhi, Punjita Baranwal, Chetanraj Bhamare, Abhijeet Dharmadhikari, Manish Gupta, Cyrus S. Poonawalla, Umesh Shaligram, Dhananjay Kapse, COVOVAX-Booster Study Group, Vineet Jain, Nidhi Goyal, Alok Arya, Temsunaro Rongsen-Chandola, Sonali Palkar, Neeta Hanumante, Arjun Kakrani, Shahzad Beg Mirza, Savita Mahajan, Rakesh Kothavale, Pramod Chandra Samantaray, Aparna Kodre, M. R. Mythily, M. Shwethashree, Girish Dayma, Tejas Patel, Surekha Kishore

**Affiliations:** 1 Serum Institute of India Pvt Ltd, Manjari (Bk), Administration Building, Poonawalla Biotechnology Park SEZ, Taluka Haveli, Pune, India; 2 Hamdard Institute of Medical Sciences and Research, New Delhi, India; 3 Bharati Vidyapeeth Deemed University Medical College and Hospital, Pune, India; 4 Dr. D. Y. Patil Medical College, Hospital and Research Centre, Pune, India; 5 Kalinga Institute of Medical Sciences, Bhubaneswar, India; 6 Noble Hospital Pvt. Ltd, Pune, India; 7 JSS Academy of Higher Education and Research, Mysore, India; 8 KEM Hospital Research Centre-Community Health Research Unit, P.O. Manchar, Pune, India; 9 All India Institute of Medical Sciences (AIIMS), Gorakhpur, India; 10 Novavax, Inc., Gaithersburg, USA; 11 360Biolabs, Melbourne, Australia; 12 ICMR - National AIDS Research Institute, Pune, India; 13 PPD, Benguluru, India

**Keywords:** Infectious diseases, Viral infection

## Abstract

Due to waning immunity following primary immunization with COVID-19 vaccines, booster doses may be required. The present study assessed a heterologous booster of SII-NVX-CoV2373 (spike protein vaccine) in adults primed with viral vector and inactivated vaccines. In this Phase 3, observer-blind, randomized, active controlled study, a total of 372 adults primed with two doses of ChAdOx1 nCoV-19 (n = 186) or BBV152 (n = 186) at least six months ago, were randomized to receive a booster of SII-NVX-CoV2373 or control vaccine (homologous booster of ChAdOx1 nCoV-19 or BBV152). Anti-S IgG and neutralizing antibodies (nAbs) were assessed at days 1, 29, and 181. Non-inferiority (NI) of SII-NVX-CoV2373 to the control vaccine was assessed based on the ratio of geometric mean ELISA units (GMEU) of anti-S IgG and geometric mean titers (GMT) of nAbs (NI margin > 0.67) as well as seroresponse (≥ 2 fold-rise in titers) (NI margin −10%) at day 29. Safety was assessed throughout the study period. In both the ChAdOx1 nCoV-19 prime and BBV152 prime cohorts, 186 participants each received the study vaccines. In the ChAdOx1 nCoV-19 prime cohort, the GMEU ratio was 2.05 (95% CI 1.73, 2.43) and the GMT ratio was 1.89 (95% CI 1.55, 2.32) whereas the difference in the proportion of seroresponse was 49.32% (95% CI 36.49, 60.45) for anti-S IgG and 15% (95% CI 5.65, 25.05) for nAbs on day 29. In the BBV152 prime cohort, the GMEU ratio was 5.12 (95% CI 4.20, 6.24) and the GMT ratio was 4.80 (95% CI 3.76, 6.12) whereas the difference in the proportion of seroresponse was 74.08% (95% CI 63.24, 82.17) for anti-S IgG and 24.71% (95% CI 16.26, 34.62) for nAbs on day 29. The non-inferiority of SII-NVX-CoV2373 booster to the control vaccine for each prime cohort was met. SII-NVX-CoV2373 booster showed significantly higher immune responses than BBV152 homologous booster. On day 181, seroresponse rates were ≥ 70% in all the groups for both nAbs and anti-S IgG. Solicited adverse events reported were transient and mostly mild in severity in all the groups. No causally related SAE was reported. SII-NVX-CoV2373 as a heterologous booster induced non-inferior immune responses as compared to homologous boosters in adults primed with ChAdOx1 nCoV-19 and BBV152. SII-NVX-CoV2373 showed a numerically higher boosting effect than homologous boosters. The vaccine was also safe and well tolerated.

## Introduction

In December 2019, a cluster of a novel coronavirus, known as 2019-nCoV, cases were identified in Wuhan, China^1^. As of 7 December 2022, there have been more than 642 million reported cases and more than 6.62 million deaths worldwide^2^. By the same time, India reported a total of more than 44.67 million cases with more than 0.53 million fatalities^3^.

The immunity achieved through natural infection by SARS-CoV-2 has been shown to last for many months^4^. The durability of immune response is affected by the waning of antibody titers over time, and the emergence of novel variants of the SARS-CoV-2 virus which may need higher titers to neutralize^5^. This is likely to necessitate booster doses of COVID-19 vaccines, and potentially periodic boosters. Boosters will generate higher antibody levels against the original strain and may also generate immunity specifically against novel variants^6^.

A SARS-CoV-2 recombinant spike (rS) protein nanoparticle vaccine adjuvanted with Matrix-M™ (NVX-CoV2373) was developed in USA. NVX-CoV2373 was assessed in a Phase 1/2^7,8^, Phase 2a/b^9^ and two Phase 3 studies^10–12^ which showed that it was safe, immunogenic with high efficacy. The overall vaccine efficacy for PCR-confirmed symptomatic SARS-CoV-2 infection was around 90% and 79% in adults^11,12^ and adolescents^13^, respectively. After technology transfer, the vaccine is also manufactured in India (SII-NVX-CoV2373). SII-NVX-CoV2373 was tested for primary immunization in India when it was successfully immuno-bridged with NVX-CoV2373^14^.

It has become evident that the protection offered against COVID-19 wanes after a two-dose schedule of COVID-19 vaccines^15,16^. Therefore, the potential role of third or booster dose against COVID-19 was evaluated. A single booster dose of NVX-CoV2373 administered to adults approximately 6 months following NVX-CoV2373 primary series showed a high immune response for both the prototype strain and all variants evaluated^6^. The vaccine also showed excellent boosting after two doses of ChAdOx1 nCoV-19 or BNT162b2 vaccines^17^.

In India, the nation-wide primary COVID-19 immunization programme was started on 16 January 2021 with two vaccines: Covishield (ChAdOx1 nCoV-19, a viral vector vaccine) and Covaxin (BBV152, a whole virion inactivated vaccine)^18^ and these two vaccines were used for primary immunization of > 90% of the adult population^19^. On 10 January 2022, the country started homologous booster immunization with these vaccines^20^. SII-NVX-CoV2373 was not used for primary immunization in India when this study started and therefore, a homologous booster of SII-NVX-CoV2373 was not evaluated in the study. While some studies have shown that a heterologous booster induces higher immune responses than a homologous booster, there are some studies which show the opposite finding^21–23^. The present study assessed the heterologous booster response to SII-NVX-CoV2373 compared to homologous booster doses of ChAdOx1 nCoV-19 or BBV152 in Indian adults as a third dose in individuals who had previously received two doses of ChAdOx1 nCoV-19 or BBV152 at least 6 months ago. Homologous boosters of ChAdOx1 nCoV-19 or BBV152 were chosen as a control group for respective prime cohorts because homologous booster vaccinations were already implemented in India’s immunization programme.

## Materials and methods

This was a Phase 3, multicentric, non-inferiority, observer-blinded, randomized, active controlled study in adults who had completed primary immunization with either ChAdOx1 nCoV-19 or BBV152 at least 6 months ago. A total of 372 eligible participants were enrolled in two prime cohorts of 186 participants each with 1:1 allocation to SII-NVX-CoV2373 or control vaccine. The study was conducted from May 2022 to December 2022 at 8 hospitals in India. The study was registered on Clinical Trials Registry-India (CTRI/2022/04/042017).

The primary objective was to demonstrate non-inferiority of the SII-NVX-CoV2373 heterologous booster against the homologous booster of control vaccine using anti-S IgG and neutralizing antibodies (nAbs) for ChAdOx1 nCoV-19, and BBV152 Prime cohorts, separately. The secondary objective was to assess the tolerability and reactogenicity profile of SII-NVX-CoV2373 booster in comparison with the control vaccine. As an exploratory objective, the study also assessed immune responses by nAbs, anti-S IgG and human ACE2 (hACE2) receptor binding inhibition assays against variants of concern (VoC).

### Study participants

The study participants were healthy adults aged ≥ 18 years who gave written informed consent and who had completed primary COVID-19 immunization schedule of two doses with either ChAdOx1 nCoV-19 or BBV152 at least 6 months ago. Participants with a history of laboratory confirmed COVID-19, history of allergic reactions after previous vaccinations, hypersensitivity to any component of study vaccines, any condition with impaired/altered function of immune system, any clinically significant systemic disorder were excluded. Clinically well-controlled comorbidities were allowed.

### Study vaccines

SII-NVX-CoV2373 (SARS-CoV-2 recombinant spike protein nanoparticle vaccine with Matrix-M™ adjuvant, Serum Institute of India Pvt. Ltd. [SIIPL]), was available as a ready to use liquid formulation. Each single dose of 0.5 mL contained 5 μg antigen and 50 μg Matrix-M adjuvant (Batch Number: 4301MF017, Expiry: August 2022).

The comparator in the ChAdOx1 nCoV-19 prime cohort was Covishield™ [ChAdOx1 nCoV-19 Corona Virus Vaccine (Recombinant), SIIPL] which is a recombinant, replication-deficient chimpanzee adenovirus vector encoding the SARS-CoV-2 Spike glycoprotein. One dose (0.5 mL) contains 5 x 10^10^ virus particles (Batch Number: 4121MC169, Expiry: August 2022).

The comparator used in the BBV152 prime cohort was Covaxin® [Whole Virion Inactivated SARS-CoV-2 Vaccine, Bharat Biotech International Ltd]. Each dose of 0.5 mL contains 6 µg of Whole Virion, Inactivated (SARS-CoV-2) Antigen (strain NIV-2020–770) along with Aluminum Hydroxide Gel, TLR7/8 Agonist and 2-Phenoxyethanol (Batch Number: C10/21004, Expiry: November 2022).

All study vaccines (SII-NVX-CoV2373, ChAdOx1 nCoV-19 and BBV152) were administered as a single dose of 0.5 mL intramuscularly in the deltoid.

### Randomization and blinding

The randomization scheme was generated using SAS version 9.4 (SAS Institute Inc, USA) for Interactive Response Technology (IRT) with 1:1 allocation to SII-NVX-CoV2373 or control vaccine separately for each prime cohort. The eligible study participants were enrolled and randomized in the study online through IRT. Each study participant was assigned a randomization number to allocate vaccine group after entry of eligibility data into the IRT system. The study participants, the study personnel responsible for the evaluation of any study endpoints and the laboratories involved in the immunogenicity testing were blinded to the treatment allocation. Personnel involved in getting randomization code by accessing IRT and vaccine administration were unblinded, and they did not conduct any study evaluations. The unblinded site personnel prepared the vaccine out of view of the study participant, and administered the vaccine with the final prepared syringe.

### Study procedures

All participants received a single dose of 0.5 mL of either SII-NVX-CoV2373 or control vaccine on day 1 as per randomization. Blood samples were collected at baseline (day 1), days 29 (+7), 91 (+7) and 181 (+14) for immunogenicity assessments including anti-S IgG and nAbs. Additionally, a blood sample was collected in a subset for assessment of cell mediated immune (CMI) responses at baseline, days 29 and 181.

A nasopharyngeal/nasal and/or throat swab was collected for RT-PCR test for detection of SARS-CoV-2 infection at baseline before vaccination on day 1 and also, if participants presented with symptoms of suspected COVID-19 or had a history of contact with a confirmed COVID-19 case at any time during the study.

Physical examination and vital sign evaluations were performed and medical history and prior/concomitant medications were recorded on days 1, 29, 91 and 181. Vital signs were also measured at 30 min post-vaccination.

### Immunogenicity assessments

Anti-S IgG antibodies against prototype strain (Wuhan) were assessed using validated enzyme-linked immunosorbent assay (ELISA) and nAbs were assessed by validated microneutralization (MN) assay using wild type virus (Ancestral strain: SARS-CoV-2 hCoV-19/Australia/VIC01/2020, GenBank MT007544.1) with an inhibitory concentration of 50% (MN_50_). Seroresponse was defined as 2 fold increase in antibody titers from baseline. Immunogenicity against VoC was assessed in a randomly selected subset of 46 participants for anti-S IgG against Omicron BA.1, and BA.5, and 50 participants for nAbs against Omicron B.1.1.529 (BA.1) from each prime cohort (maintaining 1:1 allocation). In addition, in the same subset of 46 participants, immune responses against Wuhan strain, Omicron BA.1, and BA.5 variants were determined by validated hACE2 receptor binding inhibition assay, as an exploratory objective. CMI responses were measured by quantification of antigen specific T cells using an ex vivo interferon-γ enzyme-linked immune absorbent spot (ELISpot) assay in a randomly selected subset of 18 participants from each of the two prime cohorts maintaining 1:1 allocation.

The lower limit of quantification (LLOQ) for anti-S IgG ELISA assay was 200 ELISA units per mL (EU/mL), with titers below this level documented as 100 EU/mL. The LLOQ for MN assay was a titer of 20, with titers below this level documented as 10. The LLOQ for hACE2 receptor binding inhibition assay was a titer of 10, with titers below this level documented as 5. Details of assays are included in the Supplementary appendix.

Immunogenicity testing was performed in compliance with Good Clinical Laboratory Practice (GCLP) requirements at Novavax, USA (Anti-S IgG and hACE2 receptor binding inhibition assay), at 360biolabs, Australia (nAbs) and at NARI-ICMR, India (CMI).

### Safety assessments

Solicited local and systemic adverse events (AEs) were actively collected for 7 days after vaccination using participant diary cards. The solicited local AEs included injection site pain, tenderness, erythema, swelling, and induration. The solicited systemic AEs included fever, headache, fatigue, malaise, arthralgia, myalgia, nausea, and vomiting. Unsolicited AEs were collected for 28 days after vaccination. Unsolicited AEs were any AEs reported by the participant, observed by the study staff during study visits or those identified during review of medical records or source documents. Serious adverse events (SAEs), and adverse events of special interest (AESIs) including potentially immune-mediated medical condition (PIMMCs) and AESIs relevant to COVID-19 were collected throughout the study.

The severity of all AEs was graded using the Division of AIDS (DAIDS) Table for Grading the Severity of Adult and Pediatric Adverse Events, corrected Version 2.1, July 2017. A subjective grading scale was used to grade the severity of all AEs that were not listed in the DAIDS Table.

Safety was monitored during the study by on-site clinical staff. Periodic reviews of safety data was performed by the protocol safety review team (PSRT). In addition, an independent data safety monitoring board (DSMB) reviewed the safety data and provided oversight on the study.

### Statistical considerations

Total 372 participants were randomized in the study (186 in each prime cohort). Assuming non-evaluable participants ≤ 15% (158 evaluable participants for each cohort), group sample sizes of 93 each in each vaccine group would provide 80% power to detect non-inferiority using a one-sided, two-sample t-test, with a true ratio of the means of 1.00, one-sided significance level (alpha) of 0.025 and coefficients of variation of 1.1. Non-inferiority of SII-NVX-CoV2373 booster over the control vaccine for each cohort was to be concluded separately if the lower limit of the two-sided 95% confidence interval (CI) for the geometric mean ELISA units (GMEU) ratio for anti-S IgG and geometric mean titers (GMT) ratio for nAbs between SII-NVX-CoV2373 and the control vaccine at day 29 was > 0.67 (non-inferiority margin). The same sample size provided 82% power to demonstrate non-inferiority in terms of difference in proportion of participants with seroresponse (anti-S IgG and nAbs) between SII-NVX-CoV2373 and the control vaccine at day 29, using a one-sided, two-sample z-test at alpha level of 0.025. Proportion of participants achieving seroresponse for control arm was assumed 95% and the margin of non-inferiority was −10%. The true difference of the proportion was assumed to be 0. Sample size calculations were performed using a non-inferiority test for the ratio of two means in PASS 15.0.7 Version software.

Analysis of Covariance (ANCOVA) was fitted to the log transformed anti-S IgG or nAbs for vaccine group, log baseline titer, age, sex, duration between first and second dose of primary immunization, duration between second dose to booster dose. Individual mean and 95% CI by treatment group from this model were used to estimate GMTs/GMEUs and geometric mean ratio (GMR) with 95% CI by back transforming to the original scale for non-inferiority comparison at day 29. The unadjusted GMTs/GMEUs were also summarized. The difference between the vaccine groups in the proportion of the participants with seroresponse along with two-sided 95% CIs were calculated using the Miettinen and Nurminen method.

Enrolled Population was all participants who provided written informed consent, regardless of screening, randomization and treatment status in the study. Safety Population included all participants who received the study vaccines. Per Protocol population consisted of all participants who received the study vaccine, provided an evaluable serum sample post vaccination for at least one assessment, had baseline (day 1) immunogenicity data available, excluding any data from time points following a SARS-CoV-2 infection or major protocol deviation before day 29. This was the primary population for immunogenicity analyses. Full Analysis Population comprised of all participants who received the study vaccine and provided an evaluable serum sample post vaccination for at least one assessment.

Continuous data was described using descriptive statistics (n, mean, standard deviation, median, minimum, and maximum). Categorical data was described using the participant count and percentage for each category.

All statistical analyses were performed using SAS^®^ software version 9.4.

### Ethical considerations

The study was approved by the Indian regulatory authority and the Institutional Ethics Committees (IECs) of each of the eight participating study sites. The IECs approving the study were Jamia Hamdard Institutional Ethics Committee, Delhi; Institutional Ethics Committee Bharati Vidyapeeth Deemed University, Pune; Ethics Committee Dr. D. Y. Patil Vidyapeeth, Pune; Institutional Ethics Committee-KIMS, Kalinga Institute of Medical Sciences, Bhubaneshwar; Noble Hospital Institutional Ethics Committee, Pune; Institutional Ethics Committee JSS Medical College, Mysuru; KEM Hospital Research Centre Ethics Committee, Pune; and Institutional Human Ethics Committee All India Institute of Medical Sciences, Gorakhpur. The study was conducted in compliance with Declaration of Helsinki (Revised Fortaleza, 2013) and the International Council for Harmonization (ICH) GCP Guideline, E6 R2 (2016).

## Results

### Disposition of participants

#### ChAdOx1 nCoV-19 prime cohort

**Figure 1:**
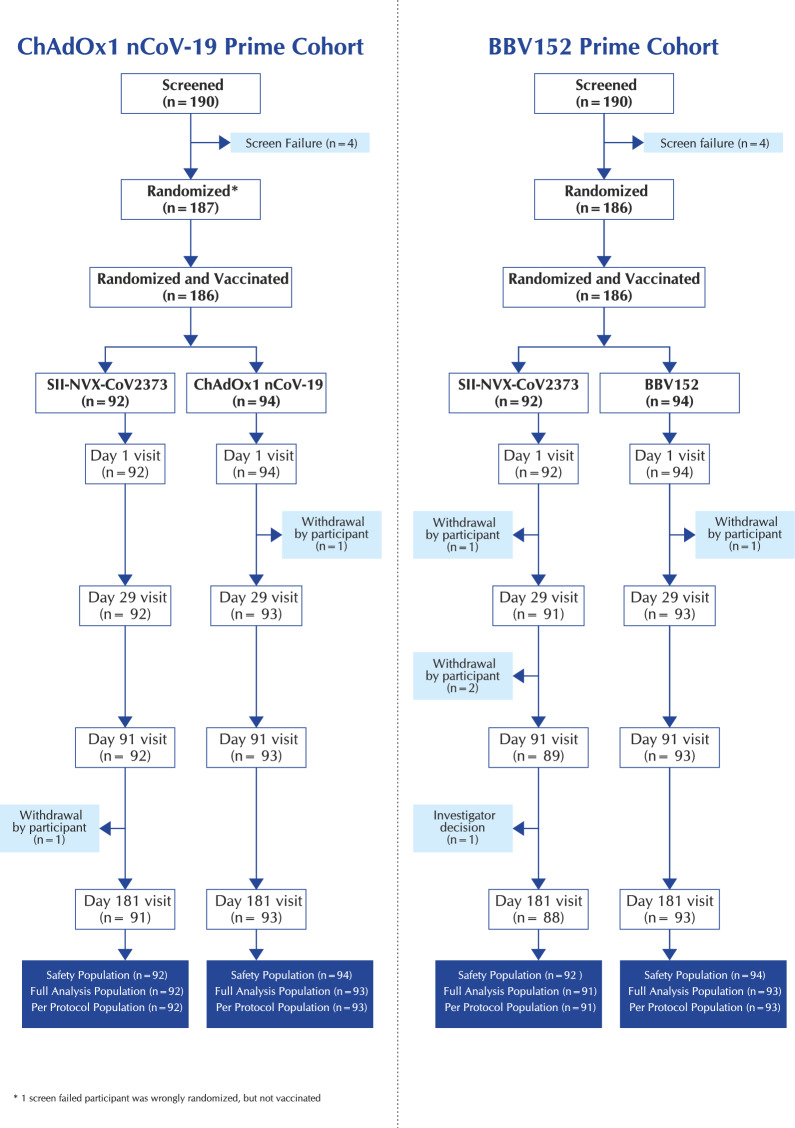
CONSORT flow chart

A total of 190 participants were enrolled and 187 were randomized. Of these, 186 received the study vaccine (92 SII-NVX-CoV2373 and 94 ChAdOx1 nCoV-19). Only a single participant withdrew the consent before day 29. The safety population comprised 186 participants, both full analysis and per protocol populations comprised 185 participants (Fig. 1).

The demographic and baseline characteristics between the groups were comparable. Mean age was about 36 years and 45% were males. About 15% participants had comorbidity. None of the participants was SARS CoV-2 RT-PCR positive. Median duration between two doses of prime series of ChAdOx1 nCoV-19 was 90 days and that between second dose of prime series and the booster dose was 270 days. The proportion of participants with seropositivity for anti-S IgG or nAbs at baseline was comparable between the groups (Table 1).

**Table 1:** Demographics and baseline characteristics - safety population.

Parameter	ChAdOx1 nCoV-19 prime cohort		BBV152 prime cohort	
	SII-NVX-CoV2373 (N = 92)	ChAdOx1 nCoV-19 (N = 94)	SII-NVX-CoV2373 (N = 92)	BBV152 (N = 94)
Age (Years), Mean (SD)	36.0 (11.04)	35.7 (9.56)	38.0 (14.13)	35.8 (14.44)
Age Group (Years), n (%)				
18–59	90 (97.8)	94 (100)	83 (90.2)	85 (90.4)
> = 60	2 (2.2)	0	9 (9.8)	9 (9.6)
Sex, n (%)				
Male	41 (44.6)	43 (45.7)	64 (69.6)	56 (59.6)
Female	51 (55.4)	51 (54.3)	28 (30.4)	38 (40.4)
SARS-CoV-2 RT-PCR Result, n (%)				
Positive	0	0	0	0
Negative	92 (100)	94 (100)	92 (100)	94 (100)
Co-morbidities, n (%)				
Yes	12 (13.0)	16 (17.0)	17 (18.5)	12 (12.8)
No	80 (87.0)	78 (83.0)	75 (81.5)	82 (87.2)
Duration between two doses of prime vaccine (Days) Median (Min, Max)	87.00 (29.0, 285.0)	93.00 (30.0, 248.0)	35.50 (29.0, 214.0)	35.50 (29.0, 184.0)
Duration between Last dose of primary vaccination and booster vaccine (Days) Median (Min, Max)	261.50 (182.0, 606.0)	273.00 (186.0, 548.0)	295.50 (182.0, 417.0)	299.50 (182.0, 416.0)

#### BBV152 prime cohort

A total of 190 participants were enrolled and 186 were randomized and received the study vaccine (92 SII-NVX-CoV2373 and 94 BBV152). Two participants withdrew consent before day 29. The safety population comprised 186 participants, both full analysis and per protocol populations comprised 184 participants (Fig. 1).

The demographic and baseline characteristics between the groups were comparable. Mean age was about 37 years and about 64% were males. About 15% participants had comorbidity. None of the participants was SARS CoV-2 RT-PCR positive. Median duration between two doses of prime series of BBV152 was 35 days and that between second dose of prime series and the booster dose was 298 days. The seropositivity for anti-S IgG and nAbs at baseline was comparable between the groups (Table 1).

### Immunogenicity results

#### ChAdOx1 nCoV-19 prime cohort

**Figure 2:**
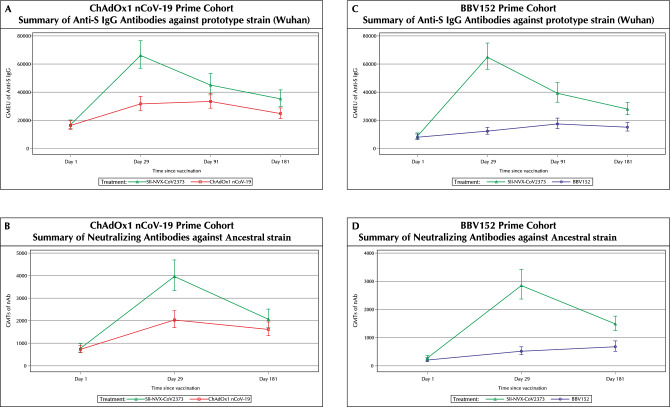
Summary of Anti-S IgG and Neutralizing Antibodies (nAb) at each visit (Per Protocol Population). *GMEU* Geometric mean ELISA unit, *GMT* Geometric mean titer. For each study vaccine, the GMEU of Anti-S IgG and GMT of nAbs and 95% CI were calculated by transforming to the original scale of log_10_-transformed mean and its two-sided 95% CI limits at each visit.

*Anti-S IgG:* Baseline GMEUs of anti-S IgG against prototype strain were comparable between the SII-NVX-CoV2373 and the ChAdOx1 nCoV-19 groups. At day 29, there was a 3.9 fold-rise (95% CI 3.4, 4.5) from the baseline in the SII-NVX-CoV2373 group [GMEU 66,085.8 (95% CI 57,028.0, 76,582.4)] and 1.9 fold-rise (95% CI 1.7, 2.3) from the baseline in the ChAdOx1 nCoV-19 group [GMEU 31,673.6 (95% CI 27,149.5, 36,951.6)] (Fig. 2 and Table S1). Non-inferiority was met with 1.73 as the lower bound of 95% CI (GMEU ratio 2.05 [95% CI 1.73, 2.43]) (Table 2). At day 181, there was a 2.1 fold-rise (95% CI 1.7, 2.5) from the baseline in the SII-NVX-CoV2373 group [GMEU 35,232.4 (95% CI 29,794.0, 41,663.5)] and 1.5 fold-rise (95% CI 1.3, 1.8) from the baseline in the ChAdOx1 nCoV-19 group [GMEU 24,802.7 (95% CI 21,311.5, 28,865.7)] (Table S1).

**Table 2:** Non-inferiority of SII-NVX-CoV2373 heterologous booster to homologous booster in terms for Anti-S IgG and neutralizing antibodies at Visit 2-Day 29 - Per Protocol Population.

	ChAdOx1 nCoV-19 Prime Cohort		BBV152 prime cohort	
	SII-NVX-CoV2373 (N = 92)	ChAdOx1 nCoV-19 (N = 93)	SII-NVX-CoV2373 (N = 91)	BBV152 (N = 93)
Anti-S IgG				
GMEU [1] with 95% CI	65,550.37 (58,155.85, 73,885.11)	31,945.42 (28,366.25, 35,976.20)	65,155.31 (56,348.14, 75,339.04)	12,719.72 (11,063.37, 14,624.05)
GMEU Ratio (GMR) [1] with 95% CI	2.05 (1.73, 2.43)		5.12 (4.20, 6.24)	
Seroresponse				
n (%) 95% CI	80 (86.96) (78.32, 93.07)	35 (37.63) (27.79, 48.28)	86 (94.51) (87.64, 98.19)	19 (20.43) (12.77, 30.05)
Difference in Proportions 95% CI	49.32 (36.49, 60.45)		74.08 (63.24, 82.17)	
Neutralizing antibodies				
GMT [1] 95% Confidence Interval	3883.22 (3363.26, 4483.56)	2050.29 (1777.55, 2364.88)	2654.90 (2221.75, 3172.49)	553.59 (466.46, 657.00)
GMT Ratio (GMR) [1] 95% Confidence Interval	1.89 (1.55, 2.32)		4.80 (3.76, 6.12)	
Seroresponse				
n (%) 95% CI	87 (94.57) (87.77, 98.21)	74 (79.57) (69.95, 87.23)	90 (98.90) (94.03, 99.97)	69 (74.19) (64.08, 82.71)
Difference in Proportions 95% CI	15.00 (5.65, 25.05)		24.71 (16.26, 34.62)	

*LS* least squares, *GMT* geometric mean titer, *GMR *geometric mean ratio.

[1] ANCOVA results, LS Mean and it's 95% CI values by treatment were used to generate the GMEU/GMT and 95% CI and the differences in LS Means and corresponding 95% CI limits were used to obtain GMEU/GMT Ratio and 95% CI using back transforming to the original scale.

ANCOVA model includes vaccine group, log baseline titer, age, sex, duration between first and second dose of the prime vaccine, duration between second dose of the prime vaccine to booster dose of the study vaccine.

The 95% CIs for seroresponse for each vaccine group were calculated by using the Clopper-Pearson method.

Estimate of difference in proportion along with associated 95% CI was obtained using Miettinen and Nurminen method.

There was 86.96% (95% CI 78.32, 93.07) and 37.63% (95% CI 27.79, 48.28) seroresponse at day 29 in the SII-NVX-CoV2373 and ChAdOx1 nCoV-19 groups, respectively. The difference in seroresponse between the groups was highly significant. Non-inferiority was met with 36.49% as the lower bound of 95% CI (Difference in proportion 49.32 [95% CI 36.49, 60.45]) (Table 2). At day 181, seroresponse rate was 57.78% (95% CI 46.91, 68.12) and 30.11% (95% CI 21.03, 40.50) in the SII-NVX-CoV2373 and ChAdOx1 nCoV-19 groups, respectively [Table S2].

*nAbs:* Baseline GMTs of nAbs against ancestral strain were comparable between the SII-NVX-CoV2373 and the ChAdOx1 nCoV-19 groups. At day 29, there was a 5.1 fold-rise (95% CI 4.2, 6.2) from the baseline in the SII-NVX-CoV2373 group [GMT 3963.0 (95% CI 3343.8, 4696.7)] and 2.8 fold-rise (95% CI 2.3, 3.4) in the ChAdOx1 nCoV-19 group [GMT 2031.9 (95% CI 1690.6, 2442.1)] (Fig. 2 and Table S3). Non-inferiority was met with 1.55 as the lower bound of 95% CI (GMT ratio 1.89 [95% CI 1.55, 2.32]) (Table 2). At day 181, there was a 2.7 fold-rise (95% CI 2.1, 3.4) from the baseline in the SII-NVX-CoV2373 group [GMT 2063.4 (95% CI 1693.0, 2514.8)] and 2.2 fold-rise (95% CI 1.8, 2.7) from the baseline in the ChAdOx1 nCoV-19 group [GMT 1612.7 (95% CI 1337.0, 1945.2)] [Table S3].

There was 94.57% (95% CI 87.77, 98.21) and 79.57% (95% CI 69.95, 87.23) seroresponse at day 29 in the SII-NVX-CoV2373 and ChAdOx1 nCoV-19 groups, respectively. The difference in seroresponse between the groups was significant. Non-inferiority was met with 5.65% as the lower bound of 95% CI (Difference in proportion 15.00 [95% CI 5.65, 25.05]) (Table 2). At day 181, seroresponse rate was 70.00% (95% CI 59.43, 79.21) and 72.04% (95% CI 61.78, 80.86) in the SII-NVX-CoV2373 and ChAdOx1 nCoV-19 groups, respectively [Table S4].

#### BBV152 prime cohort

*Anti-S IgG*: Baseline GMEUs of anti-S IgG against prototype strain were comparable between the SII-NVX-CoV2373 and the BBV152 groups. At day 29, there was a 7.4 fold-rise (95% CI 5.9, 9.1) from the baseline in the SII-NVX-CoV2373 group [GMEU 64,868.7 (95% CI 56,178.7, 74,903.1)] and 1.5 fold-rise (95% CI 1.3, 1.8) from the baseline in the BBV152 group [GMEU 12,344.1 (95% CI 10,137.5, 15,031.1)] (Fig. 2 and Table S1). Non-inferiority was met with 4.20 as the lower bound of 95% CI (GMEU ratio 5.12 [95% CI 4.20, 6.24]) (Table 2). At day 181, there was a 3.1 fold-rise (95% CI 2.4, 3.9) from the baseline in the SII-NVX-CoV2373 group [GMEU 27,998.2 (95% CI 24,005.7, 32,654.7)] and 1.9 fold-rise (95% CI 1.5, 2.3) from the baseline in the BBV152 group [GMEU 14,968.4 (95% CI 12,261.4, 18,272.9)] (Table S1).

There was 94.51% (95% CI 87.64, 98.19) and 20.43% (95% CI 12.77, 30.05) seroresponse at day 29 in the SII-NVX-CoV2373 and BBV152 groups, respectively. The difference in seroresponse between the groups was highly significant. Non-inferiority was met with 63.24% as the lower bound of 95% CI (Difference in proportion 74.08 [95% CI 63.24, 82.17]) (Table 2). At day 181, seroresponse rate was 56.82% (95% CI 45.82, 67.34) and 39.13% (95% CI 29.12, 49.86) in the SII-NVX-CoV2373 and BBV152 groups, respectively (Table S2).

*nAbs:* Baseline GMTs of nAbs against ancestral strain were comparable between the SII-NVX-CoV2373 and the BBV152 groups. At day 29, there was a 10.4 fold-rise (95% CI 7.9, 13.5) from the baseline in the SII-NVX-CoV2373 group [GMT 2848.1 (95% CI 2370.0, 3422.6)] and 2.5 fold-rise (95% CI 2.1, 3.1) in the BBV152 group [GMT 515.6 (95% CI 395.4, 672.3)] (Fig. 2 and Table S3). Non-inferiority was met with 3.76 as the lower bound of 95% CI of the GMT ratio (GMT ratio 4.80 [95% CI 3.76, 6.12]) (Table 2). At day 181, there was a 5.3 fold-rise (95% CI 3.9, 7.2) from the baseline in the SII-NVX-CoV2373 group [GMT 1486.6 (95% CI 1253.9, 1762.5)] and 3.3 fold-rise (95% CI 2.6, 4.2) from the baseline in the BBV152 group [GMT 664.6 (95% CI 505.9, 873.0)] [Table S3].

There was 98.90% (95% CI 94.03, 99.97) and 74.19% (95% CI 64.08, 82.71) seroresponse at day 29 in the SII-NVX-CoV2373 and BBV152 groups, respectively. The difference in seroresponse between the groups was significant. Non-inferiority was met with 16.26% as the lower bound of 95% CI of the difference in proportion of seroresponse (Difference in proportion 24.71 [95% CI 16.26, 34.62]) (Table 2). At day 181, seroresponse rate was 79.55% (95% CI 69.61, 87.40) and 71.74% (95% CI 61.39, 80.64) in the SII-NVX-CoV2373 and BBV152 groups, respectively (Table S4).

#### nAbs and anti-S IgG against VoC

**Figure 3:**
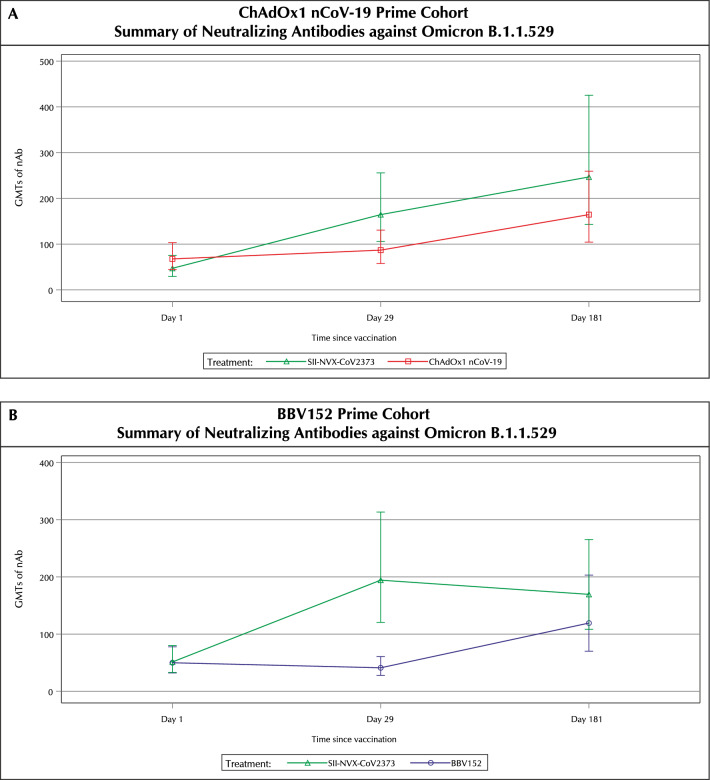
Summary of Neutralizing Antibodies (nAb) against Omicron B.1.1.529 at each visit (Per protocol population). GMT: Geometric Mean Titer. For each study vaccine, the GMT of Neutralizing Antibodies (nAb) and 95% CI were calculated by transforming to the original scale of log_10_-transformed mean and its two-sided 95% CI limits at each visit.

In the ChAdOx1 nCoV-19 prime cohort at day 29, SII-NVX-CoV2373 booster induced 3.5 fold-rise (95% CI 2.3, 5.3) in GMTs and 84% (95% CI 63.92, 95.46) seroresponse rate for nAbs against Omicron B.1.1.529 (BA.1), while a 1.3 fold-rise (95% CI 1.0, 1.7) in GMTs and 40% (95% CI 21.13, 61.33) seroresponse rate was seen with the ChAdOx1 nCoV-19 booster (Fig. 3, Tables S3 and S4). At day 181, there was 5.5 fold-rise (95% CI 2.9, 10.4) in GMTs and 75% (95% CI 53.29, 90.23) seroresponse rate in the SII-NVX-CoV2373 group while 2.4 fold-rise (95% CI 1.6, 3.8) in GMTs and 72% (95% CI 50.61, 87.93) seroresponse rate in the ChAdOx1 nCoV-19 group (Tables S3 and S4).

In the BBV152 prime cohort at day 29, SII-NVX-CoV2373 booster induced 3.8 fold-rise (95% CI 2.3, 6.3) in GMTs and 72% (95% CI 50.61, 87.93) seroresponse rate for nAbs against Omicron B.1.1.529 while there was no rise in GMTs and only 24% (95% CI 9.36, 45.13) seroresponse rate with BBV152 booster (Fig. 3, Tables S3 and S4). At day 181, there was 3.3 fold-rise (95% CI 1.7, 6.5) in GMTs and 62.5% (95% CI 40.59, 81.20) seroresponse rate in the SII-NVX-CoV2373 group while 2.3 fold-rise (95% CI 1.4, 3.8) in GMTs and 53.85% (95% CI 33.37, 73.41) seroresponse rate in the BBV152 group (Tables S3 and S4).

**Figure 4:**
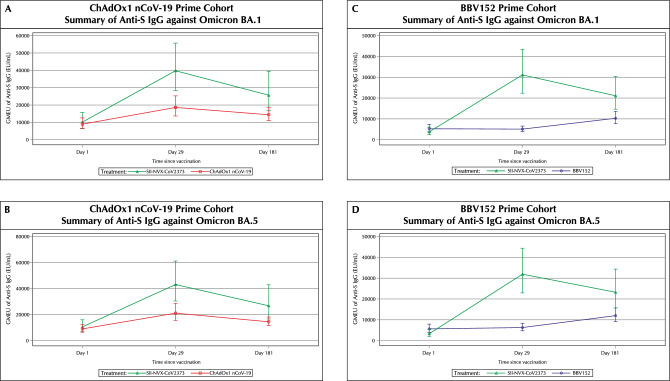
Summary of Anti-S IgG against Omicron BA.1 and Omicron BA.5 at each visit (Per protocol population). *GMEU* Geometric Mean ELISA Unit. For each study vaccine, the GMEU of Anti-S IgG and 95% CI were calculated by transforming to the original scale of log_10_-transformed mean and its two-sided 95% CI limits at each visit.

In the ChAdOx1 nCoV-19 prime cohort at day 29, SII-NVX-CoV2373 booster induced 3.9 fold-rise (95% CI 2.7, 5.6) in GMEUs and 73.91% (95% CI 51.59, 89.77) seroresponse rate for anti-S IgG against Omicron BA.1 and 4.1 fold-rise (95% CI 2.9, 5.7) in GMEUs and 86.96% (95% CI 66.41, 97.22) seroresponse rate against Omicron BA.5. ChAdOx1 nCoV-19 booster induced 2.1 fold-rise (95% CI 1.4, 3.0) in GMEUs and 43.48% (95% CI 23.19, 65.51) seroresponse rate for anti-S IgG against Omicron BA.1 and 2.4 fold-rise (95% CI 1.7, 3.3) in GMEUs and 47.83% (95% CI 26.82, 69.41) seroresponse rate against Omicron BA.5. (Fig. 4 and Tables S1 and S2). At day 181, there was 2.6 fold-rise (95% CI 1.4, 5.0) in GMEUs and 50% (95% CI 28.22, 71.78) seroresponse rate against Omicron BA.1 and 2.6 fold-rise (95% CI 1.4, 4.8) in GMEUs and 54.55% (95% CI 32.21, 75.61) seroresponse rate against Omicron BA.5 in the SII-NVX-CoV2373 group. At day 181, there was 1.6 fold-rise (95% CI 1.1, 2.3) in GMEUs and 43.48% (95% CI 23.19, 65.51) seroresponse rate against Omicron BA.1 and 1.6 fold-rise (95% CI 1.2, 2.2) in GMEUs and 39.13% (95% CI 19.71, 61.46) seroresponse rate against Omicron BA.5 in the ChAdOx1 nCoV-19 group (Tables S1 and S2).

In the BBV152 prime cohort at day 29, SII-NVX-CoV2373 booster induced 8.3 fold-rise (95% CI 5.1, 13.3) in GMEUs and 91.3% (95% CI 71.96, 98.93) seroresponse rate for anti-S IgG against Omicron BA.1 and 9.6 fold-rise (95% CI 5.9, 15.8) in GMEUs and 95.65% (95% CI 78.05, 99.89) seroresponse rate against Omicron BA.5; while there was no rise in GMEUs and 4.35% (95% CI 0.11, 21.95) seroresponse rate against Omicron BA.1 and BA.5 seen with BBV152 booster (Fig. 4 and Tables S1 and S2). At day 181, there was 5.8 fold-rise (95% CI 3.0, 11.2) in GMEUs and 77.27% (95% CI 54.63, 92.18) seroresponse rate against Omicron BA.1 and 7.3 fold-rise (95% CI 3.8, 14.1) in GMEUs and 77.27% (95% CI 54.63, 92.18) seroresponse rate against Omicron BA.5 in the SII-NVX-CoV2373 group. At day 181, there was 2 fold-rise (95% CI 1.3, 2.9) in GMEUs and 56.52% (95% CI 34.49, 76.81) seroresponse rate against Omicron BA.1 and 2.1 fold-rise (95% CI 1.5, 3.1) in GMEUs and 56.52% (95% CI 34.49, 76.81) seroresponse rate against Omicron BA.5 in the BBV152 group (Tables S1 and S2).

#### hACE2 receptor binding inhibition antibodies

In the ChAdOx1 nCoV-19 prime cohort at day 29, SII-NVX-CoV2373 booster induced 3.1 fold-rise (95% CI 2.2, 4.3), 3.3 fold-rise (95% CI 2.5, 4.5), and 3.3 fold-rise (95% CI 2.4, 4.5) in GMTs against Wuhan, Omicron BA.1 and Omicron BA.5, respectively, while ChAdOx1 nCoV-19 booster induced 2 fold-rise (95% CI 1.5, 2.6), 2 fold-rise (95% CI 1.4, 2.7), and 1.8 fold-rise (95% CI 1.2, 2.7) in GMTs, respectively. The seroresponse rate was 69% (95% CI 47.08, 86.79), 78.26% (95% CI 56.30, 92.54) and 78.26% (95% CI 56.30, 92.54) against Wuhan, Omicron BA.1 and Omicron BA.5, respectively with the SII-NVX-CoV2373 booster at day 29, while it was 39.13% (95% CI 19.71, 61.46), 43.48% (95% CI 23.19, 65.51) and 39.13% (95% CI 19.71, 61.46), respectively with the ChAdOx1 nCoV-19 booster. At day 181, there was 2 fold-rise (95% CI 1.0, 3.9), 3.7 fold-rise (95% CI 1.9, 7.1), and 3.4 fold-rise (95% CI 1.8, 6.3) in GMTs against Wuhan, Omicron BA.1 and Omicron BA.5, respectively, in the SII-NVX-CoV2373 group, while there was 1.4 fold-rise (95% CI 1.0, 2.0), 2.4 fold-rise (95% CI 1.5, 3.8), and 2 fold-rise (95% CI 1.3, 3.1) in GMTs, respectively, in the ChAdOx1 nCoV-19 group. At day 181, in the SII-NVX-CoV2373 group seroresponse rate was 45.45% (95% CI 24.39, 67.79), 63.64% (95% CI 40.66, 82.80) and 59.09% (95% CI 36.35, 79.29) against Wuhan, Omicron BA.1 and Omicron BA.5, respectively, while in the ChAdOx1 nCoV-19 group it was 26.09% (95% CI 10.23, 48.41), 52.17% (95% CI 30.59, 73.18) and 52.17% (95% CI 30.59, 73.18), respectively (Tables S5 and S6).

In BBV152 prime cohort, SII-NVX-CoV2373 booster induced 9.7 fold-rise (95% CI 5.9, 15.9), 7.1 fold-rise (95% CI 4.4, 11.4), and 7 fold-rise (95% CI 4.3, 11.3) in GMTs against Wuhan, Omicron BA.1 and Omicron BA.5, respectively, while there was no rise in GMTs against any of these strains with BBV152 booster. The seroresponse rate was 100% (95% CI 85.18, 100.00), 91.30% (95% CI 71.96, 98.93) and 86.96% (95% CI 66.41, 97.22) against Wuhan, Omicron BA.1 and Omicron BA.5, respectively in the SII-NVX-CoV2373 group, while it was 13.04% (95% CI 2.78, 33.59) and 4.35% (95% CI 0.11, 21.95) against Omicron BA.1 and Omicron BA.5, respectively in the BBV152 group with no seroresponse seen against Wuhan. At day 181, in the SII-NVX-CoV2373 group seroresponse rate was 72.73% (95% CI 49.78, 89.27), 77.27% (95% CI 54.63, 92.18) and 72.73% (95% CI 49.78, 89.27) against Wuhan, Omicron BA.1 and Omicron BA.5, respectively, while in the BBV152 group it was 34.78% (95% CI 16.38, 57.27), 73.91% (95% CI 51.59, 89.77) and 65.22% (95% CI 42.73, 83.62), respectively (Tables S5 and S6).

### CMI response

IFN-γ secreting T-Cell response to SARS-CoV-2 spike protein: There was increase in T cells responses at days 29 and 181 in each group in both ChAdOx1 nCoV-19 and BBV152 prime cohorts (Table S7).

### Safety results

#### SAEs and AESIs

Two SAEs were reported in the SII-NVX-CoV2373 group (BBV152 prime cohort) - lower respiratory tract infection and gastroenteritis with dehydration, both unrelated to the study vaccine. No AESI was reported.

#### Unsolicited AEs

*ChAdOx1 nCoV-19 prime cohort*: Only a single unsolicited AE (1.1%) was reported in the SII-NVX-CoV2373 group and 6 unsolicited AEs in 4 participants (4.3%) in the ChAdOx1 nCoV-19 group and none was treatment-related. All AEs were of mild severity (except for 1 event of joint injury in the SII-NVX-CoV2373 group of moderate severity) and resolved without any sequelae.

#### BBV152 prime cohort

A total of 4 unsolicited AEs in 4 participants (5.4%) were reported in the SII-NVX-CoV2373 group and 6 unsolicited AEs in 5 participants (5.3%) in the BBV152 group. None was treatment-related in the SII-NVX-CoV2373 group and 2 events in the BBV152 group were treatment-related (diarrhoea and injection site pain). All AEs were of mild to moderate severity (except for 1 severe event of hypertension in the BBV152 group) and resolved without any sequelae.

There were no AEs which led to study discontinuation. There was only one case of COVID-19 at 3 months after the booster in the BBV152 group which was mild in severity, and resolved without any sequelae.

### Solicited AEs

#### ChAdOx1 nCoV-19 prime cohort

There were 69 solicited AEs in 33 participants (35.9%) in the SII-NVX-CoV2373 group and 61 solicited AEs in 29 participants (30.9%) in the ChAdOx1 nCoV-19 group. The most common local solicited AEs were injection site pain, tenderness, and swelling (Table 3). The common systemic solicited AEs were headache, arthralgia, fatigue, malaise, myalgia, and fever (Table 3). Almost all AEs were of mild severity and all resolved without any sequelae.

**Table 3:** Summary of solicited adverse events safety population.

	ChAdOx1 nCoV-19 Prime cohort		BBV152 Prime cohort	
	SII-NVX-CoV2373 (N = 92) n (%) [E]	ChAdOx1 nCoV-19 (N = 94) n (%) [E]	SII-NVX-CoV2373 (N = 92) n (%) [E]	BBV152 (N = 94) n (%) [E]
Participants with at Least One Solicited Adverse Event	33 (35.9) [69]	29 (30.9) [61]	34 (37.0) [84]	43 (45.7) [87]
Participants with at Least One Local Solicited AE	18 (19.6) [26]	24 (25.5) [28]	20 (21.7) [29]	36 (38.3) [43]
Injection Site Pain	17 (18.5) [17]	24 (25.5) [24]	20 (21.7) [20]	35 (37.2) [35]
Injection Site Tenderness	5 (5.4) [5]	3 (3.2) [3]	4 (4.3) [4]	6 (6.4) [6]
Injection Site Swelling	3 (3.3) [3]	0	3 (3.3) [3]	1 (1.1) [1]
Injection Site Erythema	1 (1.1) [1]	0	1 (1.1) [1]	0
Injection Site Induration	0	1 (1.1) [1]	1 (1.1) [1]	1 (1.1) [1]
Participants with at Least One Systematic Solicited AE	24 (26.1) [43]	17 (18.1) [33]	25 (27.2) [55]	25 (26.6) [44]
Headache	12 (13.0) [12]	11 (11.7) [11]	13 (14.1) [13]	9 (9.6) [9]
Arthralgia	7 (7.6) [7]	6 (6.4) [6]	5 (5.4) [5]	5 (5.3) [5]
Fatigue	7 (7.6) [7]	6 (6.4) [6]	11 (12.0) [11]	11 (11.7) [11]
Malaise	5 (5.4) [5]	4 (4.3) [4]	12 (13.0) [12]	7 (7.4) [7]
Myalgia	5 (5.4) [5]	3 (3.2) [3]	7 (7.6) [7]	5 (5.3) [5]
Fever	5 (5.4) [5]	0	4 (4.3) [4]	4 (4.3) [4]
Vomiting	1 (1.1) [1]	2 (2.1) [2]	1 (1.1) [1]	1 (1.1) [1]
Nausea	1 (1.1) [1]	1 (1.1) [1]	2 (2.2) [2]	2 (2.1) [2]

*n* number of participants, *E* number of events.

#### BBV152 prime cohort

There were 84 solicited AEs in 34 participants (37%) in the SII-NVX-CoV2373 group and 87 solicited AEs in 43 participants (45.7%) in the BBV152 group. The most common local solicited AEs were injection site pain, tenderness, and swelling (Table 3). The common systemic solicited AEs were fatigue, headache, malaise, myalgia, arthralgia, and fever (Table 3). Almost all AEs (98%) were of mild severity and all resolved without any sequelae.

## Discussion

This Phase 3 study was conducted to assess heterologous booster effect of SII-NVX-CoV2373 in 372 adults who were primed with ChAdOx1 nCoV-19 or BBV152. The heterologous booster dose of SII-NVX-CoV2373 was non-inferior to homologous booster dose of either ChAdOx1 nCoV-19 or BBV152 in terms of anti-S IgG and nAbs. Also, the SII-NVX-CoV2373 booster showed significantly higher immune responses than BBV152 homologous booster in terms of anti-S IgG, nAbs and hACE2 receptor binding inhibition antibodies against prototype / ancestral strain as well as Omicron variants. All three vaccines were found safe and well tolerated.

The homologous booster dose of COVID-19 vaccine in India started in January 2022 when the at-risk population (elderly, healthcare workers, etc.) were offered a free dose six months after the last dose^24^. The programme opened for 18–59-year age group in April 2022 but it was a paid vaccination. In July 2022, free booster vaccination was started for this group^25^. Our study started in May 2022 and this age group was ineligible to receive free booster dose through programme. As a result, most of the participants were between 18 and 59 years with only 20 participants above 60 years of age in the study. The mean duration from the last dose of prime series and booster dose was around nine months in both the cohorts.

GMTs/GMEUs of both the antibodies with SII-NVX-CoV2373 booster were at around 2 times higher than the ChAdOx1 nCoV-19 booster and > 5 times higher than the BBV152 booster. Even the seroresponse rates with SII-NVX-CoV2373 were numerically higher than those in the ChAdOx1 nCoV-19 and BBV152 groups. In the COV-BOOST study, NVX-CoV2373 was found to show higher immune response as compared to viral vector vaccines and inactivated vaccines^17^. The results in our study are in line with these findings.

There are no head to head efficacy studies but in general, primary immunization with NVX-CoV2373 has been found more protective against symptomatic COVID-19 than viral vector vaccines and inactivated vaccines. The vaccine gave around 90% efficacy^11, 12^, while the same estimate for ChAdOx1 nCoV-19 was around 70%^26^ and around 77% for BBV152^27^. Our study shows that the vaccine is more immunogenic than these two vaccines when given as a booster vaccination. One reason for this difference could be the Matrix M adjuvant in the vaccine which enhances immune response through a combination of activities including recruitment and activation of innate immune cells to the site of vaccine injection, rapid antigen delivery to antigen-presenting cells, and enhanced antigen presentation via both major histocompatibility complex (MHC) I and MHC II molecules in the draining lymph nodes^28^. In general, heterologous booster is speculated to give better immune response than homologous booster as seen in RHH-01 study^29^ in individuals primed with inactivated vaccine, the heterologous booster with mRNA or adeno-vectored vaccine induced a substantially higher immune response as compared to homologous booster of inactivated vaccine. However, it is not always the case as seen in another study^30^, a homologous booster with ChAdOx1 nCoV-19 induced higher immune response as compared to heterologous booster with an inactivated vaccine in individuals with primary ChAdOx1 nCoV-19 vaccination. Similarly, COV-BOOST study in UK reported that the heterologous booster with inactivated vaccine did not induce significant immune response as compared to other homologous or heterologous boosters^17^. Therefore, the higher immune response seen with SII-NVX-CoV2373 in our study may not be only because it was used as a heterologous booster. It is hypothesized that anti-vector immunity may interfere with immunogenicity or efficacy of viral vector vaccine, particularly when given as a homologous booster vaccination^31^. However, this could be an issue more likely with human vector viruses. In one study, anti-S antibody responses increased after a homologous booster with ChAdOx1 nCoV-19 but the anti-vector antibody responses did not^32^.

The real-world evidence from different COVID-19 vaccines (prototype strain) supports the completion of primary series and booster vaccinations where appropriate, especially to restore waning vaccine effectiveness against the more infectious Omicron variant and protect populations from severe outcomes, hospital admissions, and longer lasting post-COVID-19 complications, as well as mortality^33^. Though our study was not powered to assess effectiveness, only one case of mild COVID-19 was reported in BBV152 group even during the strong omicron wave in India, thus implying good protection from the booster doses. As expected there was a decline in both anti-S IgG and nAbs titers against ancestral strain at 6 months in all the groups. On the other hand, nAbs against Omicron variant showed a slight increase in titers at 6 months possibly because of asymptomatic infections. However, anti-S IgG against Omicron showed a decline in titers except in BBV152 group. The reason for these discordant findings is not known.

Neutralizing antibody titers are an accepted measure of immunity against COVID-19 while there is a strong correlation for nAb titers with anti-S IgG and hACE-2 binding inhibition among vaccinated individuals^34^. We also found the similar trend in our study.

It is interesting to note that the baseline titers of both anti-S IgG and nAbs in the ChAdOx1 nCoV-19 prime group were almost two times as compared to the BBV152 prime group even though the intervals since the last vaccination were quite similar. This has been reported earlier that ChAdOx1 nCoV-19 gave higher, more durable antibody response than BBV152^35–38^. Moreover, the post-booster titers in the ChAdOx1 nCoV-19 prime-ChAdOx1 nCoV-19 boost participants were much higher than in the BBV152 prime-BBV152 boost participants. This has also been reported previously^39^. In our study, there were more males in the BBV152 prime cohort than in the ChAdOx1 nCoV-19 prime cohort which could be an incidental finding. However, we do not anticipate any impact of this finding on our results.

Effectiveness of ChAdOx1 nCoV-19 has also been higher than BBV152^40–43^. The same effectiveness data for NVX-CoV2373 booster are not available because the vaccine was approved much later than these two vaccines. As a result, it was not used in the programme in India to a great extent. Higher levels of humoral immune markers are known to correlate with a reduced risk of symptomatic infection^44–46^. Considering this, the higher immune response seen in this study indicates that SII-NVX-CoV2373 booster may give similar or higher effectiveness than ChAdOx1 nCoV-19 and BBV152 booster.

Our study had a few limitations. Our study was unable to assess the immunogenicity among the elderly population with sufficient sample size, as most of this population had already received a booster dose prior to the commencement of the study. This exclusionary factor limited the elderly cohort, constituting only 5% of our study participants. This subset of participants, especially those with comorbidities, might exhibit immunosenescence, thereby adding complexity to our observations. The study did not assess BA.2, BA.3, BA.4, and Delta-variants. We evaluated only one booster dose whereas in some countries second and third boosters have also been introduced particularly for older adults and immunocompromised individuals^47^. This was because in India only one booster dose is recommended. The study was not designed to assess efficacy of the booster, however as mentioned above higher levels of all immune markers are known to correlate with a reduced risk of symptomatic infection^44–46^. Though the efficacy of SII-NVX-CoV2373 has been reported to be higher than ChAdOx1 nCoV-19 and BBV152, the present study was not designed as a superiority trial as this was not necessary for regulatory approval of a heterologous booster dose. In our study, we did not assess the homologous booster dose of SII-NVX-CoV2373 because SII-NVX-CoV2373 was not used for primary immunization in India. However, NVX-CoV2373 has already been evaluated as a homologous booster dose which showed that for both the prototype strain and all variants evaluated, immune responses following the booster were similar to or higher than those associated with high levels of efficacy in phase 3 studies of the vaccine^6^.

A study reported that the heterologous booster induced greater systemic reactogenicity than their homologous counterparts^13^. However, in our study heterologous booster induced numerically higher systemic reactions than homologous booster in ChAdOx1 nCoV-19 prime cohort, while homologous booster induced numerically higher local reactions than heterologous booster in BBV152 prime cohort, though our study was not powered to detect these differences.

In conclusion, a heterologous booster of SII-NVX-CoV2373 in ChAdOx1 nCoV-19 and BBV152-primed adults demonstrated a non-inferior booster response, which was numerically higher than that achieved with a homologous booster of ChAdOx1 nCoV-19 and BBV152. The vaccine also exhibited an acceptable safety profile.

### Supplementary Information

Supplementary Information 1.

Supplementary Information 2.

## Data Availability

The datasets used and/or analysed during the current study are available from the corresponding author on reasonable request after execution of appropriate confidentiality and data transfer agreements. Anonymized study protocol is appended to this paper.
