# Supplementary material for: A Phase 3, randomized, non-inferiority study of a heterologous booster dose of SARS CoV-2 recombinant spike protein vaccine in adults
Source: Sci Rep. 2023 Oct 3;13:16579. doi: 10.1038/s41598-023-43578-w (PMC10547846; doi:10.1038/s41598-023-43578-w)
Supplement: Supplementary file 1 — Supplementary Information 1. [file 41598_2023_43578_MOESM1_ESM.docx]

**Supplementary appendix**

**A Phase 3, randomized, non-inferiority study of a heterologous booster dose of SARS CoV-2 recombinant spike protein vaccine in adults**

**Immunogenicity assessment**

**Anti-S IgG:**

SARS-CoV-2 spike protein serum IgG ELISA was performed at Novavax (Gaithersburg, MD, USA).

Onto the surface of the 96-well microtitre plate wells (100 μl/well) recombinant SARS-CoV-2 (rSARS-CoV-2) S protein was fixed by direct adsorption for 15 to 72 hours at 2°C to 8°C at a concentration of 1 μg/ml in phosphate-buffered saline (PBS). Plates were washed four times with 300 μl/well PBST, blocked with 300 μl blocking buffer for 1 to 1.5 hours at 24°C ± 2°C. Diluted reference standard (2-fold dilution series of 12 dilutions starting 1:1000) and human serum samples (3-fold dilution series of 12 dilutions) in assay buffer (1% milk in PBS) starting at 1:100 dilution were then added in duplicate (100 μl/well) to the rSARS-CoV-2 S protein-coated wells and any specific antibodies were allowed to complex with the coated antigen for 2 hours ± 10 minutes at 24°C ± 2°C. Plates were washed six times with 300 μl/well PBS with Tween® detergent (PBST). Antibodies bound to the rSARS-CoV-2 S protein were then detected using a horseradish peroxidase (HRP) conjugate goat anti-human immunoglobulin G (IgG) antibody diluted 1: 2000 (Southern Biotech cat no. 2040-05) incubated for 1 hour ± 10 minutes at 24°C ± 2°C, washed three times with 300 μl/well PBST, and a colorimetric signal generated by addition of 100 μl/well 3, 3′,5,5′-tetramethylbenzidine (TMB) chromogenic substrate for 10 minutes ± 2 minutes at 24°C ± 2°C. Following the completion of the incubation, 100 μL/well of TMB stop solution was used to stop the TMB reaction. With the use of a 96-well plate reader from Molecular Device, the absorbance was calculated at 450 nm. The optical density (OD) of the chromogenic substrate at the endpoint is proportional to the quantity of anti-rSARS-CoV-2 S IgG present in the blood sample when binding reagents (coated antigen and secondary antibody) are in excess. By comparing the results to a reference standard curve, the total anti-rSARS-CoV-2 S protein IgG antibody level in a serum sample was quantitated in ELISA units, EU/ml. The results were analysed in singleton by SoftMax Pro software using 4-PL curve fit. Control plates comprising of positive controls and negative controls were included in the assay. The qualified and validated assay was used for clinical trial testing.

**Microneutralization (MN) assay**

360Bio, Australia, developed and validated a MN assay to measure a neutralizing antibody response in serum against SARS-CoV-2. Human serum samples were heat inactivated for 30 minutes at 56⁰C and then diluted in MN assay media [Dulbecco Minimal Essential Medium without L-glutamine (DMEM; Thermo Fisher Scientific, Cat. No. 10313-021)] supplemented with 2% fetal bovine serum (FBS) (Bovagen SFBS), 1% GlutaMAX™ (Thermo Fisher Scientific, Cat. No. 35050-061), and 1% Pen/Strep (Thermo Fisher Scientific, Cat. No.15140-122). Serum was diluted 1:10 in assay media in the first column of a 96-well dilution plate, and then an 11-point 2-fold serial dilution was prepared. An equal volume of SARS-CoV-2 (4000 TCID50 units/ml) was mixed with diluted sera, incubated for 1 hour at 37^o^C, 5% CO2. Following this, 100 μl of the virus/serum mixtures (200 TCID50 units/well) were added in duplicate to Vero E6 cells, pre-seeded 24 hours prior in 96-well plates in 100 μl of assay media at 1.5 x 104 cells/well. Plates were incubated for 3 days at 37^o^C, 5% CO2.

The residual non-neutralized virus was detected via cytopathic effect (CPE) by microscopic scoring by a fully trained experienced personnel. Two replicate wells per dilution were scored as either positive (SARS-CoV-2 cytopathology is present) or negative (healthy Vero E6 monolayer). The neutralization titre was expressed as the reciprocal of the highest dilution at which ≥50% of the replicate wells were protected from infection (MN_50_).

**Human Angiotensin-converting enzyme 2 (hACE-2) receptor binding inhibition assay**

Novavax (Gaithersburg, MD, USA) has developed and validated an ELISA method to measure the inhibition of SARS-CoV-2 S protein binding to human angiotensin-converting enzyme 2 (hACE2) receptor by human serum from subjects who were infected with SARS-CoV-2 or vaccinated with SARS-CoV-2 rS in clinical trials and containing antibodies to the SARS-CoV-2 S protein. The hACE2 receptor binding inhibitor is the term used to refer to the molecules which block the binding of the hACE2 receptor to the SARS-CoV-2 S protein.

In this assay, the recombinant S protein was immobilized onto the surface of microtitre plate wells by direct adsorption. The S protein-coated wells were then filled with diluted human serum samples, along with assay quality controls, and any molecules that can bind to the S protein, such as specific antibodies, were allowed to complex with the immobilized S protein. Following a plate-washing procedure, a fixed concentration of hACE2 with a polyhistidine-Tag (His-Tag) was added to the plate for incubation, during which the hACE2 receptors bind to the S proteins that were not complexed with antibodies or other inhibitors in human serum. Then the detection of hACE2 receptor bound to the S protein is performed using a mouse anti-His-Tag/ HRP conjugate, and a colorimetric signal is generated by the addition of 3,3′,5,5′- TMB substrate., The OD of the chromogenic substrate at endpoint is proportional to the quantity of hACE2 His-Tag that are bound to S protein, when binding reagents (S protein coating antigen and secondary antibody/HRP conjugate) are in excess. The amount of bound hACE2 detected is inversely proportional to the amount hACE2 binding inhibitors (antibodies) in human serum.

**Table S1: Summary of Anti-S IgG antibodies against Prototype strain and Variants of Concerns - Per Protocol Population**

| **Timepoint** | **Statistic** | **ChAdOx1 nCoV-19 Prime Cohort** | | **BBV152 Prime Cohort** | |
| --- | --- | --- | --- | --- | --- |
|  |  | **SII-NVX-CoV2373 (N=92)** | **ChAdOx1 nCoV-19 (N=93)** | **SII-NVX-CoV2373 (N=91)** | **BBV152 (N=93)** |
| **Anti-S IgG - Wuhan** | | | | | |
| Visit 1 - Day 1 (Baseline) | n | 92 | 93 | 91 | 93 |
|  | GMEU  (95% CI) | 16870.1  (14053.1, 20251.8) | 16378.4  (13610.2, 19709.7) | 8811.9  (6990.4, 11108.2) | 8001.5  (6243.4, 10254.7) |
| Visit 2 – Day 29 | n | 92 | 93 | 91 | 93 |
|  | GMEU  (95% CI) | 66085.8  (57028.0, 76582.4) | 31673.6  (27149.5, 36951.6) | 64868.7  (56178.7, 74903.1) | 12344.1  (10137.5, 15031.1) |
|  | GMFR (95% CI) | 3.9 (3.4, 4.5) | 1.9 (1.7, 2.3) | 7.4 (5.9, 9.1) | 1.5 (1.3, 1.8) |
| Visit 3 - Day 91 | n | 91 | 93 | 89 | 92 |
|  | GMEU  (95% CI) | 45041.9  (38099.7, 53249.0) | 33421.6  (28617.0, 39032.9) | 39233.4  (32879.2, 46815.6) | 17515.8  (14142.6, 21693.5) |
|  | GMFR (95% CI) | 2.7 (2.2, 3.2) | 2.0 (1.7, 2.5) | 4.3 (3.4, 5.4) | 2.2 (1.7, 2.8) |
| Visit 4 - Day 181 | n | 90 | 93 | 88 | 92 |
|  | GMEU  (95% CI) | 35232.4  (29794.0, 41663.5) | 24802.7  (21311.5, 28865.7) | 27998.2  (24005.7, 32654.7) | 14968.4  (12261.4, 18272.9) |
|  | GMFR (95% CI) | 2.1 (1.7, 2.5) | 1.5 (1.3, 1.8) | 3.1 (2.4, 3.9) | 1.9 (1.5, 2.3) |
| **Anti-S IgG - Omicron BA.1** | | | | | |
| Visit 1 - Day 1 (Baseline) | n | 23 | 23 | 23 | 23 |
|  | GMEU  (95% CI) | 10149.6  (6579.2, 15657.6) | 8943.0  (6365.5, 12564.1) | 3772.4  (2358.3, 6034.5) | 5253.7  (3736.1, 7387.7) |
| Visit 2 – Day 29 | n | 23 | 23 | 23 | 23 |
|  | GMEU  (95% CI) | 39758.2  (28381.2, 55695.8) | 18595.6  (13678.3, 25280.6) | 31142.0  (22349.8, 43392.9) | 5080.1  (3954.1, 6526.8) |
|  | GMFR (95% CI) | 3.9 (2.7, 5.6) | 2.1 (1.4, 3.0) | 8.3 (5.1, 13.3) | 1.0 (0.8, 1.1) |
| Visit 4 - Day 181 | n | 22 | 23 | 22 | 23 |
|  | GMEU  (95% CI) | 25658.5  (16669.8, 39494.2) | 14388.4  (11060.9, 18716.7) | 21098.9  (14643.3, 30400.4) | 10285.4  (7751.8, 13647.1) |
|  | GMFR (95% CI) | 2.6 (1.4, 5.0) | 1.6 (1.1, 2.3) | 5.8 (3.0, 11.2) | 2.0 (1.3, 2.9) |
| **Anti-S IgG - Omicron BA.5** | | | | | |
| Visit 1 - Day 1 (Baseline) | n | 23 | 23 | 23 | 23 |
|  | GMEU  (95% CI) | 10529.0  (6925.9, 16006.5) | 8962.5  (6453.1, 12447.7) | 3311.7  (2071.0, 5295.9) | 5620.3  (3987.3, 7922.2) |
| Visit 2 - Day 29 | n | 23 | 23 | 23 | 23 |
|  | GMEU  (95% CI) | 43119.9  (30406.8, 61148.2) | 21098.4  (15562.1, 28604.2) | 31910.3  (22922.3, 44422.6) | 6271.8  (4719.4, 8334.8) |
|  | GMFR (95% CI) | 4.1 (2.9, 5.7) | 2.4 (1.7, 3.3) | 9.6 (5.9, 15.8) | 1.1 (1.0, 1.3) |
| Visit 4 - Day 181 | n | 22 | 23 | 22 | 23 |
|  | GMEU  (95% CI) | 26745.2  (16661.2, 42932.4) | 14458.7  (11538.7, 18117.6) | 23224.5  (15694.6, 34367.3) | 11989.3  (9148.8, 15711.5) |
|  | GMFR (95% CI) | 2.6 (1.4, 4.8) | 1.6 (1.2, 2.2) | 7.3 (3.8, 14.1) | 2.1 (1.5, 3.1) |

n: number of participants with a non-missing value at respective visit, GMEU: Geometric Mean ELISA Units, GMFR: Geometric Mean Fold Rise, CI: Confidence Interval

For each study vaccine, the GMEU and GMFR of anti-S IgG and 95% CI were calculated by transforming to the original scale of log 10-transformed mean and its two-sided 95% CI limits at each visit.

**Table S2: Summary of Proportion of Participants with Seroresponse for anti-S IgG against Prototype strain and Variants of Concerns - Per Protocol Population**

| **Timepoint** | **Statistic** | **ChAdOx1 nCoV-19 Prime Cohort** | | **BBV152 Prime Cohort** | |
| --- | --- | --- | --- | --- | --- |
|  |  | **SII-NVX-CoV2373 (N=92)** | **ChAdOx1 nCoV-19 (N=93)** | **SII-NVX-CoV2373 (N=91)** | **BBV152 (N=93)** |
| **Anti-S IgG – Wuhan** | | | | | |
| Visit 2 - Day 29 | N Evaluated | 92 | 93 | 91 | 93 |
|  | n (%) | 80 (86.96) | 35 (37.63) | 86 (94.51) | 19 (20.43) |
|  | 95% CI | (78.32, 93.07) | (27.79, 48.28) | (87.64, 98.19) | (12.77, 30.05) |
| Visit 3 - Day 91 | N Evaluated | 91 | 93 | 89 | 92 |
|  | n (%) | 48 (52.75) | 39 (41.94) | 65 (73.03) | 36 (39.13) |
|  | 95% CI | (42.00, 63.31) | (31.78, 52.62) | (62.58, 81.90) | (29.12, 49.86) |
| Visit 4 - Day 181 | N Evaluated | 90 | 93 | 88 | 92 |
|  | n (%) | 52 (57.78) | 28 (30.11) | 50 (56.82) | 36 (39.13) |
|  | 95% CI | (46.91, 68.12) | (21.03, 40.50) | (45.82, 67.34) | (29.12, 49.86) |
| **Anti-S IgG - Omicron BA.1** | | | | | |
| Visit 2 - Day 29 | N Evaluated | 23 | 23 | 23 | 23 |
|  | n (%) | 17 (73.91) | 10 (43.48) | 21 (91.30) | 1 (4.35) |
|  | 95% CI | (51.59, 89.77) | (23.19, 65.51) | (71.96, 98.93) | (0.11, 21.95) |
| Visit 4 - Day 181 | N Evaluated | 22 | 23 | 22 | 23 |
|  | n (%) | 11 (50.00) | 10 (43.48) | 17 (77.27) | 13 (56.52) |
|  | 95% CI | (28.22, 71.78) | (23.19, 65.51) | (54.63, 92.18) | (34.49, 76.81) |
| **Anti-S IgG - Omicron BA.5** | | | | | |
| Visit 2 - Day 29 | N Evaluated | 23 | 23 | 23 | 23 |
|  | n (%) | 20 (86.96) | 11 (47.83) | 22 (95.65) | 1 (4.35) |
|  | 95% CI | (66.41, 97.22) | (26.82, 69.41) | (78.05, 99.89) | (0.11, 21.95) |
| Visit 4 - Day 181 | N Evaluated | 22 | 23 | 22 | 23 |
|  | n (%) | 12 (54.55) | 9 (39.13) | 17 (77.27) | 13 (56.52) |
|  | 95% CI | (32.21, 75.61) | (19.71, 61.46) | (54.63, 92.18) | (34.49, 76.81) |

N Evaluated is the number of participants with a non-missing value at Baseline and respective post-vaccination visits.

n is the number of participants with Seroresponse (i.e. at least two-fold increase from baseline) at respective visits.

The 95% CIs for each vaccine group were calculated by using the Clopper-Pearson method.

**Table S3: Summary of neutralizing antibodies against Ancestral strain and Variants of Concerns - Per Protocol Population**

| **Timepoint** | **Statistic** | **ChAdOx1 nCoV-19 Prime Cohort** | | **BBV152 Prime Cohort** | |
| --- | --- | --- | --- | --- | --- |
|  |  | **SII-NVX-CoV2373 (N=92)** | **ChAdOx1 nCoV-19 (N=93)** | **SII-NVX-CoV2373 (N=91)** | **BBV152 (N=93)** |
| **Neutralizing antibodies – Ancestral strain** | | | | | |
| Visit 1 - Day 1 (Baseline) | n | 92 | 93 | 91 | 93 |
|  | GMT  (95% CI) | 772.6  (606.7, 984.0) | 726.5  (582.0, 906.8) | 274.8  (202.6, 372.8) | 203.1  (145.6, 283.4) |
| Visit 2 - Day 29 | n | 92 | 93 | 91 | 93 |
|  | GMT  (95% CI) | 3963.0  (3343.8, 4696.7) | 2031.9  (1690.6, 2442.1) | 2848.1  (2370.0, 3422.6) | 515.6  (395.4, 672.3) |
|  | GMFR (95% CI) | 5.1 (4.2, 6.2) | 2.8 (2.3, 3.4) | 10.4 (7.9, 13.5) | 2.5 (2.1, 3.1) |
| Visit 4 - Day 181 | n | 90 | 93 | 88 | 92 |
|  | GMT  (95% CI) | 2063.4  (1693.0, 2514.8) | 1612.7  (1337.0, 1945.2) | 1486.6  (1253.9, 1762.5) | 664.6  (505.9, 873.0) |
|  | GMFR (95% CI) | 2.7 (2.1, 3.4) | 2.2 (1.8, 2.7) | 5.3 (3.9, 7.2) | 3.3 (2.6, 4.2) |
| **Neutralizing antibodies - Omicron B.1.1.529 (BA.1)** | | | | | |
| Visit 1- Day 1 (Baseline) | n | 25 | 25 | 25 | 25 |
|  | GMT  (95% CI) | 47.2  (29.5, 75.5) | 67.7  (44.4, 103.4) | 51.3  (32.9, 80.0) | 49.9  (32.1, 77.7) |
| Visit 2 – Day 29 | n | 25 | 25 | 25 | 25 |
|  | GMT  (95% CI) | 164.5  (105.9, 255.6) | 86.9  (57.9, 130.7) | 194.3  (120.4, 313.4) | 41.1  (27.8, 60.8) |
|  | GMFR (95% CI) | 3.5 (2.3, 5.3) | 1.3 (1.0, 1.7) | 3.8 (2.3, 6.3) | 0.8 (0.6, 1.2) |
| Visit 4 Day 181 | n | 24 | 25 | 24 | 26 |
|  | GMT  (95% CI) | 246.8  (143.1, 425.4) | 164.5  (104.3, 259.5) | 169.5  (108.3, 265.3) | 119.3  (70.1, 203.2) |
|  | GMFR (95% CI) | 5.5 (2.9, 10.4) | 2.4 (1.6, 3.8) | 3.3 (1.7, 6.5) | 2.3 (1.4, 3.8) |

n: number of participants with a non-missing value at respective visit, GMT: Geometric Mean Titres, GMFR: Geometric Mean Fold Rise, CI: Confidence Interval

For each study vaccine, the GMT and GMFR of nAbs and 95% CI were calculated by transforming to the original scale of log 10-transformed mean and its two-sided 95% CI limits at each visit.

**Table S4: Summary of Proportion of Participants with Seroresponse for nAbs against Ancestral strain and Variants of Concerns - Per Protocol Population**

| **Timepoint** | **Statistic** | **ChAdOx1 nCoV-19 Prime Cohort** | | **BBV152 Prime Cohort** | |
| --- | --- | --- | --- | --- | --- |
|  |  | **SII-NVX-CoV2373 (N=92)** | **ChAdOx1 nCoV-19 (N=93)** | **SII-NVX-CoV2373 (N=91)** | **BBV152 (N=93)** |
| **Neutralizing Antibodies – Ancestral strain** | | | | | |
| Visit 2 - Day 29 | N Evaluated | 92 | 93 | 91 | 93 |
|  | n (%) | 87 (94.57) | 74 (79.57) | 90 (98.90) | 69 (74.19) |
|  | 95% CI | (87.77, 98.21) | (69.95, 87.23) | (94.03, 99.97) | (64.08, 82.71) |
| Visit 4 - Day 181 | N Evaluated | 90 | 93 | 88 | 92 |
|  | n (%) | 63 (70.00) | 67 (72.04) | 70 (79.55) | 66 (71.74) |
|  | 95% CI | (59.43, 79.21) | (61.78, 80.86) | (69.61, 87.40) | (61.39, 80.64) |
| **Neutralizing Antibodies - B.1.1.529 (BA.1)** | | | | | |
| Visit 2 - Day 29 | N Evaluated | 25 | 25 | 25 | 25 |
|  | n (%) | 21 (84.00) | 10 (40.00) | 18 (72.00) | 6 (24.00) |
|  | 95% CI | (63.92, 95.46) | (21.13, 61.33) | (50.61, 87.93) | (9.36, 45.13) |
| Visit 4 - Day 181 | N Evaluated | 24 | 25 | 24 | 25 |
|  | n (%) | 18 (75.00) | 18 (72.00) | 15 (62.50) | 14 (53.85) |
|  | 95% CI | (53.29, 90.23) | (50.61, 87.93) | (40.59, 81.20) | (33.37, 73.41) |

N Evaluated is the number of participants with a non-missing value at Baseline and respective post-vaccination visits.

n is the number of participants with Seroresponse (i.e. at least two-fold increase from baseline) at respective visits.

The 95% CIs for each vaccine group were calculated by using the Clopper-Pearson method.

**Table S5: Summary of hACE2 receptor inhibition antibody titers against Prototype strain and Variants of Concerns – Per Protocol Population**

| **Timepoint** | **Statistic** | **ChAdOx1 nCoV-19 Prime Cohort** | | **BBV152 Prime Cohort** | |
| --- | --- | --- | --- | --- | --- |
|  |  | **SII-NVX-CoV2373 (N=92)** | **ChAdOx1 nCoV-19 (N=93)** | **SII-NVX-CoV2373 (N=91)** | **BBV152 (N=93)** |
| **Wuhan** | | | | | |
| Visit 1- Day 1 (Baseline) | n | 23 | 23 | 23 | 23 |
|  | GMT (95% CI) | 108.3 (70.4, 166.5) | 81.9 (59.0, 113.7) | 23.3 (14.7, 36.8) | 42.6 (27.9, 65.1) |
| Visit 2 - Day 29 | n | 23 | 23 | 23 | 23 |
|  | GMT (95% CI) | 332.9 (242.8, 456.3) | 162.9 (125.9, 210.9) | 225.6 (155.3, 327.7) | 45.2 (29.8, 68.7) |
|  | GMFR (95% CI) | 3.1 (2.2, 4.3) | 2.0 (1.5, 2.6) | 9.7 (5.9, 15.9) | 1.1 (0.9, 1.2) |
| Visit 4 - Day 181 | n | 22 | 23 | 22 | 23 |
|  | GMT (95% CI) | 208.1 (126.3, 342.7) | 115.2 (90.0, 147.5) | 140.0 (98.4, 199.2) | 69.2 (46.2, 103.8) |
|  | GMFR (95% CI) | 2.0 (1.0, 3.9) | 1.4 (1.0, 2.0) | 6.1 (3.2, 11.6) | 1.6 (1.3, 2.1) |
| **Omicron BA.1** | | | | | |
| Visit 1- Day 1 (Baseline) | n | 23 | 23 | 23 | 23 |
|  | GMT (95% CI) | 42.0 (26.8, 65.8) | 43.7 (28.8, 66.3) | 15.3 (9.6, 24.3) | 20.1 (13.7, 29.5) |
| Visit 2 - Day 29 | n | 23 | 23 | 23 | 23 |
|  | GMT (95% CI) | 139.6 (95.8, 203.4) | 85.2 (62.2, 116.7) | 108.4 (70.8, 166.0) | 24.0 (17.8, 32.5) |
|  | GMFR (95% CI) | 3.3 (2.5, 4.5) | 2.0 (1.4, 2.7) | 7.1 (4.4, 11.4) | 1.2 (1.0, 1.5) |
| Visit 4 - Day 181 | n | 22 | 23 | 22 | 23 |
|  | GMT (95% CI) | 148.9 (89.4, 247.9) | 103.9 (73.5, 147.0) | 125.6 (80.9, 195.0) | 85.1 (62.2, 116.5) |
|  | GMFR (95% CI) | 3.7 (1.9, 7.1) | 2.4 (1.5, 3.8) | 8.3 (4.0, 17.3) | 4.2 (2.6, 7.0) |
| **Omicron BA.5** | | | | | |
| Visit 1- Day 1 (Baseline) | n | 23 | 23 | 23 | 23 |
|  | GMT (95% CI) | 61.6 (38.1, 99.5) | 58.2 (39.3, 86.0) | 19.2 (11.8, 31.3) | 35.1 (23.8, 51.8) |
| Visit 2 - Day 29 | n | 23 | 23 | 23 | 23 |
|  | GMT (95% CI) | 203.0 (139.2, 296.0) | 105.3 (79.4, 139.6) | 134.1 (85.6, 209.9) | 37.3 (26.2, 52.9) |
|  | GMFR (95% CI) | 3.3 (2.4, 4.5) | 1.8 (1.2, 2.7) | 7.0 (4.3, 11.3) | 1.1 (0.9, 1.3) |
| Visit 4 - Day 181 | n | 22 | 23 | 22 | 23 |
|  | GMT (95% CI) | 202.8 (128.6, 319.6) | 117.0 (90.5, 151.4) | 149.2 (94.8, 234.7) | 98.1 (76.1, 126.4) |
|  | GMFR (95% CI) | 3.4 (1.8, 6.3) | 2.0 (1.3, 3.1) | 8.0 (3.7, 17.2) | 2.8 (1.7, 4.6) |

n: number of participants with a non-missing value at respective visit. GMT: Geometric Mean Titre, GMFR: Geometric Mean Fold Rise

For each study vaccine, the GMT and GMFR of hACE2 receptor inhibition antibodies and 95% CI were calculated by transforming to the original scale of log 10-transformed mean and its two-sided 95% CI limits at each visit.

**Table S6: Summary of Proportion of Participants with Seroresponse for hACE2 receptor inhibition against Prototype strain and Variants for Concern - - Per Protocol Population**

| **Timepoint** | **Statistic** | **ChAdOx1 nCoV-19 Prime Cohort** | | **BBV152 Prime Cohort** | |
| --- | --- | --- | --- | --- | --- |
|  |  | **SII-NVX-CoV2373 (N=92)** | **ChAdOx1 nCoV-19 (N=93)** | **SII-NVX-CoV2373 (N=91)** | **BBV152 (N=93)** |
| **Wuhan** | | | | | |
| Visit-2 Day 29 | N Evaluated | 23 | 23 | 23 | 23 |
|  | n (%) | 16 (69.57) | 9 (39.13) | 23 (100.00) | 0 |
|  | 95% CI | (47.08, 86.79) | (19.71, 61.46) | (85.18, 100.00) | (0.00, 14.82) |
| Visit-4 Day 181 | N Evaluated | 22 | 23 | 22 | 23 |
|  | n (%) | 10 (45.45) | 6 (26.09) | 16 (72.73) | 8 (34.78) |
|  | 95% CI | (24.39, 67.79) | (10.23, 48.41) | (49.78, 89.27) | (16.38, 57.27) |
| **Omicron BA. 1** | | | | | |
| Visit-2 Day 29 | N Evaluated | 23 | 23 | 23 | 23 |
|  | n (%) | 18 (78.26) | 10 (43.48) | 21 (91.30) | 3 (13.04) |
|  | 95% CI | (56.30, 92.54) | (23.19, 65.51) | (71.96, 98.93) | (2.78, 33.59) |
| Visit-4 Day 181 | N Evaluated | 22 | 23 | 22 | 23 |
|  | n (%) | 14 (63.64) | 12 (52.17) | 17 (77.27) | 17 (73.91) |
|  | 95% CI | (40.66, 82.80) | (30.59, 73.18) | (54.63, 92.18) | (51.59, 89.77) |
| **Omicron BA. 5** | | | | | |
| Visit-2 Day 29 | N Evaluated | 23 | 23 | 23 | 23 |
|  | n (%) | 18 (78.26) | 9 (39.13) | 20 (86.96) | 1 (4.35) |
|  | 95% CI | (56.30, 92.54) | (19.71, 61.46) | (66.41, 97.22) | (0.11, 21.95) |
| Visit-4 Day 181 | N Evaluated | 22 | 23 | 22 | 23 |
|  | n (%) | 13 (59.09) | 12 (52.17) | 16 (72.73) | 15 (65.22) |
|  | 95% CI | (36.35, 79.29) | (30.59, 73.18) | (49.78, 89.27) | (42.73, 83.62) |

N Evaluated is the number of participants with a non-missing value at Baseline and respective post-vaccination visits.

n is the number of participants with Seroresponse (i.e. at least two-fold increase from baseline) at respective visits.

The 95% CIs for each vaccine group were calculated by using the Clopper-Pearson method.

**Table S7: Summary of Actual Value and Change from Baseline in Cell-Mediated Immune Response (IFN-gamma: SFUs /million cells)**

**Per Protocol Population**

| **Visit** | | **Actual Value** | | | | | | **Change from Baseline [1]** | | | | | |
| --- | --- | --- | --- | --- | --- | --- | --- | --- | --- | --- | --- | --- | --- |
|  |  | **n** | **Mean** | **SD** | **Median** | **Min** | **Max** | **n** | **Mean** | **SD** | **Median** | **Min** | **Max** |
| **Visit 1 - Day 1 (Baseline)** | | | | | | | | | | | | | |
| ChAdOx1 nCoV-19 Prime Cohort | SII-NVX-CoV2373 (N=92) | 9 | 51.7 | 24.28 | 60.0 | 17 | 80 |  |  |  |  |  |  |
|  | ChAdOx1 nCoV-19 (N=93) | 8 | 99.4 | 147.16 | 30.8 | 5 | 418 |  |  |  |  |  |  |
| BBV152  Prime Cohort | SII-NVX-CoV2373 (N=91) | 8 | 127.5 | 111.14 | 103.3 | 8 | 357 |  |  |  |  |  |  |
|  | BBV152 (N=93) | 7 | 57.7 | 63.24 | 23.3 | 3 | 173 |  |  |  |  |  |  |
| **Visit 2 - Day 29** | | | | | | | | | | | | | |
| ChAdOx1 nCoV-19 Prime Cohort | SII-NVX-CoV2373 (N=92) | 10 | 95.7 | 112.78 | 33.3 | 2 | 302 | 9 | 54.4 | 108.29 | 20.0 | -68 | 228 |
|  | ChAdOx1 nCoV-19 (N=93) | 8 | 68.4 | 49.44 | 56.5 | 2 | 158 | 8 | -31.0 | 130.61 | 22.9 | -260 | 72 |
| BBV152  Prime Cohort | SII-NVX-CoV2373 (N=91) | 9 | 259.1 | 394.27 | 123.3 | 15 | 1262 | 8 | 155.0 | 346.44 | 19.2 | -175 | 905 |
|  | BBV152 (N=93) | 8 | 102.1 | 59.96 | 97.5 | 25 | 185 | 6 | 61.8 | 31.97 | 70.8 | 17 | 100 |
| **Visit 4 - Day 181** | | | | | | | | | | | | | |
| ChAdOx1 nCoV-19 Prime Cohort | SII-NVX-CoV2373 (N=92) | 10 | 165.9 | 151.72 | 106.2 | 38 | 466 | 9 | 124.5 | 171.80 | 51.7 | -32 | 448 |
|  | ChAdOx1 nCoV-19 (N=93) | 8 | 242.9 | 144.94 | 247.5 | 67 | 433 | 8 | 143.5 | 234.85 | 192.7 | -352 | 428 |
| BBV152  Prime Cohort | SII-NVX-CoV2373 (N=91) | 9 | 117.1 | 109.67 | 84.9 | 24 | 390 | 8 | -6.4 | 128.91 | 3.5 | -266 | 192 |
|  | BBV152 (N=93) | 9 | 146.2 | 49.20 | 155.9 | 82 | 254 | 7 | 100.7 | 82.01 | 85.2 | -12 | 230 |

[1] Change from baseline: post-baseline value — baseline value.
