# Supplementary material for: A Phase 3, randomized, non-inferiority study of a heterologous booster dose of SARS CoV-2 recombinant spike protein vaccine in adults
Source: Sci Rep. 2023 Oct 3;13:16579. doi: 10.1038/s41598-023-43578-w (PMC10547846; doi:10.1038/s41598-023-43578-w)
Supplement: Supplementary file 2 — Supplementary Information 2. [file 41598_2023_43578_MOESM2_ESM.pdf]

CLINICAL TRIAL PROTOCOL

A PHASE 3, OBSERVER-BLIND, RANDOMIZED, CONTROLLED STUDY TO EVALUATE THE SAFETY AND IMMUNOGENICITY OF A BOOSTER DOSE OF COVOVAX IN INDIAN ADULTS WHO HAVE RECEIVED PRIMARY VACCINATION AGAINST COVID-19

Protocol number:	COVOVAX-Booster
Version:	4.0
Date:	21 Apr 2022
Amendment:	3
Investigational Products:	Test product: COVOVAX [SARS-CoV-2 recombinant spike protein nanoparticle vaccine (SARS-CoV-2 rS) with Matrix-M1™ adjuvant]<br><br>Reference (control) product:<br>• Covishield for Covishield prime cohort<br>• Covaxin for Covaxin prime cohort

[redacted]

CONFIDENTIAL

This document is confidential and is to be distributed for review only to investigators, potential investigators, consultants, study staff, and applicable Independent Ethics Committees or Institutional Review Boards. The contents of this document shall not be disclosed to others without written authorization from SIIPL, unless it is necessary to obtain informed consent from potential study participants.

TABLE OF CONTENTS

LIST OF ABBREVIATIONS 4

1. GENERAL INFORMATION 17

2. INTRODUCTION & BACKGROUND INFORMATION 18

2.1 INTRODUCTION 18

2.2 SARS-COV-2 RECOMBINANT SPIKE PROTEIN NANOPARTICLE VACCINE (SARS-COV-2 RS) WITH MATRIX-M1™ ADJUVANT 19

2.3 MATRIX-M ADJUVANT 20

2.4 COVOVAX: 21

2.5 RATIONALE FOR STUDY DESIGN 29

3. OBJECTIVES AND ENDPOINTS 31

3.1 CO-PRIMARY OBJECTIVE(S) 31

3.2 SECONDARY OBJECTIVES AND ESTIMANDS 32

3.3 EXPLORATORY OBJECTIVES 35

4. STUDY DESIGN 36

5. STUDY POPULATION 38

5.1 INCLUSION CRITERIA 38

5.2 EXCLUSION CRITERIA 38

6. TREATMENT OF STUDY PARTICIPANTS 40

6.1 DESCRIPTION OF STUDY VACCINES 40

6.2 PRECAUTIONS TO BE OBSERVED IN ADMINISTRATING STUDY VACCINES 42

6.3 PREPARATION AND ADMINISTRATION OF THE STUDY VACCINE 42

6.4 VACCINE SUPPLY, LABELLING, STORAGE, ACCOUNTABILITY AND DISPOSAL 43

7. STUDY PROCEDURES 44

7.1 GENERAL CONSIDERATIONS 44

7.2 STUDY VISITS 45

7.3 TESTING FOR COVID-19 DURING THE STUDY PERIOD: 49

7.4 PARTICIPANT DISCONTINUATION 49

7.5 MANAGEMENT OF PREGNANCY DURING STUDY 51

7.6 PRIOR AND CONCOMITANT THERAPY 51

8. ASSESSMENTS OF IMMUNOGENICITY AND DISEASE INCIDENCE 52

8.1 IMMUNOGENICITY: 52

8.2 INCIDENCE OF COVID-19: 52

8.3 METHODS FOR PROCESSING, LABEL AND STORAGE OF BLOOD SAMPLES 52

9. ASSESSMENT OF SAFETY 53

9.1 SAFETY MONITORING 53

9.2 PROTOCOL SAFETY REVIEW TEAM (PSRT) 53

9.3 ADVERSE EVENT (AE) 53

9.4 SERIOUS ADVERSE EVENT (SAE) 54

9.5 REPORTING PERIOD AND PARAMETER 54

9.6 ADVERSE EVENTS OF SPECIAL INTEREST (AESI) 55

9.7 SEVERITY OF ADVERSE EVENTS 55
9.8 CAUSALITY OF ADVERSE EVENTS 56
9.9 FOLLOW-UP OF ADVERSE EVENTS 57
9.10 GENERAL GUIDANCE ON REPORTING ADVERSE EVENTS 57
9.11 REPORTING OF SAE 58
9.12 TREATMENT OF AE AND SAEs 60

10. STATISTICAL CONSIDERATIONS 60

10.1 OVERVIEW AND GENERAL CONSIDERATIONS 60
10.2 RANDOMIZATION 61
10.3 MULTIPLICITY 61
10.4 SAMPLE SIZE AND POWER 61
10.5 ANALYSIS POPULATIONS 63
10.6 ANALYSIS PLAN 64

11. QUALITY CONTROL AND QUALITY ASSURANCE 83

11.1 PRE-STUDY DOCUMENTATION 83
11.2 MONITORING 83
11.3 DATA MANAGEMENT AND PROCESSING 84
11.4 STUDY AND SITE CLOSURE 85
11.5 AUDITS AND INSPECTIONS 86

12. REGULATORY AND ETHICAL REQUIREMENTS 87

12.1 ETHICS COMMITTEE REVIEW AND COMMUNICATION 87
12.2 PROTOCOL AMENDMENTS 87
12.3 PARTICIPANT INFORMATION AND INFORMED CONSENT 88
12.4 PARTICIPANT CONFIDENTIALITY 88
12.5 ETHICAL CONDUCT OF THE STUDY 89

13. DATA HANDLING AND RECORD KEEPING 89

14. INSURANCE AND COMPENSATION OF STUDY PARTICIPANTS 90

15. PUBLICATION POLICY & CONFIDENTIALITY 90

16. REFERENCES 92

APPENDIX:

I. Listings of AESI including PIMMCs and AESI related to COVID-19
II. COVID-19 disease symptoms and severity definition
III. Division of AIDS (DAIDS) Table for Grading the Severity of Adult and Pediatric Adverse Events, corrected version 2.1, July 2017
IV. Declaration of Helsinki
V. Sponsor’s Signature Page
VI. Investigator’s statement of compliance
VII. Study Sites and Principal Investigator Information

LIST OF ABBREVIATIONS

ACE2	Angiotensin-Converting Enzyme 2
ADE	Antibody Dependent Enhancement
AE	Adverse event
AESI	Adverse event of special interest
CI	Confidence interval
CLIA	Chemiluminescence Immunoassay
CMI	Cell Mediated Immunity
CoV	Coronavirus
COVID-19	Coronavirus Disease 2019
CRF	Case report form
CRO	Contract research organization
CTRI	Clinical trials registry of India
DS	Drug Substance
DP	Drug Product
DCGI	Drugs controller general of India
E	Envelope protein
ELISpot	Enzyme- linked Immunospot
GCP	Good Clinical Practices
GCLP	Good Clinical Laboratory Practices
GMTs	Geometric mean titers
ICF	Informed consent form
ICMR	Indian Council of Medical Research
ICU	Intensive Care Unit
IEC	Institutional Ethics Committee
IFN-γ	Interferon-gamma
IM	Intramuscular
M	Membrane protein
MedDRA	Medical Dictionary for Regulatory Activities
N	Nucleocapsid protein
nAb	Neutralizing Antibody against SARS CoV-2
NARI	National AIDS Research Institute
NHPs	Non-Human Primates
RT-PCR	Reverse transcription Polymerase Chain Reaction
PI	Principal Investigator
PIMMCs	Potential immune mediated medical conditions

PSRT	Protocol Safety Review Team
PT	Preferred term
RBD	Receptor Binding Domain
rS	Recombinant spike protein
S	Spike glycoprotein
SAE	Serious adverse event
SARS-CoV	Severe Acute Respiratory Syndrome-Coronavirus
SIIPL	Serum Institute of India Private Limited.
SOC	System Organ Class
SOP	Standard Operating Procedure
UPT	Urine Pregnancy Test
WHO	World Health Organization

PROTOCOL SUMMARY

**Title**	A phase 3, observer-blind, randomized, controlled study to evaluate the safety and immunogenicity of a booster dose of COVOVAX in Indian adults who have received primary vaccination against COVID-19
**Protocol number**	COVOVAX-Booster
**Phase**	3
**Study rationale**	The COVID-19 epidemic has caused major disruption to healthcare systems with significant socioeconomic impacts.<br>Novavax has developed SARS-CoV-2 rS Nanoparticle Vaccine with Matrix adjuvant (Nuvaxovid). After technology transfer, the same vaccine is also manufactured by Serum Institute of India Pvt Ltd (SIIPL) as COVOVAX. This vaccine has been already administered in more than 30000 adults across ongoing Phase 1/2, Phase 2, Phase 2/3 and Phase 3 studies in Australia, South Africa, UK, USA, Mexico and India without any serious safety concerns. Efficacy of about 90% has been demonstrated in both UK and USA Phase 3 clinical trials against any severity symptomatic COVID-19 and 100% against severe COVID-19, hospitalization and deaths due to COVID-19. Based on these data, COVOVAX as well as Nuvaxovid were granted Emergency Use Listing (EUL) by the World Health Organization (WHO). Nuvaxovid has been granted conditional marketing authorization by EMA and COVOVAX has also received emergency use authorization in India, Indonesia and Philippines as of 03 January 2022.<br>It has become evident that the protection offered against COVID-19 wanes after a two-dose schedule of COVID-19 vaccines, potential role of third or booster dose against COVID-19 is being explored by several countries. Many vaccines have already received approval to be used as third dose or booster dose and currently more than 100 countries have started administering booster doses. Nuvaxovid has already been used for both homologous as well as heterologous boosting in clinical trials and has shown that it induces robust booster response in both the regimens.<br>Considering this background, this study will evaluate the boosting response to COVOVAX in those individuals $\geq$ 18 years of age who have received 2

<table>
<tr><td></td><td>doses of COVID-19 vaccines at least 6 months (180 days) ago.</td></tr>
<tr><td>Population</td><td>372 adults ≥ 18 years of age</td></tr>
<tr><td>Participation Duration</td><td>Approximately 6 months</td></tr>
<tr><td>Description of study vaccine (s)</td><td>
<u>TEST VACCINE</u>
COVOVAX [SARS-CoV-2 recombinant spike protein nanoparticle vaccine (SARS-CoV-2 rS) with Matrix-M1™ adjuvant] is available as a ready to use liquid formulation in a 10-dose vial (5.0 mL). Each dose of 0.5 mL contains 5 µg antigen and 50 µg Matrix-M1 adjuvant. The other ingredients are 25 mM phosphate buffer (pH 7.2), 300 mM sodium chloride, and 0.01% (v/v) polysorbate 80.
Manufacturer: Serum Institute of India Pvt. Ltd.
<u>COMPARATOR VACCINE (CONTROL VACCINE)</u>
<u>Covishield for Covishield Prime Cohort</u>
COVISHIELD™ [ChAdOx1 nCoV-19 Corona Virus Vaccine (Recombinant)]
One dose (0.5 ml) contains:
ChAdOx1 nCoV- 19 Corona Virus Vaccine (Recombinant) 5 × 1010 virus particles (vp)
*Recombinant, replication-deficient chimpanzee adenovirus vector encoding the SARS-CoV-2 Spike (S) glycoprotein. Produced in genetically modified human embryonic kidney (HEK) 293 cells.
Manufacturer: Serum Institute of India Pvt. Ltd.

<u>Covaxin for Covaxin Prime Cohort</u>
COVAXIN® (Whole Virion Inactivated Coronavirus (SARS-CoV-2) Vaccine) is a white to off white, opalescent suspension free from extraneous particles containing 6 µg of Whole Virion, Inactivated (SARS-CoV-2) Antigen (strain NIV-2020-770).

Each dose of 0.5mL contains:
<table>
<tr><th>Ingredient</th><th>Quantity</th></tr>
<tr><td>Whole Virion, Inactivated Coronavirus (SARS-CoV-2) Antigen (Strain: NIV-2020-770)</td><td>6 µg</td></tr>
<tr><td>Aluminium Hydroxide Gel equivalent to Al+++</td><td>0.25 mg</td></tr>
<tr><td>TLR7/8 Agonist</td><td>15 µg</td></tr>
<tr><td>2-Phenoxyethanol</td><td>2.5 mg</td></tr>
<tr><td>Phosphate Buffered Saline</td><td>q.s. to 0.5 mL</td></tr>
</table>
Manufacturer: Bharat Biotech International Limited
</td></tr>
</table>

	The study vaccines (COVOVAX / Covishield / Covaxin) will be administered intramuscularly (IM) as a single dose of 0.5 ml intramuscularly in the deltoid muscle.	
**Objectives and endpoints:**	**Objectives**	**Endpoints**
	**Co-primary**	
	To compare immunogenicity of the COVOVAX booster dose in comparison with the Control vaccine by anti-S IgG for COVISHIELD Prime, and COVAXIN Prime cohorts, separately	• Geometric Mean Titers (GMTs) ratio (GMR) of anti-S IgG at 28 days after vaccination between vaccines (COVOVAX booster/ control)<br>• Proportion of participants achieving ≥ 2-fold increase in anti-S IgG at 28 days after vaccination between vaccines (COVOVAX booster/ control)
	To compare immunogenicity of the COVOVAX booster dose in comparison with the Control vaccine by neutralizing antibodies (nAbs) for COVISHIELD Prime, and COVAXIN Prime cohorts, separately	• Geometric Mean Titers (GMTs) ratio (GMR) of nAbs at 28 days after vaccination between vaccines (COVOVAX booster/ control)<br>• Proportion of participants achieving ≥ 2-fold increase in nAbs at 28 days after vaccination between vaccines (COVOVAX booster/ control)
	**Secondary**	
	To assess the tolerability and reactogenicity profile of the COVOVAX in comparison with the Control vaccine for COVISHIELD Prime, and COVAXIN Prime cohorts, separately	a. Occurrence of solicited local and systemic adverse events (AEs) for 7 days post-vaccination<br>b. Occurrence of unsolicited AEs for 28 days post-vaccination<br>c. Occurrence of SAEs, and adverse events of special

		interest (AESI) throughout the study duration following vaccination
	To assess immunogenicity of the COVOVAX booster dose in comparison with the Control vaccine by anti-S IgG and neutralizing antibody assays for COVISHIELD Prime, and COVAXIN Prime cohorts, separately	a. GMTs and GMFR of anti-S IgG antibodies at 28, 90, and 180 days post-vaccination.<br>b. GMTs and GMFR of nAbs at 28 and, 180 days post-vaccination.
	To assess immune response of COVOVAX booster dose between COVISHIELD Prime and COVAXIN Prime cohorts by anti-S IgG and neutralizing antibody assays	a. GMTs of anti-S IgG antibodies at 28, 90 and 180 days after vaccination<br>b. GMTs of neutralizing antibodies at 28 and 180 days after vaccination
	To assess immune response of Covishield booster dose in COVISHIELD Prime and Covaxin booster dose in COVAXIN Prime cohorts by anti-S IgG and neutralizing antibody assays	a. GMTs of anti-S IgG antibodies at 28, 90 and 180 days after vaccination<br>b. GMTs of nAbs at 28 and 180 days after vaccination.
	**Exploratory**	
	To assess cell mediated immune response of the COVOVAX in comparison with the Control vaccine for COVISHIELD Prime, and COVAXIN Prime cohorts, separately	The mean of change from Baseline (Day 1) in the cell-mediated immune responses as measured by enzyme-linked immune absorbent spot (ELISpot) at 28 days and 180 days post-vaccination.
	To assess immune response by neutralizing antibodies against variants of concern for	GMTs and GMFR of nAbs against variants of concern at 28 and 180 days post-vaccination.

<table>
<tr><td></td><td>COVISHIELD Prime, and COVAXIN Prime cohorts, separately</td><td></td></tr>
<tr><td>Study Design:</td><td colspan="2">
This is a Phase 3, observer-blinded, randomised, active controlled study in adults ≥ 18 years of age in India who have already received primary vaccination against COVID-19 at least 6 months ago (6 months / 180 days from the second dose), to evaluate the immunogenicity and safety of the COVOVAX booster dose in comparison with the control vaccine.
A total of 372 eligible participants of ≥ 18 years of age who have completed primary 2 dose schedule of COVID-19 vaccination at least 6 months ago will be enrolled in this study in 2 cohorts of 186 participants each with 1:1 allocation to COVOVAX or control vaccine. Two cohorts of 186 participants each will be as below:

COVISHIELD Prime cohort: Primary vaccination with two doses of COVISHIELD
COVAXIN Prime cohort: Primary vaccination with two doses of COVAXIN

All eligible participants (n=372) will receive a single dose of 0.5 mL of either COVOVAX or Control vaccine on Day 1 as per randomization. Post vaccination site visits are planned on Day 29, Day 91, and Day 181.
Treatment allocation:
<table>
<tr><td rowspan="2">COVISHIELD Prime cohort</td><td>COVOVAX</td><td>93</td></tr>
<tr><td>Covishield™</td><td>93</td></tr>
<tr><td rowspan="2">COVAXIN Prime cohort</td><td>COVOVAX</td><td>93</td></tr>
<tr><td>Covaxin™</td><td>93</td></tr>
</table>
Approximately 10 ml blood sample will be collected at baseline (Day 1), Day 29, Day 91 and Day 181. Additionally, up to 20 ml blood sample may be collected from subset of 36 participants (18 from each of the two prime cohorts) for assessment of cell mediated immune (CMI) responses at baseline, Day 29 and Day 181.
</td></tr>
<tr><td>Inclusion and Exclusion Criteria</td><td colspan="2">
Inclusion criteria:
Eligible participants must meet all of the below criteria at the time of enrolment:
Adults aged ≥ 18 years of either sex
</td></tr>
</table>

	2. Written informed consent by participants<br>3. Those who have completed primary COVID-19 vaccination schedule with either Covishield or Covaxin (2 doses) at least 6 months ago (6 months / 180 days from the second dose)<br>4. The participant is resident of the study area and is willing to comply with study protocol requirements, including availability for all scheduled visits of the study<br>5. Healthy / clinically stable condition, as determined by medical history and physical examination<br>6. Sexually active female participants of childbearing potential* must have practiced adequate contraception for 28 days prior to study vaccine administration and agree to continue adequate contraception until completion of 28 days after vaccination<br>* Females can be considered not of childbearing potential only if they have undergone bilateral tubal ligation or occlusion, or hysterectomy, or bilateral oophorectomy, or are post- menopausal (defined as continuous amenorrhea for 12 months)<br>7. Female participants of childbearing potential must have a negative urine pregnancy test within 24 hours prior to study vaccine administration<br>**Exclusion criteria:**<br>Participants meeting any of the below criteria at the time of enrolment will be ineligible to participate in the trial:<br>1. Acute illness including COVID-19 with or without fever at the time of screening<br>2. History of laboratory confirmed (by RT-PCR, rapid antigen test or serology to SARS-CoV-2) COVID-19<br>3. History of allergic reactions after previous vaccinations including primary vaccination for COVID-19<br>4. Hypersensitivity to any component of study vaccines<br>5. Any confirmed or suspected condition with impaired/altered function of immune system (e.g. immunodeficient or autoimmune conditions)<br>**Note:** Stable endocrine disorders that have a confirmed autoimmune

etiology (e.g., thyroid, pancreatic) are allowed.

6. Have any bleeding disorder which is considered as a contraindication to intramuscular injection or blood draw
7. Suspected or known current alcohol or drug dependence
8. Chronic administration (defined as more than 14 continuous days) of immunosuppressant or other immune-modifying drugs within three months prior to the study vaccination or planned use throughout the study period (For corticosteroids, this means prednisone, or equivalent, $\geq$ 0.5 mg/kg per day. Inhaled, intranasal and topical steroids are allowed)
9. Administration of blood, blood products and/or plasma derivatives or any parenteral immunoglobulin preparation in the past 3 months or planned use throughout the study period
10. Continuous use of anticoagulants, such as coumarins and related anticoagulants (i.e. warfarin) or novel oral anticoagulants (i.e. apixaban, rivaroxaban, dabigatran and edoxaban etc)
11. Administration of any vaccine within 28 days prior to enrolment in the study or planned administration of any vaccine for until 28 days after study vaccination
12. Prior receipt of a booster dose of COVID-19 vaccine
13. Planning to receive a booster dose of COVID-19 vaccine during the course of the study
14. Use of any investigational or non-registered drug or vaccine within 30 days prior to the administration of study vaccines or planned during the study
15. Pregnant or breast-feeding
16. Acute or chronic, clinically significant pulmonary, cardiovascular, endocrine, metabolic, gastrointestinal, neurological, hepatic, renal functional abnormality or any other systemic disorder, that are assessed by the investigator (based on medical history or physical examination) as being clinically unstable within the prior 4 weeks (mild or moderate well-controlled comorbidities are allowed)

	17. History of chronic neurological disorders such as multiple sclerosis, dementia, transient ischemic attacks, Parkinson's disease, degenerative neurological conditions, neuropathy, and epilepsy or a history of stroke or previous neurological disorder within 12 months with residual symptoms<br>**<u>Note:</u>** Participants with a history of migraine or chronic headaches or nerve root compression that have been stable on treatment for the last 4 weeks not to be excluded<br>18. Any other condition that in the opinion of the investigator would jeopardize the safety or rights of the participant in the study or make it unlikely that the participant could complete the protocol
**Study Conduct:**	The study will be initiated after permissions from the Drugs Controller General of India (DCGI) and Institutional Ethics Committee (IEC) of respective sites are obtained and registration of the study on Clinical Trial Registry of India (CTRI) is completed. The participants will be screened for eligibility after written informed consent is obtained. In female participants of childbearing age, urine pregnancy test (UPT) will be done on Day 1 before randomizing the study participant. In addition, nasopharyngeal / nasal and/or throat swab will be collected for RT-PCR test for detection of SARS-CoV-2 infection.<br>A total of 372 eligible participants (186 participants from each of 2 cohorts) will be randomized as mentioned above to receive study vaccines. The study vaccines will be injected intramuscularly in the deltoid as a single dose of 0.5 mL on Day 1. The participants will be observed closely for at least 30 minutes following vaccination.<br>Participants will return to the clinical study site for follow up on Day 29 (+7 days), Day 91 (+7 days), and Day 181 (+14 days).<br>Approximately up to 10 ml blood sample will be collected at baseline (Day 1), Day 29, Day 91 and Day 181 post-vaccination for immunogenicity assessments. Additionally, up to 20 ml blood sample may be collected from subset of 36 participants (18 from each of the two prime cohorts) for assessment of cell mediated immune (CMI) responses at baseline, Day 29

	and Day 181.<br>Physical examination (PE) and vital sign evaluations will be performed and medical history and prior/concomitant medications will be captured on Day 1 (Full PE), Day 29 (+7 days), Day 91 (+7 days) and Day 181 (+14 days) (Targeted PE for post vaccination visits). Vital sign measurement after 30 minutes (+15 minutes) post-vaccination will also be done.<br><br>**Adverse Events (AE):**<br>1. The solicited local AEs to be collected include pain, tenderness, erythema, swelling and induration. The solicited systemic AEs to be collected include fever, headache, fatigue, malaise, arthralgia, myalgia, nausea and vomiting. Solicited local and systemic AEs will be actively collected for 7 days after vaccination using participant diary cards.<br>2. Unsolicited adverse events will be collected for 28 days post-vaccination.<br>3. SAEs, and AESIs will be collected throughout the study participation after vaccination.<br>**Testing for COVID-19 during the study period:** Participants will be tested for COVID-19 if they present with symptoms of suspected COVID-19 disease [Appendix II, Table 1] or history of contact with a confirmed COVID-19 positive case.<br>Severe COVID-19 disease will be defined as per criteria described in Appendix II, Table 2. Detailed clinical parameters will be collected from medical records.<br>The recruitment for both the cohorts will be started simultaneously. When the recruitment for a cohort is completed, the 28 days safety and immunogenicity (anti-S IgG) data may be analyzed separately and submitted to regulatory authorities, as required. This interim analysis for each cohort will not have any impact on outcomes of the study as randomization for each cohort will be separate.
**Safety monitoring**	Safety will be monitored during the study by on-site clinical staff and

	routinely by the Protocol Safety Review Team (PSRT), an internal group of physicians which includes SIIPL Medical Officers, a biostatistician and designated pharmacovigilance medical officer from the CRO. The PSRT may seek independent expert medical opinion as dictated by the occurrence of certain events. There will be periodic reviews of accruing safety data by the PSRT.
**Statistical considerations**	It is planned to randomize 372 participants with 186 in each of 2 cohorts who have received primary vaccination with either COVISHIELD OR COVAXIN. Assuming that the proportion of non-evaluable participants ≤ 15% (which leads to a sample size of 158 evaluable participants for each cohort), group sample sizes of 93 each in the tested vaccine group and the active control group achieve at least 80% power to detect non-inferiority using a one-sided, two-sample t-test. The margin of non-inferiority is -0.33. The true ratio of the means at which the power is evaluated is 1.00. The one-sided significance level (alpha) of the test is 0.025. The coefficients of variation of both groups are assumed to be 1.1. Non-inferiority of COVOVAX booster over an active control vaccine group for each cohort will be concluded separately if the lower limit of the two-sided 95% CI for the GMT ratio for both anti-S IgG and neutralizing antibodies between COVOVAX and the active control vaccine at Day 29 is > 0.67. The same sample size to serve the statistical test for a non-inferiority difference in proportion of participants achieving ≥ 2-fold increase in Anti-S and nAbs between COVOVAX and the active control vaccine at Day 29 will achieve 82% power, using a one-sided, two-sample z-test at alpha level of 0.025. Proportion of participants achieving ≥ 2-fold increase in both Anti-S and nAbs for COVISHILED/ COVAXIN arm is assumed 95% and the margin of non-inferiority is -10%. The true difference of the proportion at which the power is evaluated is 0. Sample size calculations were performed using a non-inferiority test for the ratio of two means and difference in two proportions in PASS 15.0.7 Version software.<br><br>To assess the co-primary objectives, the non-inferiority hypotheses will be tested on the GMR between COVOVAX and COVISHIELD or COVOVAX

	and COVAXIN for anti-S IgG and nAbs at 28 days (Day 29) post study vaccination using an ANCOVA model for each cohort. The lower limit of the 95% CI will be compared with the non-inferiority margin 0.67 and COVOVAX will be declared non-inferior to the active Control vaccine, COVISHIELD or COVAXIN in each cohort if it is greater than 0.67.<br><br>The non-inferiority hypothesis of difference in proportions of participants with at least 2-fold increase in anti-S IgG and nAbs at Day 29 between COVOVAX and COVISHIELD or COVAXIN will be examined using the Miettinen and Nurminen method for 95% CI of the difference. If the lower limit is greater than the margin, -10%, then the non-inferiority of difference in proportions of participants with at least 2-fold increase in anti-S IgG and nAbs will be declared.<br><br>Detailed statistical analytical methods will be provided in statistical analysis plan (SAP) which will be prepared and approved before database lock.<br>An interim analysis is planned after completion of 28 days follow up after the vaccination of all study participants from each cohort separately or together depending on timelines and will include 28 days safety and immunogenicity data after vaccination (Day 29).

1. GENERAL INFORMATION

[redacted]	[redacted]
[redacted]	[redacted]
[redacted]	
[redacted]	
[redacted]	
[redacted]	

2. INTRODUCTION & BACKGROUND INFORMATION

2.1 INTRODUCTION

In December 2019, a cluster of a novel coronavirus, known as 2019-nCoV cases were identified in Wuhan, China.[1] The virus (subsequently named as SARS-CoV-2) is similar to the severe acute respiratory syndrome (SARS-CoV), and the Middle East respiratory syndrome (MERS-CoV) viruses.[2] Coronavirus disease 2019 (COVID-19) is the infectious disease caused by SARS-CoV-2.

As of 29 December 2021, there have been more than 281 million reported cases and more than 5.4 million deaths worldwide.[3] Importantly, as of 31 December 2021, India reported total confirmed cases of more than 34.8 million with more than 0.48 million fatalities as per data of Ministry of Health, Government of India.[4]

Coronaviruses (CoVs) are spherical, enveloped, large positive-sense single-stranded RNA genomes. One-fourth of their genome is responsible for coding structural proteins, such as the spike (S) glycoprotein, envelope (E), membrane (M) and nucleocapsid (N) proteins. E, M, and N are mainly responsible for virion assembly whilst the S protein is involved in receptor binding, mediating virus entry into host cells during CoVs infection via different receptors.[5] SARS-CoV-2 belongs to the phylogenetic lineage B of the genus Betacoronavirus and it recognises the angiotensin-converting enzyme 2 (ACE2) as the entry receptor.[6] It is the seventh CoV known to cause human infections and the third known to cause severe disease after SARS-CoV and MERS-CoV.

The spike (S) protein is a type I, trimeric, transmembrane glycoprotein located at the surface of the viral envelope of CoVs, which can be divided into two functional subunits: the N-terminal S1 and the C-terminal S2. S1 and S2 are responsible for cellular receptor binding via the receptor binding domain (RBD) and fusion of virus and cell membranes respectively, thereby mediating the entry of SARS-CoV-2 into target cells.[5] The roles of S protein in receptor binding and membrane fusion make it an ideal target for vaccine and antiviral development, as it is the main target for neutralising antibodies.

Although individuals of any age can acquire SARS-CoV-2, certain individuals are at a higher risk of infection with SARS-CoV-2. The high-risk group includes the health care workers

(physicians and paramedical staff) working amid COVID-19 patients and all other people including household contacts of COVID-19 confirmed patients or people currently residing or working in COVID-19 hotspots/outbreak areas where there is a high risk of transmission of SARS-CoV-2 infections. The SARS-CoV-2 infections tend to be severe in population with co-morbidities or elderly population aged ≥ 60 years and therefore such subjects living or currently working in COVID-19 affected areas, are also considered high-risk population.

The immunity achieved through natural infection has been shown to last for many months[6], long term immunogenicity data is not yet available for many vaccines. The available data shows that the durability of immune response may be affected by 2 factors; the waning of vaccine antibody titres over time, and the emergence of novel variants of the SARS-CoV-2 virus with substantive mutations to the spike protein which may need higher titres to neutralize these variants.[7] This is likely to necessitate booster doses of COVID-19 vaccines, and potentially periodic boosters. Boosters will either increase personal protection via generating higher antibody levels against the original pandemic variant regardless of circulating variant; or in due course by generating immunity specifically against novel variants of the virus.[8]

2.2 SARS-COV-2 RECOMBINANT SPIKE PROTEIN NANOPARTICLE VACCINE (SARS-COV-2 rS) WITH MATRIX-M1™ ADJUVANT

Novavax, Inc. has developed a recombinant vaccine adjuvanted with the saponin-based Matrix-M1™ (previously referred to as Matrix-M) for the prevention of disease caused by the SARS-CoV-2 virus. SARS-CoV-2 recombinant spike (rS) protein nanoparticle vaccine (SARS-CoV-2 rS) is constructed from the full length wild-type SARS-CoV-2 S glycoprotein (GP) based upon the GenBank gene sequence MN908947, nucleotides 21563-25384. The S protein is a type 1 trimeric glycoprotein of 1,273 amino acids that is produced as an inactive S0 precursor. The S-gene was codon optimized for expression in Spodoptera frugiperda (Sf9) insect cells. The SARS-CoV-2 rS nanoparticle vaccine is intended for administration with Matrix-M1™ adjuvant, which is a saponin-based adjuvant that has been shown to enhance the immunogenicity of nanoparticle vaccines in nonclinical and clinical studies. The SARS-CoV-2 rS nanoparticle vaccine candidate features targeted mutations to improve resistance to proteolytic cleavage and enhance retention of the prefusion conformation. It binds to the human ACE2 receptor with high affinity and exhibits good thermostability. Further details on

the study vaccine can be found in the IB (Novavax 2020).

The SARS-CoV-2 rS nanoparticle vaccine, developed by Novavax, is constructed from the full length wild-type SARS-CoV-2 S glycoprotein based upon the GenBank gene sequence MN908947, nucleotides 21563-25384. The S protein is a type 1 trimeric glycoprotein of 1,273 amino acids that is produced as an inactive S0 precursor. The S-gene was codon optimized for expression in Sf9 insect cells. The native S protein has been modified by mutation of the putative native furin cleavage site RRAR to QQAQ and insertion of 2 proline substitutions (positions K986P and V987P) in the HR1 domain. Coronavirus S proteins are metastable prefusion type I glycoproteins that undergo structural rearrangement to a post fusion form facilitating fusion of viral and host-cell membranes and infection. Mutation of the S protein furin site and 2 adjacent proline mutations in the C-terminal region of betacoronavirus S proteins is effective in stabilizing SARS-CoV-2 S in a prefusion conformation with high affinity to the human ACE2 receptor.[9] SARS-CoV-2 rS with these substitutions, as exemplified by the Novavax BV2373 construct, is resistant to proteolytic cleavage, binds to the ACE2 receptor with high affinity, and exhibits good thermostability.

The recombinant SARS-CoV-2 S genes are cloned into pBac™-1 deoxyribonucelic acid (DNA) baculovirus transfer vectors (Millipore Sigma, Burlington, Massachusetts) and co-transfected into Sf9 cells using the flashBAC™ GOLD system (Oxford Expression Technologies, Oxford, United Kingdom) using Roche – X-tremeGENE HP transfection reagent (Roche). The recombinant baculoviruses expressing the SARS-CoV-2 S protein are then identified and amplified in cell culture to produce a master virus seed.

2.3 MATRIX-M ADJUVANT

Adjuvants are compounds which, when combined with a specific vaccine antigen, serve to increase the immune response to the vaccine. In general, adjuvants work by engaging one or more component of the innate immune system, a system that provides a rapid response to infection or tissue damage based on recognition of molecular structures common to large groups of microbial pathogens.[10] Adjuvants may both quantitatively increase the antibody response and also qualitatively broaden its specificity. In addition, some adjuvants may modulate the cellular immune response.

Matrix-M is a saponin-based adjuvant, which can be co-administered with an antigen to

induce a targeted and enhanced immune response. The proposed mode of action of Matrix-M does not include a depot effect, but rather is through a combination of activities including recruitment and activation of innate immune cells to the site of vaccine injection, rapid antigen delivery to antigen-presenting cells (APCs), and enhanced antigen presentation via both major histocompatibility complex (MHC) I and MHC II molecules in the draining lymph nodes. Further details regarding the Matrix-M adjuvant are provided in the current version of the Matrix-M adjuvant safety supplement to the IB.

2.4 COVOVAX:

After technology transfer between Novavax and Serum Instiute of India Pvt Ltd., Novavax-SARS-CoV-2 rS with Matrix-M1™ Adjuvant vaccine is also manufactured at SIIPL. It is called as COVOVAX [SII-SARS-CoV-2 recombinant spike protein nanoparticle vaccine (SARS-CoV-2 rS) with Matrix-M1™ adjuvant)].

2.4.1 Summary of Nonclinical Studies:

In support of the development of SARS-CoV-2 rS, Novavax has obtained nonclinical pharmacology data concerning several SARS-CoV-2 spike protein variants, toxicity data concerning SARS-CoV-2 rS with Matrix-M, and prior toxicity data concerning other viral glycoproteins manufactured in the baculovirus-Sf9 system and formulated with Matrix-M.

2.4.2 Nonclinical Data from Other Baculovirus-Sf9-Produced Nanoparticle Vaccines that Support SARS-CoV-2 rS Development

The immunogenicity and protective efficacy of 2002-2003 SARS-CoV S protein and chimeric influenza/SARS-CoV virus-like particle (VLP) vaccines produced in the baculovirus-Sf9 system and administered with and without aluminum hydroxide adjuvants was demonstrated in a mouse challenge study. The selected target was the S protein, which mediates coronavirus attachment to host cells and, in the case of SARS-CoV, binds to the same angiotensin converting enzyme 2 (ACE2) receptor as the SARS-CoV-2 responsible for the current COVID-19 outbreak. Robust neutralizing antibody titers were observed following vaccination, although both antigens required adsorption to aluminum hydroxide for optimal responses. Following SARS-CoV challenge, there were no survivors among vehicle-treated mice, whereas 70% of mice in the lowest-dose SARS-CoV S group without aluminum, and 100% of mice from all other groups survived.[11]

Middle Eastern respiratory syndrome (MERS), arose in Saudi Arabia in 2012 and was found to be caused by another coronavirus, now termed MERS-CoV, that was resident in camels. The immunogenicity and protective efficacy of a MERS-CoV S nanoparticle vaccine with and without Matrix-M adjuvant was demonstrated in a mouse challenge study. Following vaccination, the MERS-CoV S nanoparticle was immunogenic across all active treatment groups; however, the presence of Matrix-M induced a 3 to > 10-fold enhancement of the binding and neutralizing antibody responses. In addition, Matrix-M essentially eliminated the antigen dose-response, suggesting the potential for major antigen sparing and consequent improved manufacturing efficiency and timeliness. Unlike SARS-CoV and SARS-CoV-2, which bind to the ACE2 receptor of human cells, the MERS-CoV host cell receptor is dipeptidyl peptidase IV (DPP4).[8] The MERS-CoV S protein does not bind to murine DPP4, and mice are not susceptible to MERS-CoV infection; therefore, mice were transduced with human DPP4 (hDPP4) prior to challenge. Following challenge with MERS-CoV, virus replication was significantly reduced in all immunized animals compared with placebo recipients, with virus replication almost completely blocked in animals receiving MERS-CoV S nanoparticles with Matrix-M.[12]

Protective efficacy of viral glycoproteins produced in the bacuilovirus-Sf9 system has also been shown against a highly virulent filovirus, Zaire ebolavirus (EBOV), with EBOV GP nanoparticle vaccine administered with Matrix-M adjuvant. Efficacy was demonstrated in a series of 5 nonhuman primate (NHP) active immunization and challenge studies. Apparent risk reduction was 45.7% and 82.4% among antigen-treated (0.6 to 4 μg and 5 μg, respectively) animals. Notably, of the 3 animals that died after receiving 5 μg of EBOV GP with Matrix-M adjuvant, 1 animal was deemed to have died due to causes other than EBOV disease and another had received an untried bivalent formulation. Potential risk reduction could theoretically exceed 93%.

The Matrix-M adjuvant was also shown to enhance antibody, cellular, and protective immune responses in Balb/c mice administered EBOV GP vaccine with or without Matrix-M or aluminum phosphate adjuvants.[13]

In addition, 3 Good Laboratory Practice (GLP)-compliant toxicology studies in New Zealand White (NZW) rabbits have been performed with 4 different antigens (influenza

hemagglutinins ± respiratory syncytial virus [RSV] F, Zika virus envelope dimers [ZIKV EnvD], and EBOV GP), in which up to 100 µg Matrix-M alone or with antigen was evaluated. These toxicological investigations indicated that baculovirus-Sf9-produced antigens (up to 240 µg total nanoparticle dose) with Matrix-M adjuvant (up to 100 µg) were well-tolerated in the animals tested with no evidence of toxicity suggestive of any unusual risk or target organ for toxicity. Non-adverse findings, including local injection site inflammation, enlargement of the lymph nodes draining the injection sites, and elevated serum markers of inflammation (including C-reactive protein), were transient and were considered consistent with immune system stimulation consequent to immunization.

2.4.3 Nonclinical Data from SARS-CoV-2 Spike Protein Constructs

Mouse immunogenicity studies were conducted to evaluate several SARS-CoV-2 spike protein variants and select the vaccine candidate. The BV2373 construct, a "3Q-2P" construct featuring a full-length S protein with amino acid substitutions in the S1/S2 cleavage domain furin cleavage site introduced to confer protease resistance and also 2 proline substitutions (K986P and V987P) introduced in the HR1 domain to produce a stable prefusion conformation, which is believed to maintain availability of the most neutralization-sensitive epitopes when used as a vaccine antigen,[9] was selected as the vaccine candidate. BV2373 (3Q-2P) was demonstrated to be immunogenic and elicited functional antibodies.

For the tested constructs, shallow dose responses with Matrix-M were observed, suggesting that the adjuvant may be significantly antigen-sparing in large animals and humans. When tested over a broad 1000-fold dose range in mice, BV2373 with Matrix-M adjuvant demonstrated a greater than 100-fold antigen-sparing effect. A plateau of anti-S immunoglobulin G (IgG) responses as well as human ACE2 (hACE2) binding inhibition was seen with adjuvanted 2-dose regimens at 1 µg of antigen and above in mice.

The candidate SARS-CoV-2 rS vaccine, based on the BV2373 construct, is also being evaluated in a dose titration study using baboons, as results from this animal model may be more predictive of responses in humans. Preliminary results demonstrated potentially valuable immune responses to BV2373 as assessed by anti-S IgG, hACE2-binding inhibiting antibodies, and neutralizing antibodies.

Matrix-M provided antigen-sparing, and supported induction of functional antibodies. Importantly, Matrix-M-adjuvanted BV2373 also appeared to induce strong Th1 type CD4+

T cell responses to SARS-CoV-2 spike protein which included polyfunctional effector phenotypes. IL-4 producing cells (a T helper 2 [Th2] cell marker) were not detected by enzyme-linked immunosorbent spot (ELISPOT), while data concerning Th2 responses from intracellular cytokine staining ICCS were unfortunately not informative due to technical issues in the assay. The available data in this small baboon study provided evidence that doses of 5 μg and 25 μg with 50 μg Matrix-M are the correct doses to proceed with the Phase 1/2 trial, with Matrix-M appearing critical for maximum responses. The variability of response observed across doses with multiple response parameters (IgG, ACE2 receptor and neutralization) supported advancement of this dose ranging and dose schedule into the Phase 1/2 trial.

A GLP-compliant toxicity study in the NZW rabbit is being performed to evaluate 50 μg of the SARS-CoV-2 BV2373 construct with and without 50 μg Matrix-M adjuvant. The Interim Phase provides data informing “N+1” doses relative to the planned human Phase 1/2 trial; and the Main Phase addresses “N+1” exposures relative to a primary regimen plus a potential late booster dose. At the interim sacrifice, the well-being of the test animals appeared unaltered relative to controls, with no test article related effects on mortality, overall clinical status, Draize score of the injection sites, body weight, food consumption, body temperature, ocular examination findings, organ weights, and macroscopic observations at necropsy.

Some males in the BV2373 plus Matrix-M group had minimal erythema at the injection site after Dose 3, but this resolved by 72 hours, and there were no temperature elevations outside the normal range. As seen in other programs, doses of active vaccines, especially those with Matrix-M, induced a transient rise in acute-phase reactants such as the globulin fraction, fibrinogen, and C-reactive protein. These were interpreted as non-adverse events consistent with the inflammatory response and immune stimulation secondary to vaccination. Currently available data show no systematic alterations in gross pathology or organ weights through 3 days after the third dose; histopathology is pending.

2.4.4 Clinical Summary

Novavax has tested baculovirus-Sf9-produced nanoparticle vaccines in approximately 14,732 subjects comprising older adults, young adults, and a limited number of children 2 to 5 years of age; and also including 3,075 pregnant women, with acceptable safety. Matrix-M has been given to approximately 4,200 humans (of which, approximately 2,567 humans received

nanoparticle vaccine) with acceptable short-term reactogenicity, and an unremarkable long-term safety profile.

Novavax, is also conducting a Phase 1/2 randomized, observer-blinded, placebo-controlled trial of SARS-CoV-2 recombinant spike protein nanoparticle (SARS-CoV-2 rS) (NVX-CoV2373) vaccine with and without Matrix-M™ adjuvant in healthey adults 18-59 years of age. Participants received NVX-CoV2373 with or without Matrix-M1 (n=106) or placebo (n=25) in part 1 of the study (Phase 1).[14]

Following Dose 1, tenderness and pain were the most frequent local symptoms and systemic events were individually less frequent with headache, fatigue and myalgia being reported most commonly. As expected, following Dose 2, greater reactogenicity was reported, although the majority of symptoms were reported as ≤ Grade 1. The average duration of events was < 2 days.

Unsolicited adverse events were collected through 28 days after Dose 2. There were no severe (Grade 3) unsolicited adverse events, and the vast majority of adverse events were mild and deemed not related to vaccination. No serious adverse events (SAEs) were reported and safety follow-up continues.

NVX-CoV2373 induced neutralization titers in 100% of participants; 5 µg adjuvanted dose group peak GMT: 3,906 (95% CI: 2,556; 5,970). All subjects developed anti-spike IgG antibodies after a single dose of vaccine, many of them also developing wild-type virus neutralizing antibody responses, and after Dose 2, 100% of participants developed wild-type virus neutralizing antibody responses. Both anti-spike IgG and viral neutralization responses compared favorably to responses from patients with clinically significant COVID-19 disease. Importantly, the IgG antibody response was highly correlated with neutralization titers, demonstrating that a significant proportion of antibodies were functional.

Matrix-M™ adjuvant induced robust polyfunctional CD4+ T cell responses. The adjuvant was dose-sparing, with the lower 5 µg dose of NVX-CoV2373 performing comparably with the 25 µg dose. Cellular immune responses were measured in a subset of participants, and

NVX-CoV2373 induced antigen-specific polyfunctional CD4+ T cell responses with a strong bias toward the Th1 phenotype (IFN-g, IL-2, and TNF-a).

NVX-CoV2373/Matrix-M1 was well tolerated and elicited robust immune responses (IgG and neutralization) four-fold higher than the mean observed in COVID-19 convalescent serum from participants with clinical symptoms requiring medical care and induced CD4+ T-cell responses biased toward a Th1 phenotype.[14] These findings suggest that the vaccine may confer protection and favours evaluation in clinical efficacy studies.

Part 2 (Phase 2) is designed to evaluate the immunogenicity, safety and preliminary efficacy of SARS CoV-2 rS and Matrix-M1 adjuvant in up to 1,500 healthy adults ≥ 18 to ≤ 84 years of age with more co-morbidities than the participant population in Part 1 of the study [ClinicalTrials.gov Identifier: NCT04368988]. After enrollment, 1,288 participants were randomly assigned to 1 of 4 vaccine groups or placebo, with 1,283 participants administered at least 1 study treatment. Of these, 45% were older participants 60 to 84 years. Reactogenicity was predominantly mild to moderate in severity and of short duration (median <3 days) after first and second vaccination with NVX-CoV2373, with higher frequencies and intensity after second vaccination and with the higher dose. Reactogenicity occurred less frequently and was of lower intensity in older participants. Both 2-dose regimens of 5-μg and 25-μg NVX-CoV2373 induced robust immune responses in younger and older participants. For the 2-dose regimen of 5 μg, GMTs for IgG anti-spike protein were 65,019 (95% confidence interval (CI) 55,485 to 76,192) and 28,137 (95% CI 21,617 to 36,623) EU/mL and for wild-type virus neutralizing antibody (MN_{50}%) were 2,201 (95% CI 1,343 to 3,608) and 981 (95% CI 560 to 1,717) titers for younger and older participants, respectively, with seroconversion rates of 100% in both age groups. Neutralizing antibody responses exceeded those seen in a panel of convalescent sera for both age groups. The study confirmed the phase 1 findings that the 2-dose regimen of 5-μg NVX-CoV2373 is highly immunogenic and well tolerated in younger adults. In addition, in older adults, the 2-dose regimen of 5 μg was also well tolerated and showed sufficient immunogenicity to support its use in late-phase efficacy studies.[15]
Phase 2a/b safety, efficacy and immunogenicity study in South Africa and Phase 3 efficacy study in UK are ongoing.

UK Phase 3 efficacy results[16, 17]:

The study enrolled and randomized a total of 15,187 participants underwent, and 14,039 were included in the per-protocol efficacy population. between 18-84 years of age, including 27.9% over the age of 65 and 44.6% had coexisting illnesses. The primary endpoint of the UK Phase 3 clinical trial was based on the first occurrence of PCR-confirmed symptomatic (mild, moderate or severe) COVID-19 with onset at least 7 days after the second study vaccination in serologically negative (to SARS-CoV-2) adult participants at baseline.

The primary efficacy endpoint demonstrated an overall vaccine efficacy of 89.7% (95% CI: 80.2, 94.6) with 10 cases in the vaccine group and 96 cases in the placebo group.[16] No hospitalizations or deaths were reported among the 10 cases in the vaccine group. Five cases of severe infection were reported, all of which were in the placebo group. Vaccine efficacy was 96.4% (95% CI: 73.8, 99.5) against the original virus strain and 86.3% (95% CI: 71.3, 93.5) against the B.1.1.7/501Y.V1 variant circulating in the U.K (post hoc). Reactogenicity was generally mild and transient. The incidence of serious adverse events was low and similar in the two groups.[16]

A substudy within this UK trial evaluated the safety, immunogenicity, and efficacy of NVX-CoV2373 when co-administered with licensed seasonal influenza vaccines. 431 participants were co-vaccinated with a seasonal influenza vaccine in the substudy (217 received NVX-CoV2373 plus the influenza vaccine and 214 received placebo plus the influenza vaccine). Co-administration resulted in no change to influenza vaccine immune response. NVX-CoV2373 vaccine efficacy when co-administered with seasonal influenza vaccines (participants aged 18 to <65 years) was 87·5% (95% CI -0·2 to 98·4). This study showed that efficacy of NVX-CoV2373 was not affected with seasonal influenza vaccine co-administration.[17]

South Africa Phase 2a/b interim results[18]:

In the South Africa Phase 2a/b clinical trial, 4387 received at least one injection of vaccine or placebo. 60% efficacy (95% CI: 19.9 – 80.1) for the prevention of mild, moderate and severe COVID-19 disease was observed in the 94% of the study population that was HIV-negative. Twenty-nine cases were observed in the placebo group and 15 in the vaccine group. One severe case occurred in the placebo group and all other cases were mild or moderate. The clinical trial

also achieved its primary efficacy endpoint in the overall trial population, including HIV-positive and HIV-negative subjects (efficacy of 49.4%; 95% CI: 6.1 – 72.8).[18]

<u>USA, Mexico Phase 3 results[19]:</u>

A large Phase 3, randomized, observer-blinded, placebo-controlled trial was conducted in the United States and Mexico during the first half of 2021 to evaluate the efficacy and safety of NVX-CoV2373 in adults (≥18 years of age) who had not had severe acute respiratory syndrome coronavirus 2 (SARS-CoV-2) infection.

Participants were randomly assigned in a 2:1 ratio to receive two doses of NVX-CoV2373 or placebo 21 days apart. The primary objective was to determine vaccine efficacy against RT-PCR-confirmed COVID-19 occurring at least 7 days after the second dose. Vaccine efficacy against moderate-to-severe disease and against different variants was also assessed.

Of the 29,949 participants who underwent randomization between December 27, 2020, and February 18, 2021, a total of 29,582 (median age, 47 years; 12.6% ≥65 years of age) received at least one dose: 19,714 received vaccine and 9868 placebo. Over a period of 3 months, 77 cases of COVID-19 were noted - 14 among vaccine recipients and 63 among placebo recipients (vaccine efficacy, 90.4%; 95% CI, 82.9 to 94.6; P<0.001). Ten moderate and 4 severe cases occurred, all in placebo recipients, yielding vaccine efficacy against moderate-to-severe disease of 100% (95% CI, 87.0 to 100). Most sequenced viral genomes (48 of 61, 79%) were variants of concern or interest - largely B.1.1.7 (alpha) (31 of the 35 genomes for variants of concern, 89%). Vaccine efficacy against any variant of concern or interest was 92.6% (95% CI, 83.6 to 96.7). Reactogenicity was mostly mild to moderate and transient but was more frequent among NVX-CoV2373 recipients than among placebo recipients and was more frequent after the second dose than after the first dose.

NVX-CoV2373 was safe and effective for the prevention of COVID-19.[19]

<u>Homologous booster dose in Phase 2 study in USA and Australia[8]:</u>

In a phase 2 study, a single booster dose of NVX-CoV2373 was administered to healthy adult participants 18 to 84 years of age approximately 6 months following their primary two-dose vaccination series. Safety and immunogenicity parameters were assessed, including assays for

IgG, MN_{50}, and hACE2 receptor binding inhibition against the ancestral SARS-CoV-2 strain (Wuhan) and select variants (B.1.351 [Beta], B.1.1.7 [Alpha], B.1.617.2 [Delta], and B.1.1.529 [Omicron]). This trial is registered with ClinicalTrials.gov, NCT04368988.

Immune responses seen 14 days following the primary vaccination series were compared with those observed 28 days following the booster (Day 35 and Day 217, respectively). For the ancestral SARS-CoV-2 strain, serum IgG GMTs increased ~4.7-fold from 43,905 EU at day 35 to 204,367 EU at Day 217. Neutralization (MN_{50}) assay GMTs showed a similar increase of ~4.1-fold from 1,470 at day 35 to 6,023 at Day 217.

A functional hACE2 receptor binding inhibition assay analyzing activity against ancestral and variant strains of SARS-CoV-2 at Day 189 vs Day 217 found 54.4-fold (Ancestral), 21.9-fold (Alpha), 24.5-fold (Beta), 24.4-fold (Delta), and 20.1-fold (Omicron) increases in titers. An anti-rS IgG activity assay comparing the same time points across the same SARS-CoV-2 strains found titers improved 61.2-fold, 85.9-fold, 65.0-fold, 92.5-fold, and 73.5-fold, respectively.

Following the booster, incidence rates of local and systemic reactions were 82.5% (13.4% ≥ Grade 3) and 76.5% (15.3% ≥ Grade 3), respectively, compared to 70.0% (5.2% ≥ Grade 3) and 52.8% (5.6% ≥ Grade 3), respectively, following the primary vaccination series. Events were primarily mild or moderate in severity and transient in nature, with a median duration of 1.0 to 2.5 days.

Administration of a booster dose of NVX-CoV2373 approximately 6 months following the primary vaccination series resulted in enhanced immune responses. For both the prototype strain and all variants evaluated, immune responses following the booster were notably higher than those associated with high levels of efficacy in phase 3 studies of the vaccine.[8]

2.5 RATIONALE FOR STUDY DESIGN

This is a Phase 3, observer-blinded, randomised, active controlled study in adults ≥ 18 years of age in India, to evaluate the immunogenicity and safety of COVOVAX in comparison with control vaccine in adults who have already received primary vaccination against COVID-19 at least 3 months ago (6 months / 180 days from second dose).

It has become evident that the protection offered against COVID-19 wanes after a two-dose schedule of COVID-19 vaccines, potential role of third or booster dose against COVID-19 is

being explored by several countries. Many vaccines have already received approval to be used as third dose or booster dose and currently more than 100 countries have started administering booster doses. Nuvaxovid has already been used for both homologous as well as heterologous boosting in clinical trials. Nuvaxovid has already shown that it induces robust booster response in both the regimens.[8,20]

Considering this background, this study will evaluate the booster response to COVOVAX in those individuals ≥ 18 years of age who have received 2 doses of COVID-19 vaccines at least 6 months ago.

3. OBJECTIVES AND ENDPOINTS

3.1 CO-PRIMARY OBJECTIVE(S)

Co-primary Objective(s)	Estimand Description (including Endpoint)
To compare immunogenicity of the COVOVAX booster dose in comparison with the Control vaccine by anti-S IgG for COVISHIELD Prime, and COVAXIN Prime cohorts, separately	**Estimand 1.1a (Co-primary)**<br>Geometric Mean Titers (GMTs) ratio (GMR) of anti-S IgG at 28 days after vaccination between vaccines (COVOVAX booster/ control) for each cohort<br>Principal stratum and Hypothetical strategy are used to understand antibody levels achieved through vaccination, without subsequent COVID-19/SARS-CoV-2 infection or use of any immune-modifying medications or other vaccines.<br>While on treatment strategy is used for event death.<br><br>**Co-primary endpoint 1.1:**<br>Anti-S IgG at 28 days after vaccination<br><br>**Estimand 1.1b (Co-primary)**<br>Proportion of participants achieving ≥ 2-fold increase in anti-S IgG at 28 days after vaccination between vaccines (COVOVAX booster/ control).<br>Principal stratum and Hypothetical strategy are used to understand antibody levels achieved through vaccination, without subsequent COVID-19/SARS-CoV-2 infection or use of any immune-modifying medications or other vaccines.<br>While on treatment strategy is used for event death.<br><br>**Co-primary endpoint 1.1a:**<br><br>Proportion of participants achieving ≥ 2-fold increase in anti-S IgG at 28 days after vaccination
To compare immunogenicity of the COVOVAX booster dose in comparison with the Control vaccine by neutralizing antibodies (nAbs) for COVISHIELD Prime, and COVAXIN Prime cohorts, separately	**Estimand 1.2a (Co-primary)**<br>Geometric Mean ratio (GMR) of nAbs at 28 days after vaccination between vaccines (COVOVAX booster/ control) for each cohort

Co-primary Objective(s)	Estimand Description (including Endpoint)
	Principal stratum and Hypothetical strategy are used to understand antibody levels achieved through vaccination, without subsequent COVID-19/SARS-CoV-2 infection or use of any immune-modifying medications or other vaccines.<br>While on treatment strategy is used for event death.<br>**Co-primary endpoint:**<br>nAbs at 28 days after vaccination<br><br>**Estimand 1.2b (Co-primary)**<br>Proportion of participants achieving ≥ 2-fold increase in nAbs at 28 days after vaccination between vaccines (COVOVAX booster/ control).<br>Principal stratum and Hypothetical strategy are used to understand antibody levels achieved through vaccination, without subsequent COVID-19/SARS-CoV-2infection or use of any immune-modifying medications or other vaccines.<br>While on treatment strategy is used for event death.<br><br>**Co-primary endpoint 1.2a:**<br><br>Proportion of participants achieving ≥ 2-fold increase in nAbs at 28 days after vaccination

3.2 SECONDARY OBJECTIVES AND ESTIMANDS

Secondary Objective(s)	Estimand Description (including Endpoints)
To assess the tolerability and reactogenicity profile of COVOVAX in comparison with the Control vaccine for COVISHIELD Prime, and COVAXIN Prime cohorts, separately	**Estimand 2 (for 2 and 3 listed below)**<br>**Estimand 3 (for 1 and 4 listed below)**<br>Proportion of participants with at least one<br>1. Solicited local and/or Systemic adverse event up to 7 days post-vaccination<br>2. Unsolicited AE up to 28 days post-vaccination<br>3. SAE throughout study duration post-vaccination<br>4. Adverse event of special interest post-vaccination<br>A treatment policy strategy is used for assessing safety irrespective of use of immune-modifying medications/ vaccinations.<br>An intercurrent event death will use strategy as follows:

Secondary Objective(s)	Estimand Description (including Endpoints)
	• Composite strategy is used for event death in case of SAE<br>• Solicited local and/ or systemic adverse events, unsolicited adverse events and AESI will use while on treatment strategy (i.e. data until death is utilized)<br>An intercurrent event COVID-19/SARS-CoV-2 infection will use Treatment policy for all listed above.<br>**Endpoint:**<br>1. Occurrence of solicited local and systemic adverse events (AEs) for 7 days post-vaccination<br>2. Occurrence of unsolicited AEs for 28 days post-vaccination<br>3. Occurrence of SAEs, and adverse events of special interest (AESI) throughout the study duration following vaccination
To assess immunogenicity of the COVOVAX booster dose in comparison with the Control vaccine by anti-S IgG and neutralizing antibody assays for COVISHIELD Prime, and COVAXIN Prime cohorts, separately	**Estimand 4**<br>GMTs and GMFR of anti-S IgG antibodies at 28, 90 and 180 days post-vaccination.<br>Principal stratum and Hypothetical strategy are used to understand antibody levels achieved through vaccination, without subsequent COVID-19/SARS-CoV-2 infection or use of any immune-modifying medications or other vaccines.<br>While on treatment strategy is used for event death.<br>**Endpoint:**<br>Anti-S IgG antibodies at Day 29, Day 91 and Day 181<br><br>**Estimand 5:**<br>GMTs and GMFR of nAbs at 28 and 180 days post-vaccination.<br>Principal stratum and Hypothetical strategy are used to understand antibody levels achieved through vaccination, without subsequent COVID-19/SARS-CoV-2 infection or use of any immune-modifying medications or other vaccines.<br>While on treatment strategy is used for event death.<br>**Endpoint:**<br>nAbs at Day 29 and Day 181

Secondary Objective(s)	Estimand Description (including Endpoints)
To assess immune response of COVOVAX booster dose between COVISHIELD Prime, and COVAXIN Prime cohorts by anti-S IgG and neutralizing antibody assays	**Estimand 6:**<br>GMTs of anti-S IgG antibodies of COVOVAX from COVISHILED prime and COVOVAX from COVAXIN prime at 28, 90 and 180 days after vaccination<br><br>Principal stratum and Hypothetical strategy are used to understand antibody levels achieved through vaccination, without subsequent COVID-19/SARS-CoV-2 infection or use of any immune-modifying medications or other vaccines.<br>While on treatment strategy is used for event death.<br>**Endpoint:**<br>Anti-S IgG antibodies at Day 29, Day 91 and Day 181<br><br>**Estimand 7:**<br>GMTs of nAbs of COVOVAX from COVISHILED prime and COVOVAX from COVAXIN prime at 28 and 180 days after vaccination.<br>Principal stratum and Hypothetical strategy are used to understand antibody levels achieved through vaccination, without subsequent COVID-19/SARS-CoV-2 infection or use of any immune-modifying medications or other vaccines.<br>While on treatment strategy is used for event death.<br>**Endpoint:**<br>nAbs at Day 29 and Day 181
To assess immune response of Covishield booster dose in COVISHIELD Prime and Covaxin booster dose in COVAXIN Prime cohorts by anti-S IgG and neutralizing antibody assays	**Estimand 8:**<br>GMTs of anti-S IgG antibodies at 28, 90 and 180 days after vaccination<br>Principal stratum and Hypothetical strategy are used to understand antibody levels achieved through vaccination, without subsequent COVID-19/SARS-CoV-2 infection or use of any immune-modifying medications or other vaccines.<br>While on treatment strategy is used for event death.<br>**Endpoint:**<br>Anti-S IgG antibodies at Day 29, Day 91 and Day 181<br><br>**Estimand 9:**<br>GMTs of nAbs at 28 and 180 days after vaccination.<br>Principal stratum and Hypothetical strategy are used to understand antibody levels achieved through vaccination, without subsequent COVID-19/SARS-CoV-2 infection or use of any immune-modifying medications or other vaccines.<br>While on treatment strategy is used for event death.<br>**Endpoint:**<br>nAbs at Day 29 and Day 181

3.3 EXPLORATORY OBJECTIVES

Exploratory Objective(s)	Estimand Description (including Endpoints)
To assess cell mediated immune response of the COVOVAX in comparison with the Control vaccine for COVISHIELD Prime, and COVAXIN Prime cohorts, separately	**Estimand 10:**<br>The mean of change from baseline (Day 1) in cell-mediated immune responses as measured by enzyme-linked immune absorbent spot (ELISpot) at 28 days and 180 days post-vaccination<br><br>Principal stratum and Hypothetical strategy are used to understand cell-mediated immunity achieved through vaccination, without subsequent COVID-19/SARS-CoV-2 infection or use of any immune-modifying medications or other vaccines.<br>While on treatment strategy is used for event death.<br>**Endpoint:**<br>Mean of change from Baseline (Day 1) in the cell-mediated immune responses (for example, as measured by enzyme-linked immune absorbent spot (ELISpot) ± intracellular cytokine staining) at Day 29 and Day 181.
To assess immune response by neutralizing antibodies against variants of concern for COVISHIELD Prime, and COVAXIN Prime cohorts, separately	**Estimand 11:**<br>GMTs and GMFR of nAbs against variants of concern at 28 and 180 days post-vaccination.<br>Principal stratum and Hypothetical strategy are used to understand antibody levels achieved through vaccination, without subsequent COVID-19/SARS-CoV-2 infection or use of any immune-modifying medications or other vaccines.<br>While on treatment strategy is used for event death.<br>**Endpoint:**<br>nAbs against SARS-CoV-2 variants of concern at Day 29 and Day 181

4. STUDY DESIGN

This is a Phase 3, observer-blinded, randomised, active controlled study in adults ≥ 18 years of age in India who have already received primary vaccination against COVID-19 at least 6 months ago (6 months / 180 days from the second dose), to evaluate the immunogenicity and safety of the COVOVAX booster dose in comparison with the control vaccine.

A total of 372 eligible participants of ≥ 18 years of age who have completed primary 2 dose schedule of COVID-19 vaccination at least 6 months ago will be enrolled in this study in 2 cohorts of 186 participants each with 1:1 allocation to COVOVAX or control vaccine. Two cohorts of 186 participants each will be as below:

- **COVISHIELD Prime cohort:** Primary vaccination with two doses of COVISHIELD
- **COVAXIN Prime cohort:** Primary vaccination with two doses of COVAXIN

All eligible participants (n=372) will receive a single dose of 0.5 mL of either COVOVAX or Control vaccine on Day 1 as per randomization. Post vaccination site visits are planned on Day 29, Day 91, and Day 181.

Treatment allocation:

COVISHIELD Prime cohort	COVOVAX	93
	Covishield	93
COVAXIN Prime cohort	COVOVAX	93
	Covaxin	93

Approximately 10 ml blood sample will be collected at baseline (Day 1), Day 29, Day 91 and Day 181. Additionally, up to 20 ml blood sample may be collected from subset of 36 participants (18 from each of the two prime cohorts) for assessment of CMI responses at baseline, Day 29 and Day 181.

Table 1: Schedule of study events

Visit Number	1	2	3	4
**Visit time and window**	**Day 1**	**Day 29 (+7)**	**Day 91 (+7)**	**Day 181 (+14)**
**Informed Consent**	X	NA		
**Demographic Data**	X	NA		
**Medical History including details of COVID-19 Vaccination**	X	NA		
**General Physical Examination & vital signs**	X	X[a]	X[a]	X[a]
**Urine pregnancy test[b]**	X	NA		
**Inclusion/Exclusion Criteria**	X	NA		
**Randomization**	X	NA		
**Nasopharyngeal /Nose and/or throat swab for RT-PCR**	X	X[c]		
**Blood collection for immunogenicity assessments**	X	X	X	X
**Study Vaccination**	X	NA		
**30-Minute Post-Vaccination Assessment**	X	NA		
**Recording of solicited AEs**	*Through 7 days post-vaccination*			
**Recording of unsolicited AEs**	*Through 28 days post-vaccination*		NA	
**Reporting of SAEs, AESI**	Throughout the study			
**Recording of concomitant medications and vaccinations[d]**	Throughout the study			

a. A targeted physical examination (only at post vaccination visits) will be performed if there has been any AE reported since the previous visit that has not already been recorded and closed within unscheduled visits

b. Only among female participants of child bearing potential.

c. Post-vaccination: If the participant presents with qualifying symptoms of suspected COVID-19 disease [Appendix II, Table 1] OR history of contact with a confirmed COVID-19 positive case then a swab from nasopharynx / nose and/or throat will be collected for RT-PCR testing for SARS-CoV-2 infection.

d. Beyond Day 29, only the concomitant medications indicated for SAEs and AESI, if any will be recorded

5. STUDY POPULATION

5.1 INCLUSION CRITERIA

Eligible participants must meet all of the below criteria at the time of enrolment:

1. Adults aged ≥ 18 years of either sex
2. Written informed consent by participants
3. Those who have completed primary COVID-19 vaccination schedule with either Covishield or Covaxin (2 doses) at least 6 months ago (6 months / 180 days from the second dose)
4. The participant is resident of the study area and is willing to comply with study protocol requirements, including availability for all scheduled visits of the study
5. Healthy / clinically stable condition, as determined by medical history and physical examination
6. Sexually active female participants of childbearing potential* must have practiced adequate contraception for 28 days prior to study vaccine administration and agree to continue adequate contraception until completion of 28 days after vaccination

   * Females can be considered not of childbearing potential only if they have undergone bilateral tubal ligation or occlusion, or hysterectomy, or bilateral oophorectomy, or are post- menopausal (defined as continuous amenorrhea for 12 months)
7. Female participants of childbearing potential must have a negative urine pregnancy test within 24 hours prior to study vaccine administration

5.2 EXCLUSION CRITERIA

Participants meeting any of the below criteria at the time of enrolment will be ineligible to participate in the trial:

1. Acute illness including COVID-19 with or without fever at the time of screening
2. History of laboratory confirmed (by RT-PCR, rapid antigen test or serology to SARS-CoV-2) COVID-19
3. History of allergic reactions after previous vaccinations
4. Hypersensitivity to any component of study vaccines
5. Any confirmed or suspected condition with impaired/altered function of immune system (e.g. immunodeficient or autoimmune conditions)

Note: Stable endocrine disorders that have a confirmed autoimmune etiology (e.g., thyroid, pancreatic) are allowed.

6. Have any bleeding disorder which is considered as a contraindication to intramuscular injection or blood draw
7. Suspected or known current alcohol or drug dependence
8. Chronic administration (defined as more than 14 continuous days) of immunosuppressant or other immune-modifying drugs within three months prior to the study vaccination or planned use throughout the study period (For corticosteroids, this means prednisone, or equivalent, ≥ 0.5 mg/kg per day. Inhaled, intranasal and topical steroids are allowed)
9. Administration of blood, blood products and/or plasma derivatives or any parenteral immunoglobulin preparation in the past 3 months or planned use throughout the study period
10. Continuous use of anticoagulants, such as coumarins and related anticoagulants (i.e. warfarin) or novel oral anticoagulants (i.e. apixaban, rivaroxaban, dabigatran and edoxaban etc)
11. Administration of any vaccine within 28 days prior to enrolment in the study or planned administration of any vaccine for until 28 days after study vaccination
12. Planning to receive a booster dose of COVID-19 vaccine during the course of the study
13. Prior receipt of a booster dose of COVID-19 vaccine
14. Use of any investigational or non-registered drug or vaccine within 30 days prior to the administration of study vaccines or planned during the study
15. Pregnant or breast-feeding
16. Acute or chronic, clinically significant pulmonary, cardiovascular, endocrine, metabolic, gastrointestinal, neurological, hepatic, renal functional abnormality or any other systemic disorder, that are assessed by the investigator (based on medical history or physical examination) as being clinically unstable within the prior 4 weeks (mild or moderate well-controlled comorbidities are allowed)
17. History of chronic neurological disorders such as multiple sclerosis, dementia, transient ischemic attacks, Parkinson’s disease, degenerative neurological conditions, neuropathy, and epilepsy or a history of stroke or previous neurological disorder within 12 months with residual symptoms

Note: Participants with a history of migraine or chronic headaches or nerve root compression that have been stable on treatment for the last 4 weeks not to be excluded

18. Any other condition that in the opinion of the investigator would jeopardize the safety or rights of the participant in the study or make it unlikely that the participant could complete the protocol

6. TREATMENT OF STUDY PARTICIPANTS

6.1 DESCRIPTION OF STUDY VACCINES

The term 'study vaccine' refers to those vaccines provided by the Sponsor, which will be evaluated as part of the study objectives. The study vaccines specific to this study are described below.

Study Vaccines

**COVISHIELD Prime cohort**	**Test vaccine**	COVOVAX [SARS-CoV-2 recombinant spike protein nanoparticle vaccine (SARS-CoV-2 rS) with Matrix-M1™ adjuvant]
	**Control**	Covishield™
**COVAXIN Prime cohort**	**Test vaccine**	COVOVAX [SARS-CoV-2 recombinant spike protein nanoparticle vaccine (SARS-CoV-2 rS) with Matrix-M1™ adjuvant]
	**Control**	Covaxin™

6.1.1 Test vaccine:

COVOVAX [SARS-CoV-2 recombinant spike protein nanoparticle vaccine (SARS-CoV-2 rS) with Matrix-M1™ adjuvant] is available as a ready to use liquid formulation in a 10-dose (5.0 mL) vial. Each dose of 0.5 mL contains 5 µg antigen and 50 µg Matrix-M1 adjuvant. The other ingredients are 25 mM phosphate buffer (pH 7.2), 300 mM sodium chloride, and 0.01% (v/v) polysorbate 80.

- **Manufacturer:** Serum Institute of India Pvt. Ltd. (SIIPL)
- **Formulation**: Ready to use liquid formulation in a 10-dose vial.
- **Route of administration:** Intramuscular
- **Site of injection:** Deltoid muscle
- **Dose:** 0.5 ml containing 5 µg antigen and 50 µg Matrix-M adjuvant
- **Dose schedule:** Single dose on Day1

6.1.2 Comparator product [Covishield™ for Covishield prime cohort and Covaxin for Covaxin prime cohort]:

Covishield™:

One dose (0.5 ml) contains:

ChAdOx1 nCoV- 19 Corona Virus Vaccine (Recombinant) 5×10^{10} virus particles (vp)

*Recombinant, replication-deficient chimpanzee adenovirus vector encoding the SARS-CoV-2 Spike (S) glycoprotein. Produced in genetically modified human embryonic kidney (HEK) 293 cells.

- **Manufacturer:** Serum Institute of India Pvt. Ltd.
- **Formulation:** Ready to use formulation in multi-dose glass vials
- **Route of administration:** Intramuscular
- **Site of injection:** Deltoid muscle
- **Dose:** 0.5 ml.
- **Dose schedule:** Single dose on Day 1.

Covaxin®:

Each dose of 0.5mL contains:

Ingredient	Quantity
Whole Virion, Inactivated Coronavirus (SARS-CoV-2) Antigen (Strain: NIV-2020-770)	6 µg
Aluminium Hydroxide Gel equivalent to Al+++	0.25 mg
TLR7/8 Agonist	15 µg
2-Phenoxyethanol	2.5 mg
Phosphate Buffered Saline	q.s. to 0.5 mL

- **Manufacturer**: Bharat Biotech International Limited
- **Formulation**: Ready to use formulation in glass vials
- **Route of administration**: Intramuscular
- **Site of injection**: Deltoid muscle
- **Dose**: 0.5 ml.
- **Dose schedule**: Single dose on Day 1.

6.2 PRECAUTIONS TO BE OBSERVED IN ADMINISTRATING STUDY VACCINES

Prior to vaccination, participants must be determined to be eligible for study vaccination and it must be clinically appropriate in the judgment of the investigator to vaccinate.

Study vaccines should not be administered to individuals with known hypersensitivity to any component of the vaccines.

Standard immunization practices are to be observed and care should be taken to administer the injection intramuscularly. Before administering the study vaccine, the vaccination site is to be disinfected with a skin disinfectant (e.g., 70% alcohol). Allow the skin to dry. DO NOT inject intravascularly.

As with all vaccines, appropriate medical treatment (like adrenaline 1:1000, anti-histamine (diphenhydramine), corticosteroids (hydrocortisone) and resuscitation equipment etc.) must be available at the site, and staff and supervision must be readily available in case of rare anaphylactic or any severe allergic reactions following administration of the study vaccine.

Prompt use of resuscitation measure can be lifesaving and must be implemented at the first suspicion of anaphylaxis.

6.3 PREPARATION AND ADMINISTRATION OF THE STUDY VACCINE

Study vaccines are available as a ready to use vial / ampoule and does not need any reconstitution. A vial / ampoule will be removed from cold storage and inspected to confirm the absence of particulate materials and 0.5 mL volume from vial / ampoule will be withdrawn using needle and syringe and injected intramuscularly.

Study vaccine should be visually inspected before administration and in the event of any foreign particulate matter and/or any unusual appearance of the study vaccine, vial / ampoule will be set aside and replacement vial / ampoule should be used.

The study vaccine will be administered as per randomization schedule via intramuscular injection on Day 1. Preferred site of injection is deltoid muscle. The study vaccines are supplied as 10-dose vials for COVOVAX, and single dose ampoule / multi-dose vials for control vaccine. The used vial / ampoule will be kept securely at site for accountability by study monitor.

The investigator or designee will be responsible for oversight of the administration of vaccine to participants enrolled in the study according to the procedures presented in this study

protocol. All vaccines will be prepared and administered only by designated personnel who are qualified to perform that function.

Study vaccine to be administered to the participants must be stored in a safe and locked place with no access by unauthorized personnel.

6.4 VACCINE SUPPLY, LABELLING, STORAGE, ACCOUNTABILITY AND DISPOSAL

The sponsor will ensure the following:

- Appropriate supply of the study vaccines;
- Appropriate labeling of all study vaccines that complies with regulatory requirements.

The investigator must ensure the following:

- Availability of appropriately trained site staff to manage vaccine supply, accountability, preparation and administration.
- Acknowledge receipt of the study vaccines by site staff, including confirmation that the vaccines:
  - were received in good condition;
  - remained within the appropriate temperature range during shipment from the sponsor to the investigator's designated storage location;
  - have been confirmed by the sponsor as authorized for use
- Proper storage of the study vaccines, including:
  - storage in a secure, locked, temperature-controlled location;
  - proper storage according to the instructions specified on the labels;
  - appropriate record keeping and inventory of the study vaccines, including regular documentation of adequate storage temperature
- Appropriate use of the study vaccines, including:
  - use only in accordance with the approved protocol;
  - proper handling, including confirmation that the vaccine has not expired prior to administration;
  - appropriate documentation of administration of vaccines to study participants including:

  - Date, dosage, batch number, screening number assigned to participants, and time of vaccine administration. This information will be maintained in an accountability log that will be reviewed by the site monitor;
  - Proper reconciliation of all study vaccines received from the sponsor. Reconciliation is defined as maintaining records of which and how many vaccines were received, which vaccines (and volume thereof) were administered to participants, and which vaccines were destroyed at the site.
- Vaccine will be either destroyed at site after sponsor approval or can be returned back to sponsor. Site will provide adequate documentation of destruction in the former case.

The study vaccines will be stored at +2ºC to +8ºC in a secure refrigerator. The storage temperature of the vaccines will be monitored daily with temperature monitoring devices and will be recorded.

Vaccines that have been stored differently from the manufacturer's instructions must not be used unless the sponsor provides written authorization for use. Any temperature deviation, i.e. temperature outside the range, must be reported to the sponsor as soon as detected. Following the exposure to such a temperature deviation, vaccines will not be used until written approval has been given by the sponsor. Expired vaccines must not be administered.

In the event that the use cannot be authorized, the sponsor will make every effort to replace the vaccine supply. Monitoring of vaccine accountability will be performed by the study monitor during site visits and at the completion of the trial.

7. STUDY PROCEDURES

7.1 GENERAL CONSIDERATIONS

The study will be initiated only after approvals from each site's Institutional ethics committee (IEC) and the DCGI have been obtained.

The schedules of evaluations and procedures that must be performed at specific time points are described in the following sections and in Tables 1: Schedule of study events.

At each visit participants need to be counselled thoroughly to follow all the standard measures like wearing mask, social distancing, regular hand washing, using of hand sanitizer etc. as per the health authority guidelines to prevent getting infected with SARS CoV-2.

7.2 STUDY VISITS

Visit #1 – Screening, Randomization and Vaccination

Potential participants will be informed about the scope of the study and the possibility of their inclusion in the study. If they are willing to participate, informed consent will be obtained. A signed (or thumb print with witness signature) and dated informed consent must be obtained by the principal investigator (PI) or the designee before initiating any study specific procedures. The informed consent document used for the purpose must be approved by respective site IEC.
The process of obtaining informed consent should be documented in the source documents in addition to maintaining the original signed and dated informed consent at the site. A copy of the consent form will be given to the participants.

The participants will be screened for eligibility by the PI / designated site staff under the direction of the PI after the informed consent process has been completed. All participants screened for the study will be assigned a screening number.

All participants who have consented will be evaluated for eligibility.
The following procedures will be completed for each participant to confirm eligibility for the study:

- Demography (Age, sex, height and weight)
- Details of COVID-19 vaccination (Name of vaccine and dates of vaccination - Dose 1 and Dose 2)
- Medical History (significant past and concurrent conditions, family history, history of allergies and vaccinations, history of COVID-19)
- Complete physical Examination [general, respiratory, cardiovascular system, and central nervous system] including vital signs measurements (temperature, resting blood pressure, pulse and respiratory rate)
- Relevant prior and Concomitant medications
- In case of female participants of child bearing potential, urine pregnancy test (UPT) will be performed prior to randomization.

The participants who are ineligible for the study or not randomized will be documented as screen failures on the Screening Log and/or eCRF. The reason for screen failure must be documented.

A complete review of inclusion/exclusion criteria will be conducted. Participants who satisfy all inclusion criteria and none of the exclusion criteria will be enrolled.

Randomization:

The eligible participants will be randomized on the same day. The eligible participants will be randomized via an Interactive Web Response System (IWRS).

If for any reason, after signing the informed consent form, the participant (who has passed screening) fails to be randomized, the reason for not being randomized should be recorded in source documents.

Blinding:

The study is designed as an observer-blind study. The study participants and the study personnel responsible for the evaluation of any study endpoints (e.g. safety) will be unaware which study vaccine is administered. At each site, only designated study personnel will be involved in getting randomization code by accessing IWRS, vaccine preparation and administration. The unblinded personnel involved in study vaccine preparation and administration will not participate in any of the study end-point evaluations. All other site personnel will remain blinded to the treatment allocation.

The sponsor personnel involved in the study will also remain blinded until 28 days after the booster dose. At the interim analysis of data at 28 days after study vaccination, the sponsor personnel will become unblinded to group-wise data. Individual level blinding will still be maintained for the sponsor personnel until study completion i.e. final database lock. The CRO will designate an unblinded monitor(s) and a statistician who may be able to access the subject level unblinded data as per the need. Other CRO personnel working on the trial will remain blinded.

The laboratories involved in the immunological testing will be blinded to the treatment allocation.

Prior to Randomization and Vaccination:

If the participant meets all the eligibility criteria, a swab from nasopharynx /nose and/or throat will be collected for RT-PCR testing to detect SARS-CoV-2 infection to assess baseline status. Approximately 10 ml blood will be collected from participants for anti-S IgG and neutralizing antibody assessment prior to vaccination on Day 1. Additionally, up to 20 ml blood sample

may be collected from subset of 36 participants (18 from each of the two prime cohorts) for assessment of CMI responses.

Study Vaccination: The eligible participant will receive 0.5 ml of either COVOVAX or Control vaccine as per the randomization schedule.

Post - Vaccination Activities:

The participants will be observed closely for at least 30 minutes following vaccination, with appropriate medical treatment readily available in case of a rare anaphylactic reaction following the administration of vaccine. After 30 minutes (+15 minutes) post vaccination, any unsolicited AEs and Vitals (temperature, resting blood pressure, pulse and respiratory rate) will be recorded.

The participants will receive a digital thermometer, measuring scale and a diary. These participants will be trained by the site personnel for recording and documenting any solicited AEs within 7 days after vaccination and any other AEs they may experience until 28 days after vaccination and concomitant medications they may use in the diary. The participants will be informed to visit the site on Day 29 and carry this completed diary at the time of visit.

The investigator or a delegate should ensure that all information is recorded in the source documents and accurately transcribed in the eCRF as soon as possible after the participant's visit has been completed.

The participant will be reminded to contact the site if there is any health problem / illness or if they have any questions and to return to the clinic on Day 29.

Visit #2 (Day 29 [+7]):

Study participants will return for follow-up evaluations to the clinical study site 28 days after the vaccination. At this visit, investigator or delegate will perform the following procedures and evaluations in the following order:

1. Review and retrieval of diary card records
2. Assessment of any ongoing solicited AEs (note that all ongoing solicited AEs must be followed up by site staff until resolution)
3. Medical interview of participant to assess any unsolicited AEs/SAEs/AESI since previous study visit
4. Collection of concomitant medications and vaccinations history

5. Targeted physical examination including assessment of vital signs
6. Collection of blood sample (approximately 10 mL) for anti-S IgG and neutralizing antibody assessment. Additionally, up to 20 ml blood sample may be collected from subset of 36 participants (18 from each of the two prime cohorts) for assessment of CMI responses.

Occurrence of COVID-19 disease: If any of the study participants experience signs/symptoms suggestive of COVID-19 or history of exposure to a confirmed case of COVID-19, RT-PCR test will be performed. If the participant is confirmed with COVID-19 after the study vaccination, this will be documented in the source and eCRF.

Visit # 3 (Day 91 [+7]):

Study participants will return for follow-up evaluations to the study site 90 days following study vaccination. At this visit, investigator or delegate will perform the following procedures and evaluations in the following order:

1. Medical interview of participant to determine if any SAE/ AESI occurred and if any concomitant medications were taken/ received since the last study visit.)
2. Targeted physical examination including assessment of vital signs.
3. Collection of blood sample (approximately 10 mL) for anti-S IgG antibody assessment.

The investigator or a delegate should ensure that all information is recorded in the source documents and accurately transcribed in the eCRF as soon as possible after the participant's visit has been completed.

The participant will be reminded to contact the site if there is any health problem / illness or if they have any questions and to return to the clinic on Day 181.

Visit #4 (Day 181 [+14]): End of study visit

Study participants will return for follow-up evaluations to the study site at 6 months following vaccination. At this visit, investigator or delegate will perform the following procedures and evaluations in the following order:

1. Medical interview of participant to determine if any SAEs/ AESI occurred and if any concomitant medications or vaccines were taken/ received since the last study visit.)
2. Targeted physical examination including assessment of vital signs.

3. Approximately 10 ml blood will be collected from participants for anti-S IgG and neutralizing antibody assessment. Additionally, up to 20 ml blood sample may be collected from subset of 36 participants (18 from each of the two prime cohorts) for assessment of CMI responses.

The investigator or a delegate should ensure that all information is recorded in the source documents and accurately transcribed in the eCRF as soon as possible after the participant's visit has been completed.

In case there are no ongoing SAEs, after this visit, the participation in the study will be completed. Source records will be completed and all information will be recorded in the eCRF (including "end of study" page).

Unscheduled Visits

Unscheduled visits may be performed at participant's requests or directly by the study site when the investigator or a delegate considers it necessary for diagnosis and/or management of a finding or an AE. All unscheduled visits will be recorded in source and eCRF.

7.3 TESTING FOR COVID-19 DURING THE STUDY PERIOD:

If the participant presents with qualifying symptoms of suspected COVID-19 disease [Appendix II, Table 1] OR history of contact with a confirmed COVID-19 positive case then a swab from nasopharynx / nose and/or throat will be collected for RT-PCR testing for SARS-CoV-2 infection.

At the COVID-19 testing visit, a swab from nasopharynx /nose and/or throat, vital signs and other clinical data will be taken. Symptomatic cases will be managed as per national guidelines.

7.4 PARTICIPANT DISCONTINUATION

Participant discontinuation from study procedures prior to completion of the last study visit may occur for any of the following reasons:

- Dropout (defined as discontinuation initiated by a participant): Participation in the study is strictly voluntary. Participants have the right to withdraw their consent from study participation at any time and for any reason, without penalty. The participant may also initiate discontinuation due to an adverse event.

- Investigator-initiated: The study investigator may, at their discretion, discontinue a participant from the study if they consider it to be in the participant's best interest to do so (e.g., for safety concerns), or if the participant does not comply with the study requirements.
- Lost to follow-up: For participants who fail to attend scheduled visits, study staff are to make at least three attempts to contact the participant prior to considering the participant as lost to follow-up. These attempts should be recorded in the source documents.
- Sponsor-initiated: For example, if the sponsor is obliged to end the study for administrative or any other reasons.
- Ineligibility (either arising during the study or retrospectively, having been overlooked at screening).

Participants who discontinue prior to administration of the vaccine will be replaced, whereas those withdrawn after administration of the vaccine will not be replaced.

The reason for and date of participant discontinuation will be documented in the source documents and relevant electronic Case Report Form (eCRF). Before entering any category as the reason for the participant's discontinuation from the study, the investigator should make every effort to investigate whether an AE may have been related to the participant's discontinuation from the study. If an AE has been associated with the discontinuation, this must be described on the discontinuation eCRF page, even if it is not the primary reason for the participant's withdrawal. For participants considered lost to follow-up, the discontinuation date for the participant to be captured on the discontinuation eCRF page is the date of the participant's last completed study visit.

In the event of participant discontinuation from the study, reasonable efforts should be made to conduct the following procedures (unless participant consent to do so has been withdrawn):

- Update any AE/SAEs that remained ongoing at the time of the participant's last visit prior to discontinuation.
- If within the protocol defined reporting period, collect any new AE/SAEs and concomitant medications since the participant's last visit and the time of discontinuation.
- Update participant contact information.

The sponsor or the investigator (following consultation with the sponsor) has the right to discontinue this study at any time. The study may be discontinued at one site or across multiple sites. If the clinical study is prematurely terminated at any of the site, the investigator of the respective site is to promptly inform the study participants and respective IEC and should assure appropriate therapy and follow up for the participants. All procedures and requirements pertaining to the archiving of study documents should be followed. All other study materials (study medication/vaccines, etc.) must be returned to the sponsor.

7.5 MANAGEMENT OF PREGNANCY DURING STUDY

If a female participant becomes pregnant following administration of vaccine, she will be encouraged to complete remaining visits and study procedures unless medically contraindicated, and if possible and agreed to by the participant, she will continue to be followed for pregnancy outcome. The pregnancy and its outcome will be documented, even if birth occurs after the scheduled end of the study for the participant.

7.6 PRIOR AND CONCOMITANT THERAPY

7.6.1 Prior Medications and Vaccines

Any medications (including vaccines) that were administered to the participant within 30 days prior to the study vaccination will be considered as prior medications for this study. These will be recorded in the eCRF.

7.6.2 Concomitant Medications and Vaccines

At each study visit, the investigator/designee will ask the participants about any prescription or over-the-counter medication(s) taken since the last visit. Any medications taken at any time during the study period must be recorded on source documents and the eCRF with trade and/or generic name, indication, dose, start and end dates until Day 29. Beyond Day 29, only medications taken for SAE / AESI will be recorded.

Any treatments and/or medications specifically contraindicated, e.g., any investigational or non-registered product, any immunosuppressant and immune-modifying drug including systemic steroids, any immunoglobulin and blood product should be checked at each study visit subsequent to the study vaccination. If any become applicable during the study, it will not

require withdrawal of the participant from the study but may determine a participant's evaluability in the per-protocol analysis.
Any vaccine not foreseen in the study protocol in the period starting at Visit 1 (Day 1) and ending at end of study visit must be recorded in the eCRF.

8. ASSESSMENTS OF IMMUNOGENICITY AND DISEASE INCIDENCE

8.1 IMMUNOGENICITY:

Immunogenicity will be assessed by anti-spike (S) protein IgG antibodies to SARS-CoV-2-Spike antigen by electrochemiluminescence (ECL) using MSD or any other appropriate assay and neutralizing antibodies by using microneutralization (MN) assay.

Immunogenicity testing will be performed in compliance with GCP and GCLP requirements at the following laboratories:

- Anti-S IgG antibodies testing at Christian Medical College, Vellore, India
- Neutralizing antibodies testing at 360biolabs, Burnet Institute, Melbourne, Australia.
- Cell mediated immune (CMI) response assays at NARI-ICMR, Pune, India

Any other laboratories as appropriate may be used, if required.

8.2 INCIDENCE OF COVID-19:

If the participant presents with qualifying symptoms of suspected COVID-19 disease [Appendix II, Table 1] OR history of contact with a confirmed COVID-19 positive case then a swab from nose and/or throat will be collected for RT-PCR testing for SARS-CoV-2 infection. These samples will be processed for SARS CoV-2 RT-PCR testing at respective study sites.
Severe COVID-19 disease will be defined as per criteria described in Appendix II, Table 2. Detailed clinical parameters will be collected from medical records. These are likely to include, but are not limited to, oxygen saturation, need for oxygen therapy, respiratory rate and other vital signs, need for ventilatory support, X-ray and CT scan imaging and blood test results, amongst other clinically relevant parameters.

8.3 METHODS FOR PROCESSING, LABEL AND STORAGE OF BLOOD SAMPLES

Approximately 10 mL of blood will be drawn On Day 1, Day 29, Day 91 and Day 181 for anti-S IgG and neutralizing antibody assessments. Additionally, up to 20 ml blood sample may be

collected from subset of 36 participants (18 from each of the two prime cohorts) for assessment of CMI responses on Day 1, Day 29 and Day 181.

The blood will be processed and sera / CMI samples will be aliquoted according to the Laboratory Manual. All aliquots for anti-S IgG and neutralizing antibodies will be stored at a temperature of -20°C or below. CMI samples will be stored at -70°C or below or in Liquid nitrogen as applicable. Each aliquot (Cryotube / cryovial) will be labeled with the labels provided by Sponsor/designee. All samples will be sent to the Sponsor or Sponsor designated laboratory.

Complete instructions for labeling and storage of samples will be provided in the Laboratory Manual.

9. ASSESSMENT OF SAFETY

9.1 SAFETY MONITORING

The Investigators at each study site will be responsible for continuous close safety monitoring of all study participants, and for alerting the protocol team if concerns arise. An internal team - the Protocol Safety Review (PSRT) will be set up for safety monitoring of the trial participants.

9.2 PROTOCOL SAFETY REVIEW TEAM (PSRT)

Safety will be monitored during the study by on-site clinical staff and routinely by the PSRT, an internal group of physicians which includes the SIIPL Medical Officers, a biostatistician and designated pharmacovigilance medical officer from the CRO. The PSRT may seek independent expert medical opinion as dictated by the occurrence of certain events. There will be periodic reviews of accruing safety data by the PSRT.

9.3 ADVERSE EVENT (AE)

An AE is any untoward medical occurrence in a participant after administration of the vaccine and that does not necessarily have a causal relationship with the vaccine. An AE can therefore be any unfavorable or unintended sign (including an abnormal laboratory finding), symptom, or disease temporally associated with the use of the vaccine, whether or not related to the vaccine. This definition includes exacerbations of pre-existing conditions. Stable pre-existing conditions which do not change in nature or severity during the study are not considered AEs; however, these should be reported as part of the medical history at screening.

Adverse events that may be related to the study vaccine are listed in the prescribing information for each product.

Solicited AEs are pre-specified local and systemic AEs that occur relatively more frequently or are known to be associated with immunization, which are monitored actively as potential indicators of vaccine reactogenicity. Investigators will not be required to assess causality of solicited AEs if the onset is during the solicitation period.

The following specific solicited adverse events will be monitored for this study:

- Local reactions at injection site: Pain, tenderness, erythema, swelling, and induration
- Systemic reactions: Fever, headache, fatigue, malaise, arthralgia, myalgia, nausea and vomiting

Unsolicited AEs are any AEs reported spontaneously by the participant, observed by the study staff during study visits or those identified during review of medical records or source documents. Solicited AEs with an onset after the seven-day solicitation period will be considered unsolicited AEs.

9.4 SERIOUS ADVERSE EVENT (SAE)

An SAE is any AE that results in any of the following outcomes:

- Death
- Is life-threatening (i.e., the participant was, in the opinion of the investigator, at risk of death at the time of the event; it does not refer to an event which hypothetically might have caused death if it were more severe)
- Requires inpatient hospitalization or prolongation of existing hospitalization
- Results in persistent or significant incapacity or substantial disruption of the ability to conduct normal life functions
- Congenital anomaly or birth defect
- Important medical event that may not result in one of the above outcomes but may jeopardize the health of the study participant or may require medical or surgical intervention to prevent one of the outcomes listed above

9.5 REPORTING PERIOD AND PARAMETER

Solicited AEs will be collected through 7 days following study vaccination using participant diary card. Solicited AEs with onset during the seven-day solicitation period that continue

beyond the seven-day period will be reported as solicited AEs. Solicited AEs with onset after the seven-day solicitation period will be reported as unsolicited AEs.

Unsolicited AEs will be collected through 28 days (Day 29) following administration of study vaccine. SAEs and AESIs including PIMMCs and AESIs relevant to COVID-19 including possible vaccine-enhanced disease will be collected following administration of the study vaccine until completion of the Day 181 study visit.

Any untoward medical occurrence in a participant prior to administration of the vaccine but after signing the informed consent form, which is assessed by the investigator as being related to a study procedure, must also be documented and reported to the Sponsor.

9.6 ADVERSE EVENTS OF SPECIAL INTEREST (AESI)

Subjects will be assessed for diagnosis of an AESI at all study visits. AESIs include PIMMCs, AEs specific to COVID-19, or other potential AEs that may be determined at any time by regulatory authorities as additional information concerning COVID-19 is obtained. Listings of AESI are presented in Appendix I.

9.7 SEVERITY OF ADVERSE EVENTS

The grading scales cited below will be used to interpret the severity of each AE as such:

Grade 1 = Mild

Grade 2 = Moderate

Grade 3 = Severe (a severe AE is not necessarily an SAE, unless it meets one of the criteria that define an SAE; likewise, all SAEs are not necessarily by definition severe)

Grade 4 = Potentially Life-threatening (life-threatening AEs are to be reported as SAEs)

Grade 5 = Death

The severity of all AEs, listed specifically as an event in the Division of AIDS (DAIDS) Table for Grading the Severity of Adult and Pediatric Adverse Events, corrected version 2.1, July 2017, of the US National Institute of Health, will be assessed based on this Table, which is provided as Appendix III to this protocol and is currently also available at: https://rsc.niaid.nih.gov/sites/default/files/daidsgradingcorrectedv21.pdf

The following grading scale should be used to grade the severity of all unsolicited AEs that are not listed as a specific event in the DAIDS Table cited:

Grade 1= Causes no or minimal interference with usual social & functional activities

Grade 2 = Causes greater than minimal interference with usual social & functional activities

Grade 3 = Causes inability to perform usual social & functional activities

Grade 4 = Potentially life-threatening symptoms causing inability to perform basic self-care functions with intervention indicated to prevent permanent impairment, persistent disability

Grade 5 = Death

9.8 CAUSALITY OF ADVERSE EVENTS

The investigator will determine the causal relationship between the vaccine and the AE for all unsolicited AEs. The causality assessment is made based on the available information at the time of reporting and can subsequently be changed according to follow-up information. Causality determination is based on clinical assessment and should take into consideration the following factors:

- Is there a temporal relationship between the event and administration of the vaccine?
- Is there a plausible biological mechanism for the vaccine to cause the AE?
- Is there a possible alternative etiology for the AE, such as a concurrent illness or a concomitant medication?
- Are there previous reports of similar AEs associated with the vaccine or other vaccines in the same class?

For this study, the investigator must classify the causality of the AE according to the categories defined below:

Related: There is a reasonable possibility that the vaccine caused the event. 'Reasonable possibility' means that there is evidence to suggest a causal relationship between the vaccine and the AE.

Not Related: There is not a reasonable possibility that the administration of the vaccine caused the event.

All solicited AEs within 7 days of each vaccination will be considered as related AEs unless there is reasonable possibility of any systemic solicited AE being caused by any other concurrent condition / disease. In addition, related SAEs will be evaluated by the investigator for "expectedness" also. An unexpected AE is one that is not listed in the current Summary of

Product Characteristics / prescribing information or the IB or it is an event that is by nature more specific or more severe than a listed event.

9.9 FOLLOW-UP OF ADVERSE EVENTS

All AEs should be followed by the investigator or their designee until the event is resolved or determined to be irreversible, chronic, or stable by the investigator or participant is lost to follow up (including death). The investigator must ensure that any participants with AEs ongoing at study completion are advised or referred appropriately for continuation of care.

The outcome of an AE will be assessed as at the time of last observation per the following categories:

- Recovered without sequelae
- Recovered with sequelae
- Ongoing
- Death
- Unknown

9.10 GENERAL GUIDANCE ON REPORTING ADVERSE EVENTS

To improve the quality and precision of AE data, the investigator should observe the following guidelines:

- AEs must be graded, assessed for severity and causality, and reviewed by a site investigator.
- Whenever possible, use recognized medical terms when reporting AEs and avoid the use of colloquialisms or abbreviations.
- If known, report the diagnosis (i.e., syndrome or disease) rather than component symptoms, signs or laboratory values (e.g., report congestive heart failure rather than dyspnoea, rales, and cyanosis); however, symptoms or signs that are considered unrelated to an observed syndrome or disease should be reported as individual AEs (e.g., if congestive heart failure and severe headache are observed at the same time, each event should be recorded as an individual AE).
- AEs occurring secondary to other events (e.g., sequelae) should be identified by the primary cause. A 'primary' AE, if clearly identifiable, generally represents the most accurate clinical term to report. For example: orthostatic hypotension → fainting and fall

to floor → head trauma → neck pain; the primary AE is orthostatic hypotension, which is what should be reported. If a primary SAE is reported, events occurring secondary to the primary event should be described in the narrative description of the case.

- Death is an outcome of an event. The event that resulted in the death should be reported as the SAE.
- For hospitalizations for surgical or diagnostic procedures, the illness leading to the surgical or diagnostic procedure should be recorded as the SAE, not the procedure itself. The procedure should be captured in the case narrative as part of the action taken in response to the illness.
- Elective surgical or diagnostic procedures with or without hospitalizations (e.g., circumcision or elective abortion of a pregnancy) will not be recorded as an AE. The procedure should be captured in the case narrative as part of medical history.
- A pregnancy in a participant is not in and of itself an AE.

9.11 REPORTING OF SAE

Any SAE occurring in a study participant during the study (after vaccine administration) must be reported. Information about all SAEs will be collected and recorded in SAE form. To ensure participant safety, each SAE must be reported by the Investigator to the Sponsor within 24 hours of learning of its occurrence, even if it is not felt to be treatment-related.

The SAE form will always be completed as thoroughly as possible with all available details of the event. Even if the investigator does not have the entire recommended minimum information regarding a SAE, the SAE should still be submitted to sponsor, DCGI and respective IEC within 24 hours. Once additional relevant information is received, the SAE form should be updated. Reporting procedures will be followed as per the New Drugs and Clinical Trials Rules, 2019.

The investigator will always provide an assessment of causality at the time of the initial report.

Instructions for reporting of SAEs

The recommended minimum information required for the initial SAE report is:

- Identifiable study participant
- A suspect medicinal product
- Identifiable reporting source

- An event or outcome that can be identified as SAE
- Preliminary causality assessment
- Severity

All SAEs are also to be documented on the Adverse Events eCRF. Any medication or other therapeutic measures used to treat the AE will be recorded on the appropriate eCRF pages in addition to the grading and outcome of the AE.

Contact Persons and Numbers

The details of the Sponsor's contact person for safety reporting or questions are listed below and will also be kept on-site in the Investigator File.

[redacted]

[redacted]

[redacted]

Follow-up of SAEs

After receipt of the initial report, sponsor/designee may contact the investigator if it is necessary to obtain further information for assessment of the event.

All SAEs must be documented and followed up until the event has resolved, subsided, stabilized, or is otherwise explained. All follow-up activities have to be reported, if necessary on one or more consecutive SAE report forms in a timely manner.

9.12 TREATMENT OF AE AND SAES

Treatment of any AE and SAE is at the sole discretion of the investigator and according to current Good Medical Practice. The applied measures should be recorded in eCRF.
Cost of the medical care for vaccine related AEs will be borne by the sponsor.

10. STATISTICAL CONSIDERATIONS

10.1 OVERVIEW AND GENERAL CONSIDERATIONS

This is a Phase 3, observer-blind, randomised, active controlled study in adults aged ≥ 18 years in India who have already received primary vaccination against COVID-19 at least 6 months ago (6 months / 180 days from the second dose), to evaluate the immunogenicity and safety of the COVOVAX booster dose in comparison with the control vaccine (COVISHIELD and COVAXIN, for COVISHIELD and COVAXIN Prime cohorts, respectively).
A detailed statistical analysis plan will be created and finalized prior to database lock. All statistical analyses will be performed using SAS® software Version 9.4 or later. Additional details for statistical analysis will be included in the SAP.
Medical History and AEs will be coded using the Medical Dictionary for Regulatory Activities (MedDRA) (version 23.0 or later). The frequency count and percentage of participants will be summarized by system organ class (SOC) and preferred term (PT). Study participant-wise data listing will be provided.
Non-inferiority of COVOVAX booster over an active control vaccine group for each cohort will be concluded if both the lower limit of the two-sided 95% CI for the GMT ratio (GMR) >0.67 and the lower limit of the two-sided 95% CI for the difference in proportions of participants with ≥2-fold rise is >-10% for both anti-S IgG and nAbs between COVOVAX and the Control vaccine groups at Day 29. For consistency two-sided 95% confidence intervals (CIs) will be provided throughout. The main purpose of the safety analysis is to estimate the incidence rate of different events in each vaccine group.
No statistical tests will be performed at any interim analyses of safety data.

10.2 RANDOMIZATION

The randomization scheme for treatment assignment (vaccine groups) will be generated and maintained by independent personnel at PPD. PPD Biostatistics will generate the randomization schedule using SAS software Version 9.4 or later (SAS Institute Inc, Cary, North Carolina) for Interactive Response Technology (IRT), which will link sequential participant randomization numbers to treatment codes.

The eligible participants will be enrolled and randomized in the study online through IRT. Each participant enrolled into the study will be assigned a randomization number to assign vaccine group after identification and eligibility data have been entered into the IRT system.

A total of 372 eligible participants of ≥ 18 years of age who have completed primary 2 dose schedule of COVID-19 vaccination at least 6 months ago will be enrolled in the study in 2 cohorts of 186 participants each with 1:1 allocation to COVOVAX or control vaccine. All eligible participants (n=372) will receive 0.5 ml of either COVOVAX or the Control vaccine (COVISHIELD or COVAXIN in the respective cohort) on Day 1 as per randomization.

10.3 MULTIPLICITY

This trial has two separate cohorts with their own type I error family. There are four co-primary endpoints in each study cohort: GMR and difference in proportions of participants with at least 2-fold increase measured in anti-S IgG and nAb between COVOVAX and the active control vaccine at Day 29 after booster vaccination. To claim success of the primary objective in a cohort, all four null hypotheses of non-inferiority are required to be rejected at one-sided alpha level of 0.025 for each of them. No additional multiplicity adjustment will be performed.

10.4 SAMPLE SIZE AND POWER

It is planned to randomize 372 participants in the study with 186 in each of 2 cohorts who have received primary vaccination with either COVISHIELD OR COVAXIN. Assuming that the proportion of non-evaluable participants ≤ 15% (which leads to a sample size of 158 evaluable participants for each cohort), group sample sizes of 93 each in the tested vaccine group and the active control group achieve 80% power to detect non-inferiority using a one-sided, two-sample t-test. The margin of non-inferiority is -0.33. The true ratio of the means at which the power is evaluated is 1.00. The one-sided significance level (alpha) of the test is 0.025. The

coefficients of variation of both groups are assumed to be 1.1. Non-inferiority of COVOVAX booster over an active control vaccine group for each cohort will be concluded separately if the lower limit of the two-sided 95% CI for the GMT ratio for both anti-S IgG antibodies and neutralizing antibodies between COVOVAX and the active control vaccine at Day 29 is > 0.67 (non-inferiority margin).

With the same sample size and randomization ratio, the study will achieve 82% power to detect non-inferiority using a one-sided, two-sample z-test for difference in two proportions of participants achieving ≥ 2-fold increase. The margin of non-inferiority is -10%. The true difference of the proportion at which the power is evaluated is 0 and the proportion in the active control group is assumed 95%. The one-sided significance level (alpha) of the test is 0.025.

Sample size calculations were performed using a non-inferiority test for the ratio of two means and difference in two proportions in PASS 15.0.7 Version software.

The following table shows the evaluable sample size to demonstrate noninferiority of immune response:

Cohort	Power		Evaluable sample size (SS)			% Non-evaluable participants (Dropout rate)
	GMR	Diff. of Prop. of participants with ≥2-fold rise	Total number of participants	COVOVAX	Control (Covishield/ Covaxin for respective cohort)	
COVISHIELD Prime cohort	80	82	158	79	79	15%
COVAXIN Prime cohort	80	82	158	79	79	15%

Based on sample size of each arm, the 95% confidence interval for safety events are:

True safety event rate	95% Exact binomial CI				
	COVOVAX (N=93)	Active Control (N=93)	One Cohort (either COVISHIELD Prime or COVAXIN Prime) (N=186)	COVOVAX Booster Combined (N=186)	Overall (N=372)
1.0%	0% -5.7%	0% -5.7%	0.1% - 3.7%	0.1% - 3.7%	0.3% - 2.6%
2.0%	0.2% – 7.3%	0.2% – 7.3%	0.5% – 5.2%	0.5% – 5.2%	0.8% - 4.0%
5.0%	1.6% – 11.6%	1.6% – 11.6%	2.3% - 9.2%	2.3% - 9.2%	3.0% - 7.7%
10.0%	4.7% – 18.0%	4.7% – 18.0%	6.1% - 15.2%	6.1% - 15.2%	7.1% - 13.5%

10.5 ANALYSIS POPULATIONS

10.5.1 Enrolled Population

All participants who provide written informed consent, regardless of the participants screening, randomization and treatment status in the study.

10.5.2 Randomized Population

All participants in the enrolled population who are randomized (i.e. assigned treatment).

10.5.3 Full Analysis Population

All participants in the Enrolled population who received the study vaccine and provided an evaluable serum sample post vaccination for at least one assessment. Participants in the Full Analysis population will be analyzed ‘as randomized’, i.e., according to the vaccine a participant was designated to receive, which may be different from the vaccine the participants actually received.

10.5.4 Safety Population

All participants who receive the study vaccine [COVOVAX or Control Vaccine (COVISHIELD or COVAXIN)]. All safety analyses will be performed using this population. Participants in the safety population will be analyzed as ‘treated’ (i.e. actual vaccine received).

10.5.5 Per Protocol Population

Per Protocol population will be a subset of Full Analysis population. Per Protocol population consist of all participants who received the study vaccine and provided an evaluable serum sample post vaccination for at least one assessment and have baseline (Day 1) data available, excluding any data from time points following a SARS-CoV-2 infection or major protocol deviation (defined as use of an immunosuppressant, immune-modulating medication or vaccines which interfere with assessing immunogenicity), which means subject exclusion when any of these event(s) occurs before Day 29. All immunogenicity analyses will be performed using this population. Participants in the Per Protocol population will be analyzed ‘as randomized’ (i.e. planned vaccine group). The review and determination for exclusion from the Per Protocol Population will be carried out in a blinded fashion by a study clinician prior to unblinding.

10.6 ANALYSIS PLAN

10.6.1 Intercurrent events (IcEv)

Label	Intercurrent Event Type
IcEv1 (Death)	Death due to any cause; this is an IcEv because it leads to the endpoint (e.g. antibody titer) not existing at later timepoints.
IcEv2 (Immune modifiers)	Use of Immunosuppressant and Immune modifying medications or vaccines which interfere with assessing immunogenicity.
IcEv3 (COVID-19/SARS-CoV-2 infection)	Incidence of COVID-19/SARS-CoV-2 infection after vaccination.

10.6.2 Estimand Specifications

Attributes for the primary immunogenicity estimand with strategies for IcEvs are presented in the Table 11.5.2.1.

Table 11.5.2.1 Co-primary Objective(s) and Estimand(s) with Rationale for Strategies to Address Intercurrent Events

**Objective**	To compare immunogenicity of the COVOVAX booster dose in comparison with the Control vaccine by both anti-S IgG and nAbs for COVISHIELD Prime, and COVAXIN Prime cohorts, separately
**Estimand Label**	Estimand 1.1a and Estimand 1.2a
**Estimand Description**	Ratio of geometric mean titers (GMTs) of anti-S IgG and nAbs 28 days after vaccination (at Day 29) between vaccines (COVOVAX booster/ Active control).<br><br>Principal stratum and Hypothetical strategy are used to understand antibody levels achieved through vaccination, without subsequent COVID-19/SARS-CoV-2 infection or use of any immune-modifying medications or other vaccines.<br>While on treatment strategy is used for event death. (data until death is utilized)
**Target Population**	Vaccinated individuals aged 18 years and older
**Endpoint**	Anti-S IgG and nAbs at Day 29
**Treatment Condition(s)**	Test: COVOVAX and<br>Reference: Active Control vaccine, COVISHIELD or COVAXIN
**Population**<br>**Level Summary**	Ratio of GMTs between vaccine groups (test/reference)

**Intercurrent Event Strategy**	
**IcEv1 (Death)**<br>**IcEv2(Immune modifiers)**<br>**IcEv3(COVID-19/SARS-CoV-2 infection)**	While on Treatment strategy<br>Principal stratum and Hypothetical strategy<br>Principal stratum and Hypothetical strategy
**Rationale for**<br>**Strategies**	The hypothetical strategy is used to estimate effect attributable to the difference in vaccines without any use of immune-modifying medications or other vaccinations and without influence from subsequent COVID-19/SARS-CoV-2 infection and while on treatment strategy for death. Principal stratum will be applicable when any of these intercurrent events occur before Day 29.

Refer to Section 11.5.1 specific numbered intercurrent event definitions.

Table 11.5.2.1a Co-primary Objective(s) and Estimand(s) with Rationale for Strategies to Address Intercurrent Events

**Objective**	To compare immunogenicity of the COVOVAX booster dose in comparison with the Control vaccine by both anti-S IgG and nAbs for COVISHIELD Prime, and COVAXIN Prime cohorts, separately
**Estimand Label**	Estimand 1.1b and Estimand 1.2b
**Estimand Description**	Proportion of participants achieving ≥ 2-fold increase in anti-S IgG and nAbs 28 days after vaccination (at Day 29) between vaccines (COVOVAX booster/ Active control).<br><br>Principal stratum and Hypothetical strategy are used to understand antibody levels achieved through vaccination, without subsequent COVID-19/SARS-CoV-2 infection or use of any immune-modifying medications or other vaccines.<br>While on treatment strategy is used for event death. (data until death is utilized)
**Target Population**	Vaccinated individuals aged 18 years and older
**Endpoint**	Proportion of participants achieving ≥ 2-fold increase in anti-S IgG and nAbs at 28 days after booster vaccination
**Treatment Condition(s)**	Test: COVOVAX and<br>Reference: Active Control vaccine, COVISHIELD or COVAXIN
**Population**<br>**Level Summary**	Difference in proportions of participants with ≥ 2-fold increase in anti-S IgG and nAbs at 28 days after in vaccine groups(test/reference)
**Intercurrent Event Strategy**<br>**IcEv1 (Death)**<br>**IcEv2(Immune modifiers)**<br>**IcEv3(COVID-19/SARS-CoV-2 infection)**	 While on Treatment strategy<br>Principal stratum and Hypothetical strategy<br>Principal stratum and Hypothetical strategy
**Rationale for**<br>**Strategies**	The hypothetical strategy is used to estimate effect attributable to the difference in vaccines without any use of immune-modifying medications or other vaccinations and without influence from subsequent COVID-19/SARS-CoV-2 infection and while on treatment strategy for death. Principal stratum will be applicable when any of these intercurrent events occur before Day 29.

Table 11.5.2.2 Secondary Safety Objective (s) and Estimand(s) with Rationale for Strategies to Address Intercurrent Events

**Objective**	To assess the tolerability and reactogenicity profile of COVOVAX in comparison with the Control vaccine for COVISHIELD Prime, and COVAXIN Prime cohorts, separately
**Estimand Label**	Estimand 2, Estimand 3
**Estimand Description**	Proportion of participants with at least one occurrence of<br>1. Solicited local and/or systemic adverse event (AE) up to 7 days post-vaccination<br>2. Unsolicited AE up to 28 days post-vaccination<br>3. SAE throughout the study duration following vaccination<br>4. Adverse event of special interest (AESI) throughout the study duration following vaccination<br>A treatment policy strategy is used for assessing safety irrespective of use of immune-modifying medications/vaccinations.<br>An intercurrent event death will use strategy as follows:<br>• Composite strategy is used for event death in case of SAE<br>• Unsolicited AE, adverse event of special interests, solicited local adverse events and/or systemic adverse events will use while on treatment strategy (i.e. data until death is utilized)<br>An intercurrent event COVID-19/SARS-CoV-2 infection will use Treatment policy for all listed above.
**Target Population**	Vaccinated individuals aged 18 years and older.
**Endpoint**	1. Occurrence of solicited local and/or systemic adverse events (AEs) for 7 days post-vaccination<br>2. Occurrence of unsolicited AEs for 28 days post-vaccination<br>3. Occurrence of SAEs, and adverse event of special interest (AESI) throughout the study duration following vaccination
**Treatment Condition(s)**	COVOVAX and the Control vaccine (COVISHIELD or COVAXIN) from COVISHIELD and COVAXIN prime cohorts, separately
**Population**<br>**Level Summary**	Proportion
**Intercurrent Event Strategy**	
**IcEv1 (Death)**	Composite strategy is used for SAE and while on treatment is used for other safety endpoints.
**IcEv2(Immune modifiers)**	Treatment policy for all endpoints
**IcEv3(COVID-19/SARS-CoV-2 infection)**	Treatment policy for all endpoints

**Rationale for Strategies**	A treatment policy strategy is used for assessing safety irrespective of use of Immune modifiers (we cannot exclude the safety events even subject receive the immune modifier).<br>Death will use composite strategy/ while on treatment based on AEs under consideration. SAE will use composite strategy as death is serious adverse event.<br>COVID-19/SARS-CoV-2 infection will use treatment policy to assess safety based on endpoint under consideration as assessing tolerability and reactogenicity irrespective of COVID-19/SARS-CoV-2 infection.

Refer to Section 11.5.1 specific numbered intercurrent event definitions.

Table 11.5.2.3 Secondary Immunogenicity Objective (s) and Estimand(s) with Rationale for Strategies to Address Intercurrent Events

**Objective**	To assess immunogenicity of the COVOVAX booster dose in comparison with the Control vaccine by anti-S IgG and neutralizing antibody assays for COVISHIELD Prime, and COVAXIN Prime cohorts, separately
**Estimand Label**	Estimand 4 and Estimand 5
**Estimand Description**	GMTs and GMFR of anti-S IgG antibodies at 28, 90 and 180 days post-vaccination.<br>GMTs and GMFR of nAbs at 28 and 180 days post-vaccination.<br>Principal stratum and Hypothetical strategy is used to understand antibody levels achieved through vaccination, without subsequent COVID-19/SARS-CoV-2<br>infection or use of any immune-modifying medications or other vaccines.<br>While on treatment strategy is used for event death.
**Target Population**	Vaccinated individuals aged 18 years and older.
**Endpoint**	Anti-S IgG antibodies at Day 29, Day 91 and Day 181<br>nAbs at Day 29 and Day 181
**Treatment Condition(s)**	COVOVAX and the Control vaccine (COVISHIELD or COVAXIN)
**Population**<br>**Level Summary**	GMTs and GMFR for each vaccine (test/reference)
**Intercurrent Event Strategy**	
**IcEv1 (Death)**<br>**IcEv2(Immune modifiers)**<br>**IcEv3(COVID-19/SARS-CoV-2 infection)**	While on treatment strategy<br>Principal stratum and Hypothetical strategy<br>Principal stratum and Hypothetical strategy
**Rationale for**<br>**Strategies**	The hypothetical strategy is used to antibody levels achieved through vaccination without subsequent COVID-19/SARS-CoV-2 infection or use of any immune-modifying medications or other vaccines. Principal stratum will be applicable when any of these intercurrent events occur before Day 29.<br><br>While on treatment policy is used to utilize the data until death

Refer to Section 11.5.1 specific numbered intercurrent event definitions.

Table 11.5.2.4 Secondary Immunogenicity Objective (s) and Estimand(s) with Rationale for Strategies to Address Intercurrent Events

**Objective**	To assess immune response of COVOVAX booster dose between COVISHIELD Prime, and COVAXIN Prime cohorts by anti-S IgG and neutralizing antibody assays
**Estimand Label**	Estimand 6 and Estimand 7
**Estimand Description**	GMTs of anti-S IgG antibodies of COVOVAX from COVISHIELD prime and COVOVAX from COVAXIN prime at 28, 90 and 180 days after vaccination<br>GMTs of nAbs of COVOVAX from COVISHIELD prime and COVOVAX from COVAXIN prime at 28 and 180 days after vaccination.<br>Principal stratum and Hypothetical strategy are used to understand antibody levels achieved through vaccination, without subsequent COVID-19/SARS-CoV-2 infection or use of any immune-modifying medications or other vaccines.<br>While on treatment strategy is used for event death.
**Target Population**	Vaccinated individuals aged 18 years and older.
**Endpoint**	Anti-S IgG antibodies at Day 29, Day 91 and 181<br>nAbs at Day 29 and Day 181
**Treatment Condition(s)**	COVOVAX Booster from COVISHIELD Prime cohort and<br>COVOVAX Booster from COVAXIN Prime cohort
**Population Level Summary**	GMTs for each vaccine (COVOVAX Booster from COVISHIELD prime/ COVOVAX Booster from COVAXIN prime cohorts)
**Intercurrent Event Strategy**	
**IcEv1 (Death)**	While on treatment strategy
**IcEv2(Immune modifiers)**	Principal stratum and Hypothetical strategy
**IcEv3(COVID-19/SARS-CoV-2 infection)**	Principal stratum and Hypothetical strategy
**Rationale for Strategies**	The hypothetical strategy is used to antibody levels achieved through vaccination without subsequent COVID-19/SARS-CoV-2 infection or use of any immune-modifying medications or other vaccines. Principal stratum will be applicable when any of these intercurrent events occur before Day 29.<br>While on treatment policy is used to utilize the data until death

Refer to Section 11.5.1 specific numbered intercurrent event definitions.

Table 11.5.2.5 Secondary Immunogenicity Objective (s) and Estimand(s) with Rationale for Strategies to Address Intercurrent Events

**Objective**	To assess immune response of Covishield booster dose in COVISHIELD Prime and Covaxin booster dose in COVAXIN Prime cohorts by anti-S IgG and neutralizing antibody assays
**Estimand Label**	Estimand 8 and Estimand 9
**Estimand Description**	GMTs of anti-S IgG antibodies at 28, 90 and 180 days after vaccination<br>GMTs of nAbs at 28 and 180 days after vaccination.<br><br>Principal stratum and Hypothetical strategy are used to understand antibody levels achieved through vaccination, without subsequent COVID-19/SARS-CoV-2 infection or use of any immune-modifying medications or other vaccines.<br>While on treatment strategy is used for event death.
**Target Population**	Vaccinated individuals aged 18 years and older.
**Endpoint**	Anti-S IgG antibodies at Day 29, Day 91 and 181<br>nAbs at Day 29 and Day 181
**Treatment Condition(s)**	COVISHIELD Booster from COVISHIELD Prime cohort and<br>COVAXIN Booster from COVAXIN Prime cohort
**Population Level Summary**	GMTs for each vaccine COVISHIELD Booster from COVISHIELD prime/ COVAXIN Booster from COVAXIN prime cohorts
**Intercurrent Event Strategy**	
**IcEv1 (Death)**<br>**IcEv2(Immune modifiers)**<br>**IcEv3(COVID-19/SARS-CoV-2 infection)**	While on treatment strategy<br>Principal stratum and Hypothetical strategy<br>Principal stratum and Hypothetical strategy
**Rationale for Strategies**	The hypothetical strategy is used to antibody levels achieved through vaccination without subsequent COVID-19/SARS-CoV-2 infection or use of any immune-modifying medications or other vaccines. Principal stratum will be applicable when any of these intercurrent events occur before Day 29.<br>While on treatment policy is used to utilize the data until death

Refer to Section 11.5.1 specific numbered intercurrent event definitions.

Table 11.5.2.6 Exploratory Immunogenicity Objective (s) and Estimand(s) with Rationale for Strategies to Address Intercurrent Events

**Objective**	To assess cell mediated immune response of the COVOVAX in comparison with the Control vaccine for COVISHIELD Prime, and COVAXIN Prime cohorts, separately
**Estimand Label**	Estimand 10
**Estimand Description**	The mean change from baseline in cell-mediated immune responses as measured by enzyme-linked immune absorbent spot (ELISpot) at 28 days and 180 days post-vaccination<br>Principal Stratum and Hypothetical strategy are used to understand antibody levels achieved through vaccination, without subsequent COVID-19/SARS-CoV-2 infection or use of any immune-modifying medications or other vaccines.<br>While on treatment strategy is used for event death.
**Target Population**	Vaccinated individuals aged 18 years and older.
**Endpoint**	• Change from Baseline (Day 1) in the cell-mediated immune responses at Day 29 and Day 181.
**Treatment Condition(s)**	COVOVAX and the Control vaccine (COVISHIELD or COVAXIN)
**Population Level Summary**	• Mean change from baseline in cell-mediated immune responses for COVOVAX and Control vaccine (COVISHIELD or COVAXIN)
**Intercurrent Event Strategy**	
**IcEv1 (Death)**<br>**IcEv2(Immune modifiers)**<br>**IcEv3(COVID-19/SARS-CoV-2 infection)**	While on treatment strategy<br>Principal stratum and Hypothetical strategy<br>Principal stratum and Hypothetical strategy
**Rationale for Strategies**	Principal stratum and Hypothetical strategy is used to understand cell mediated response achieved through vaccination, without subsequent COVID-19/SARS-CoV-2 infection or use of any immune-modifying medications or other vaccines. Principal stratum will be applicable when any of these intercurrent events occur before Day 29.<br>While on treatment policy is used to utilize the data until death

Refer to Section 11.5.1 specific numbered intercurrent event definitions.

Table 11.5.2.7 Exploratory Immunogenicity Objective (s) and Estimand(s) with Rationale for Strategies to Address Intercurrent Events

**Objective**	To assess immune response by neutralizing antibodies against variants of concern for COVISHIELD Prime, and COVAXIN Prime cohorts, separately
**Estimand Label**	Estimand 11
**Estimand Description**	GMTs and GMFR of neutralizing antibodies (nAbs) against variants of concern at 28 and 180 days post-vaccination.<br>Principal Stratum and Hypothetical strategy are used to understand antibody levels achieved through vaccination, without subsequent COVID-19/SARS-CoV-2 infection or use of any immune-modifying medications or other vaccines.<br>While on treatment strategy is used for event death.
**Target Population**	Vaccinated individuals aged 18 years and older.
**Endpoint**	nAbs at Day 29 and Day 181 against variants of concern.
**Treatment Condition(s)**	COVOVAX and the Control vaccine (COVISHIELD or COVAXIN)
**Population Level Summary**	GMTs and GMFRs of nAbs against variants of concern
**Intercurrent Event Strategy**	
**IcEv1 (Death)**<br>**IcEv2(Immune modifiers)**<br>**IcEv3(COVID-19/SARS-CoV-2 infection)**	While on treatment strategy<br>Principal Stratum and Hypothetical strategy<br>Principal Stratum and Hypothetical strategy
**Rationale for Strategies**	Principal Stratum and Hypothetical strategy is used to understand cell mediated response achieved through vaccination, without subsequent COVID-19/SARS-CoV-2 infection or use of any immune-modifying medications or other vaccines. Principal stratum will be applicable when any of these intercurrent events occur before Day 29.<br>While on treatment strategy is used for event death.

10.6.3 Table of Statistical Method and Sensitivity Analysis

		Main Estimation			Sensitivity Analysis
**Estimand Label**	**Estimand Description**	**Analysis Set**	**Imputation/ Data/ Censoring Rules**	**Analysis Model/Method**	
Estimand 1.1a, Estimand 1.2a (Co-Primary)	Geometric Mean ratio (GMR) of anti-S IgG at 28 days after vaccination between vaccines (COVOVAX booster/ control) for each cohort<br><br>Principal Stratum is used to understand antibody levels achieved through vaccination, without subsequent COVID-19/SARS-CoV-2 infection or use of any immune-modifying medications or other vaccines.<br><br>While on treatment strategy is used for event death.	Per protocol Population	Values below the limit of quantification (LOQ) or limit of detection (LOD) will be replaced by LOQ/2 and LOD/2, respectively before log transformation.<br>Multiple imputation of missing values assumed to MAR.	ANCOVA will be fitted to the log transformed anti-S IgG or nAbs with terms for vaccine group, log baseline titer, age, sex, duration between 1st - 2nd vaccine dose, duration between 2nd dose to booster dose of vaccine.<br>Individual mean and 95% CI values by treatment from this model will be used to generate the geometric mean titers (GMT) at each time point and geometric mean ratio (GMR) with 95% CI at Day 29 after vaccination by back transforming to the original scale.<br><br>This analysis will be performed for each type antibody (anti-S IgG or nAbs) and cohort separately.<br><br>Hypothesis testing:<br>H_0: GMTT/GMTC ≤ 0.67<br>H_1: GMTT/GMTC > 0.67<br>Where, T =COVOVAX and C=Active Control vaccine (COVISHIELD or COVAXIN).<br>The lower limit of the 95% CI for the GMR will be compared with a non-inferiority margin of 0.67 and COVOVAX vaccine will be declared non-inferior to the Control vaccine (COVISHIELD or COVAXIN) if > 0.67 on both anti-S IgG antibody and nAbs.	Supplementary: Similar repeat analysis based on Full Analysis population will be provided.<br><br>Hypothetical is used to understand antibody levels achieved through vaccination, without subsequent COVID-19/SARS-CoV-2 infection or use of any immune-modifying medications or other vaccines.

		Main Estimation			Sensitivity Analysis
**Estimand Label**	**Estimand Description**	**Analysis Set**	**Imputation/ Data/ Censoring Rules**	**Analysis Model/Method**	
Estimand 1.1b, Estimand 1.2b (Co-Primary)	Difference in proportions of participants achieving ≥ 2-fold increase in anti-S IgG and nAbs 28 days after vaccination (at Day 29) between vaccines (COVOVAX booster/ Active control).<br><br>Principal stratum and Hypothetical strategy are used to understand antibody levels achieved through vaccination, without subsequent COVID-19/SARS-CoV-2 infection or use of any immune-modifying medications or other vaccines.<br>While on treatment strategy is used for event death. (data until death is utilized)	Per protocol Population	Values below the limit of quantification (LOQ) or limit of detection (LOD) will be replaced by LOQ/2 and LOD/2, respectively before log transformation.	The difference between the vaccines (COVOVAX booster - Active control) in the proportion of the participants achieving ≥ 2-fold increase in anti-S IgG nAbs 28 days after vaccination will be provided along with their two-sided 95% CIs obtained by the Miettinen and Nurminen method<br><br>This analysis will be performed for each type antibody (anti-S IgG or nAbs) and cohort separately.<br><br>H_0: P_T-$P_C \leq$ -10% versus<br>H_1: P_T-$P_C >$ -10%<br><br>Where, T =COVOVAX and C=Active Control vaccine (COVISHIELD or COVAXIN).<br>The lower limit of the 95% CI for the for difference in proportion will be compared with a non-inferiority margin of -10% and COVOVAX vaccine will be declared non-inferior to the Control vaccine (COVISHIELD or COVAXIN) if > -10% on both anti-S IgG antibody and nAbs.	Additional details for the sensitivity analysis will be included in the SAP.
Estimand 2 Estimand 3 (Secondary)	Proportion of participants with at least one occurrence of<br>1. solicited local and/ or systemic adverse event (AEs) for 7 days post-vaccination,<br>2. unsolicited AEs for 28 days post-vaccination and<br>3. SAEs throughout the study	Safety Population	None	Frequencies and estimate of the proportion of participants with at least one solicited local and systemic adverse event (AE) for 7 days post-vaccination, unsolicited AE for 28 days post-vaccination, SAE, and adverse events of special interest (AESI) throughout the study duration following vaccination will be computed by vaccine	

		Main Estimation			Sensitivity Analysis
**Estimand Label**	**Estimand Description**	**Analysis Set**	**Imputation/ Data/ Censoring Rules**	**Analysis Model/Method**	
	duration following vaccination<br>4. Adverse events of special interest (AESI) throughout the study duration following vaccination<br><br>A treatment policy strategy is used for assessing safety irrespective of use of immune-modifying medications/vaccinations.<br>An intercurrent event death will use strategy as follows:<br>• Composite strategy is used for event death in case of SAE<br>• Solicited local and systemic adverse events, unsolicited adverse events and AESI will use while on treatment strategy (i.e. data until death is utilized)<br><br>An intercurrent event COVID-19/SARS-CoV-2 infection will use<br>Treatment policy for all endpoints listed above.			group using two sided 95% Clopper-Pearson confidence intervals.<br><br>This analysis will be performed for each cohort separately.	
Estimand 4, Estimand 5 (Secondary)	GMTs and GMFR of anti-S IgG antibodies at 28, 90 and 180 days post-vaccination.<br><br>GMTs and GMFR of nAbs at 28 and 180 days post-vaccination.	Per protocol Population	Values below the limit of quantification (LOQ) or limit of detection (LOD) will be replaced by LOQ/2 and LOD/2, respectively	MMRM will be fitted to the log transformed anti-S IgG and nAb with terms for vaccine group, visit, log baseline titer, age, sex, duration between 1st - 2nd vaccine dose, duration between 2nd dose to booster dose of vaccine with interactions	

			Main Estimation		Sensitivity Analysis
**Estimand Label**	**Estimand Description**	**Analysis Set**	**Imputation/ Data/ Censoring Rules**	**Analysis Model/Method**	
	Principal Stratum and Hypothetical strategy are used to understand antibody levels achieved through vaccination, without subsequent COVID-19/SARS-CoV-2 infection or use of any immune-modifying medications or other vaccines.<br><br>While on treatment strategy is used for event death.			for treatment by visit. The repeated timepoints on subject will be modelled (Details of the covariance structure will be provided in the SAP).<br><br>Individual mean and 95% CI values by treatment from this model will be used to generate the geometric mean titers (GMTs) for anti-S IgG and nAb with at each time point by back transforming to the original scale.<br><br>GMFRs will be provided descriptively for:<br>anti-S IgG antibodies at 28, 90 and 180 days post-vaccination,<br>nAb at 28 and 180 days post-vaccination.<br><br>This analysis will be performed for each cohort separately.	
Estimand 6 and Estimand 7 (Secondary)	GMTs of anti-S IgG antibodies at 28, 90 and 180 days after vaccination<br>GMTs of nAbs at 28 and 180 days after vaccination.<br><br>Principal Stratum and Hypothetical strategy are used to understand antibody levels achieved through vaccination, without subsequent COVID-19/SARS-CoV-2 infection or use of any immune-modifying medications or other vaccines.<br>While on treatment strategy is	Per protocol Population	Values below the limit of quantification (LOQ) or limit of detection (LOD) will be replaced by LOQ/2 and LOD/2, respectively	For this analysis, data from COVOVAX booster from each cohort will be used.<br><br>GMTs for COVOVAX from COVISHIELD prime and COVOVAX from COVAXIN prime will be reported descriptively along with 95% CI.<br><br>The 95% CI will be calculated based on the t-distribution of the log transformed values for geometric means then back transformed to the original<br>scale for presentation.<br><br>Analysis method to account for possible	

			Main Estimation		Sensitivity Analysis
**Estimand Label**	**Estimand Description**	**Analysis Set**	**Imputation/ Data/ Censoring Rules**	**Analysis Model/Method**	
	used for event death.			covariates may be employed and details will be provided in the SAP.	
Estimand 8 and Estimand 9 (Secondary)	GMTs of anti-S IgG antibodies at 28, 90 and 180 days after vaccination<br><br>GMTs of nAbs at 28 and 180 days after vaccination.<br><br>Principal Stratum and Hypothetical strategy are used to understand antibody levels achieved through vaccination, without subsequent COVID-19/SARS-CoV-2 infection or use of any immune-modifying medications or other vaccines.<br>While on treatment strategy is used for event death.	Per protocol Population	Values below the limit of quantification (LOQ) or limit of detection (LOD) will be replaced by LOQ/2 and LOD/2, respectively	For this analysis, data for COVISHILED booster dose from COVISHIELD Prime and COVAXIN booster dose from COVAXIN Prime cohorts will be used.<br><br>GMTs for COVISHIELD booster group from COVISHIELD prime and COVAXIN booster group from COVAXIN prime will be reported descriptively along with 95% CI.<br><br>The 95% CI will be calculated based on the t-distribution of the log transformed values for geometric means then back transformed to the original<br>scale for presentation.<br><br>Analysis method to account for possible covariates may be employed and details will be provided in the SAP.	
Estimand 10 (Exploratory)	The mean change from baseline in cell-mediated immune responses as measured by enzyme-linked immune absorbent spot (ELISpot) at 28 days and 180 days post-vaccination<br>Principal Stratum and Hypothetical strategy are used to understand cell-mediated immunity achieved through vaccination, without subsequent COVID-19/SARS-CoV-2	Per protocol Population		ANCOVA will be fitted to change from Baseline in cell-mediated immune responses with terms for vaccine group, Baseline cell-medicated immune response value, age and sex, duration between 1st - 2nd vaccine, and duration between 2nd to booster.<br><br>Mean difference between treatment groups and associated 95% CI will be presented.	

		Main Estimation			Sensitivity Analysis
**Estimand Label**	**Estimand Description**	**Analysis Set**	**Imputation/ Data/ Censoring Rules**	**Analysis Model/Method**	
	infection or use of any immune-modifying medications or other vaccines.<br>While on treatment strategy is used for event death.			This analysis will be performed each cohort separately.	
Estimand 11 (Exploratory)	GMTs and GMFR of nAbs against variants of concern at 28 and 180 days post-vaccination.<br><br>Principal Stratum and Hypothetical strategy are used to understand cell-mediated immunity achieved through vaccination, without subsequent COVID-19/SARS-CoV-2 infection or use of any immune-modifying medications or other vaccines.<br>While on treatment strategy is used for event death.	Per protocol Population	Values below the limit of quantification (LOQ) or limit of detection (LOD) will be replaced by LOQ/2 and LOD/2, respectively	GMTs and GMFRs will be provided descriptively for:<br><br>nAbs at 28- and 180-days post-vaccination<br><br>The 95% CI will be calculated based on the t-distribution of the log transformed values for geometric means or GMFRs, then back transformed to the original scale for presentation.	

The details about analysis regarding cell mediated immune responses will be defined in the SAP.

An interim analysis is planned for the safety and immunogenicity data by anti- S IgG at 28 days post study vaccination (Day 29).

10.6.4 Analysis of Demographic and Baseline Characteristics

Demographic (age, gender, height, weight) and baseline characteristics (medical history, Pre-existing conditions, and Prior medications) will be presented descriptively by vaccine group.

The quantitative variables will be summarized as mean, standard deviation, median, minimum and maximum and categorical variables will be summarized as frequency and percentage. Distributions of participants by gender, age will be summarized as frequency and percentages by cohort and within each cohort as overall and by vaccine group.

Baseline characteristics such as medical history, pre-existing conditions will be tabulated by vaccine group within each cohort using MedDRA dictionary classification and prior and concomitant medications will be tabulated by vaccine group within each cohort separately using WHODD drug classification.

10.6.5 Statistical Methods for Co-Primary Objectives

Statistical Method for the Co-primary endpoints

A summary of the statistical methods for the co-primary objectives are presented in the section 11.5.3 of the protocol.

To assess the primary objectives, the following non-inferiority hypotheses will be tested on the GMT of anti-S IgG antibodies and nAbs at 28 days (Day 29) post study vaccination.

1) Hypothesis testing for GMR:

   H_0: $GMT_T/GMT_C \leq 0.67$ (Inferior)

   H_1: $GMT_T/GMT_C > 0.67$ (Non-inferior)

   Where – $_T$- COVOVAX and $_C$ -COVISHIELD or COVAXIN in the relevant cohort

The lower limit of the 95% CI will be compared with the non-inferiority margin 0.67 and COVOVAX will be declared non-inferior to the active control vaccine, COVISHIELD or COVAXIN in each cohort if > 0.67.

2) Hypothesis testing for difference of proportions of participants with ≥2-fold rise
H_0: P_T-$P_C \leq$ -10% versus
H_1: P_T-P_C > -10%

Where – $_T$- COVOVAX and $_C$ -COVISHIELD or COVAXIN in the relevant cohort.
The lower limit of the 95% CI for the difference in proportion will be compared with a non-inferiority margin of -10% and COVOVAX vaccine will be declared non-inferior to the Control vaccine (COVISHIELD or COVAXIN) if the lower limit > -10% on both anti-S IgG antibody and nAbs.

10.6.6 Statistical Methods for Secondary and Exploratory Objectives

Immunogenicity analysis of neutralizing antibodies (nAbs), anti-S IgG antibodies and cell-mediated immune responses**.**
A summary of the statistical methods and sensitivity analysis for the immunogenicity objective (Estimand 4, 5, 6, 7, 8 and 9) is presented in the section 11.5.3 of the protocol. Summary of statistical methods for cell-mediated immune response (Estimand 10) is presented in the section 11.5.3 of the protocol.
In addition to the proposed analysis in section 11.5.3, the GMTs and GMFRs from baseline will be summarized with descriptive statistics including a boxplot (on log scale) versus time for each cohort separately.

10.6.7 Safety Objectives

Analysis of Solicited and Unsolicited Adverse Events
A summary of the statistical methods for the analysis relating secondary objective of safety, tolerability and reactogenicity profile (Estimand 2 and 3) is presented in Section 11.5.3 of the protocol.
In addition to above proposed analysis, the following summaries will be provided.
All solicited AEs will be summarized according to defined severity grading scales. Frequencies and percentages of participants experiencing each AE will be presented for each symptom severity. Summary tables showing the occurrence of any local or systemic AE overall will also be presented.

Solicited local and systemic adverse events reported until 7 days post-vaccination will be summarized by maximal severity and by vaccine group within each cohort. All the solicited reactions occurring up to 7 days after each vaccination will be summarized similarly.

All unsolicited AEs reported until 28 days post-vaccination, assessed either as related or not related to vaccine by the investigator, will be recorded. The original verbatim terms used by investigators to identify AEs in the eCRFs will be mapped to PTs using MedDRA. The AEs will then be grouped by MedDRA PTs into frequency tables according to SOC. All reported AEs, as well as AEs assessed by the investigator as related to vaccine, will be summarized according to SOC, PT within SOC, and severity.

Safety and tolerability of study vaccines will be evaluated using the following endpoints:

- Number and severity of solicited local and systemic adverse events (AEs) and relatedness of all solicited systemic adverse events during the first 7 days after each vaccination.
- Number, severity and relatedness of all unsolicited AEs through 28 days after vaccination.
- Number, severity and relatedness of all SAEs through the entire study period.
- Number, severity and relatedness of all AESIs through the entire study period.

Generally, safety evaluations will be descriptive in nature. Tabular summaries of safety data will be provided for each vaccine group within each cohort.
Occurrence of local and systemic reactogenicity within 7 days after vaccination, as well as AEs through 28 days after vaccination and SAEs and AESIs during the entire study period, will be reported for all vaccine groups within each cohort.
Data listings of all adverse events will be provided by participant.
Additional details of the safety analysis such as (vital, physical examination. Etc.), disposition demographic will be provided in the statistical analysis plan.

10.6.8 Handling of Dropouts and Missing Data

Details for the imputation of missing values will be documented in the SAP based on details outlined in Section 11.5.3.

11. QUALITY CONTROL AND QUALITY ASSURANCE

11.1 PRE-STUDY DOCUMENTATION

Prior to enrolment of participants at the study site, specific regulatory documents must be available, such as regulatory (DCGI) and Institutional Ethics Committee (IECs) approvals; curriculum vitae for investigator and study staff; standard operating procedures (SOPs) and other essential documents. Sponsor/designee will inform the investigator which documents need to be provided according to the applicable regulatory requirements.

11.2 MONITORING

Sponsor monitoring responsibilities will be provided through qualified and appropriately trained individuals designated by CRO to carefully monitor all aspects of the study. A site initiation visit will be conducted prior to the beginning of the study and monitoring will be conducted during and at closeout of the study by the study monitor.

During the course of the study, the monitors will visit the clinical sites at intervals in order to verify that:

- The data are authentic, accurate and complete
- The safety and rights of participants are being protected
- The study is conducted in accordance with the approved protocol (and any subsequent amendment), GCP and all applicable regulatory requirements

Monitors will periodically contact the site and perform site visits. The extent, nature and frequency of site visits will be decided before the start of the study and will be based on considerations as study objectives, study design and complexity, and enrolment rate. During these contacts, the monitor will:

- Check and assess the progress of the study
- Review study data collected
- Perform source data verification, identify any issues and address their resolution

Monitoring will be conducted according to ICH-GCP. The individuals responsible for monitoring the study will have access to all records necessary to ensure the integrity/validity of the recorded data and will periodically review the progress of the study.

The investigator must agree to allow the monitor direct access to all relevant documents and to allocate his/her time and the time of his/her staff to the monitor to discuss findings and any relevant issues.

The monitor must contact the site prior to the start of the study to discuss the protocol and data collection procedures with the site personnel.

The investigator should allow representatives of the Ethics Committee, Regulatory Authority and the sponsor to visit the study site.

11.3 DATA MANAGEMENT AND PROCESSING

Site PI is responsible for ensuring timely completeness and accuracy of data reported. Data collection is the responsibility of clinical trial staff at the study site under supervision of site PI. The CRO is responsible for clinical data management activities, including quality review, analysis and reporting of study data according to SOPs.

11.3.1 Data Collection

Data will be entered electronically by site study staff using Internet in eCRF. The data system will include password protection. Instructions for use of the system will be included in eCRF manual.

Clinical data will be entered directly from source documents. All source documents should be completed in neat and legible manner to ensure accurate interpretation of data. All information required by the study protocol must be entered into eCRF. An explanation must be provided for any missing data. Source documentation supporting the eCRF data should document the dates and details of study procedures, AEs and participant status. PI/site staff will maintain information in eCRFs and all source documents that support the data collected from each participant.

Study monitor will check for completeness and accuracy of eCRF during the monitoring visits.

11.3.2 Data Management Procedures

Site staff should complete eCRFs as soon as possible after the information is collected. Completed eCRFs must be submitted for each screened participant who signs the study specific ICF.

Internal data quality checks such as automatic range checks, checks to identify data that appear inconsistent, incomplete or inaccurate will be programmed into eCRF that will help in real time review of data, as and when, clinical data is entered into the system by site staff.

Clinical Data Management team at CRO will review the data for quality and will provide several quality assurance reports to ensure that study data is clean and complete. Quality assurance reports will include, but are not limited to, the following: missing forms, automated and manual data queries. Data queries will be distributed to the sites at scheduled time period for site staff to review and update the database.

11.3.3 Coding

All medical verbatim terms will be coded by Clinical Data Management and reviewed by a medical doctor according to most recent versions of MedDRA (Adverse events and medical history) and the WHO Drug Dictionary enhanced version (concomitant medication).

11.3.4 Database Lock Procedures

Database will be locked upon completion of the following activities:

- All participants have completed the follow up visits
- All the participant data has been entered in the database
- All data anomalies have been resolved
- Study monitoring has been completed
- All listings of the database have been reviewed and discussed for assessment of consistency and medical plausibility.

11.3.5 Procedures for Analysis

Data will be analyzed as per the **pre-specified Statistical Analysis Plan (SAP)** after the database lock**.** An audit trail will be kept of any subsequent changes to the data.

11.4 STUDY AND SITE CLOSURE

Upon completion of the study, the monitor and the investigator will conduct the following activities:

- Data clarification and/or resolution

- Accounting, reconciliation and return to sponsor or destruction at sites of used and unused vaccines
- Review of site study records for completeness
- Return of all study data to Sponsors or designee.

Sponsors reserve the right to temporarily suspend or prematurely discontinue this study at either a single site or at all sites at any time for any other reason.

If the study is stopped or suspended prematurely, Sponsor will inform the investigator(s) as well as the regulatory authorities about the decision and the reasons for termination or suspension. If such action is taken, all effort must be made to ensure the safety of the participants enrolled in the study. The investigator(s) will inform the responsible IECs and provide the reason for the suspension or termination.

In case of premature study or study site closure, the monitor will conduct all activities as indicated above.

11.5 AUDITS AND INSPECTIONS

For the purpose of compliance with ICH-GCP and regulatory guidelines, it may be possible that the sponsor/designee or a national regulatory authority may conduct a site audit/inspection. This may occur at any time from start to after conclusion of the study.

The investigator agrees to allow the auditor direct access to all relevant documents and to allocate his/her time and the time of his/her staff to the auditor to discuss findings and any relevant issues.

If a regulatory authority requests an inspection, the investigator must inform the sponsor or its designee immediately about this request. The investigator(s) and the study coordinator(s) must make the relevant records available for inspection and must be available to respond to reasonable requests and audit queries made by authorized representatives of regulatory agencies. The sponsor will provide any needed assistance in responding to regulatory audits or correspondence.

12. REGULATORY AND ETHICAL REQUIREMENTS

12.1 ETHICS COMMITTEE REVIEW AND COMMUNICATION

It is the investigator's responsibility to ensure that this protocol is reviewed and approved by the IECs responsible for the study sites. The IECs must also review and approve the Informed Consent Form and any other written information to be provided to the participant. Written IEC approval shall be obtained prior to study start.

No deviations from, or changes to, the protocol shall be initiated without prior written IEC approvals of an appropriate amendment, except when necessary to eliminate immediate hazards to the participants or when the change(s) involves only logistical or administrative aspects of the study (e.g., change of monitor(s), telephone number(s)). The investigator shall provide to the sponsors a statement from the IEC confirming the IEC is organized and operates according to GCP and applicable laws and regulations.

12.2 PROTOCOL AMENDMENTS

Any significant change in the study protocol shall be addressed in a written protocol amendment, which will be signed by the investigator(s) and the sponsors. It is the investigator's responsibility to submit protocol amendments to the IECs and to obtain written approval where required.

In some cases, protocol amendments may also be submitted to DCGI.

A protocol amendment may be implemented after it has been approved by IECs. In the case of a protocol change intended to eliminate an apparent immediate hazard to participants, the change may be implemented immediately. In this case, the change must be later documented in an amendment and reported to the IECs as soon as possible. Amendments affecting only logistical or administrative aspects of the study may not require formal IEC approval. Logistical and administrative amendments (e.g., concerning a change of telephone number) shall be submitted to the IECs for information purposes. However, the investigator must provide the sponsors with written verification that such logistical or administrative amendments are submitted to the relevant IECs.

12.3 PARTICIPANT INFORMATION AND INFORMED CONSENT

Prior to including any participant in the clinical study, his/her free and expressed informed consent must be obtained in writing. Consent must be given with free will of choice, and without inducement.

The investigator or his/her designee shall provide to each potential participant sufficient knowledge and understanding of the nature of the proposed research, the anticipated risks and potential benefits, and the requirements of the research to be able to make an informed decision. The investigator shall give the participants ample time and opportunity to inquire about details of the study and ask any questions.

The process for obtaining the informed consent of the participant shall be in accordance with the recommendations in the New Drugs and Clinical Trials Rules, 2019.

The written informed consent must be signed and dated by both investigator/designee and participant prior to any study related procedure. In case of illiterate individuals, the study will be explained to them by the investigator or his/her designee and the Informed consent form (ICF) read for them in the presence of an impartial witness. The witness shall personally sign and date the consent form while a fingerprint will be requested from illiterate individuals. The process of informed consent should be described in source template.

Original ICF must be kept on file by the investigator for possible inspection by IECs member, regulatory authorities and the sponsors (or their designees). Participant must receive a copy of the signed ICF, and any subsequent updates or amendments.
The study monitor shall check the documentation of the individual ICF during each monitoring visit.

12.4 PARTICIPANT CONFIDENTIALITY

The investigator(s) must ensure that participant confidentiality is maintained. Personal identifiers will not be included in any study reports. Participants will be identified by the screening number and by participant initials. If a participant’s name appears on any other

document (e.g., pathologist report), it will be obliterated before the copy of the document is supplied to the sponsor/designee. Study findings stored on a computer will subject to local data protection laws. Participant will be informed that representatives of the sponsor, IEC, or regulatory authorities may inspect their medical records to verify the information collected, and that all personal information made available for inspection will be handled in the strictest confidence.

12.5 ETHICAL CONDUCT OF THE STUDY

This study will be conducted in compliance with:

1. The approved clinical trial protocol,
2. ICH-GCP guidelines.
3. Current revision of the Declaration of Helsinki (Revised Fortaleza, 2013).
4. ICH Harmonized Tripartite Guideline for Good Clinical Practice (E6) 1996.
5. ‘Guidelines for Clinical Trials on Pharmaceutical Products in India – GCP Guidelines’ issued by Central Drugs Standard Control Organization (CDSCO), Ministry of Health, Government of India in 2005.
6. New Drugs and Clinical Trials Rules, 2019 and any amendment thereof
7. ‘Ethical Guidelines for Biomedical Research on Human Subjects’ issued by Indian Council of Medical Research, 2017.

13. DATA HANDLING AND RECORD KEEPING

In accordance with applicable regulatory requirements, following closure of the study, the investigator/site/institution will maintain a copy of all essential documents in a secure and designated location at the study site. Essential documents shall be retained for at least 5 years after the completion or discontinuation of the study. Sponsor will notify the investigator/institution when the study-related records are no longer required. The investigator agrees to adhere to the document retention procedures by signing the protocol. Essential documents include:

1. Signed protocol and all amendments;
2. Ethics committee approval for the study protocol and all amendments;
3. All source documents;
4. eCRF records;

5. Study Participant Informed Consent and
6. Any other pertinent study document.

The document should not be destroyed without the written permission from SIIPL. It is responsibility of SIIPL to inform the study Investigator when these documents no longer need to be retained.

14. INSURANCE AND COMPENSATION OF STUDY PARTICIPANTS

All the study participants in this study are insured by Sponsor against any injury caused by any AE causally related to the study investigational product.
The cost of medical care needed for treatment of vaccine related AEs (including SAEs) occurring among trial participants will be borne by sponsor and as required by the Rules and Regulations passed by DCGI. In case DCGI directs to pay compensation for any AE, sponsor will pay the same and the details of compensation provided would be intimated to the office of the DCGI.

Pending respective site’s IEC approval, participants will be compensated for their time in this study, and reimbursed for travel to study visits. The study ICF will state the plan for reimbursement. Study participants will not be charged for study vaccinations, research clinic visits, research-related examinations, or research-related laboratory tests.

PI and delegated study staff as well as IEC members will be insured by Sponsor for this study as per regulatory and ethical requirements.

15. PUBLICATION POLICY & CONFIDENTIALITY

SIIPL hold the exclusive rights to publish the study results. Due credit will be given to the investigators in case the results of the study are published.

All proprietary or confidential information communicated to the investigator by or for SIIPL or communicated to the investigator during the course of and/or as a result of the clinical study is the exclusive property of SIIPL, and the investigator shall ensure that the same shall be kept strictly confidential by him/her and any other person connected with the clinical study and

shall not be disclosed, either orally or in written form, by him/her or such person to any third party without the prior written consent of SIIPL.

The investigator shall communicate the results of the clinical study promptly to SIIPL.

All rights and interests worldwide in any inventions, know-how, or other intellectual or industrial property rights which arise during the course of and/or as a result of the clinical study which is the subject of this protocol or which otherwise arise from the information or materials supplied under this protocol, shall be assigned to, vest in and remain the property of SIIPL.

16. REFERENCES

1. Zhu, N. et al. A Novel Coronavirus from Patients with Pneumonia in China, 2019. New England Journal of Medicine 382, 727-733, doi:10.1056/NEJMoa2001017 (2020).
2. Lu, R. Et al. Genomic characterisation and epidemiology of 2019 novel coronavirus: implications for virus origins and receptor binding. Lancet (London, England) 395, 565-574, doi:10.1016/S0140-6736(20)30251-8 (2020).
3. https://covid19.who.int/. Accessed 31 Dec 2021
4. COVID-19 Updates by Ministry of Health and Family Welfare, Government of India. Available at https://www.mygov.in/covid-19. Accessed 31 Dec 2021
5. Li, F. Structure, Function, and Evolution of Coronavirus Spike Proteins. Annual review of virology 3, 237-261, doi:10.1146/annurev-virology-110615-042301 (2016).
6. Dan, J.M., et al., Immunological memory to SARS-CoV-2 assessed for up to 8 months after infection. Science, 2021. 371(6529).
7. Wu, K., et al., Serum Neutralizing Activity Elicited by mRNA-1273 Vaccine. N Engl J Med, 2021.
8. Mallory R et al. Immunogenicity and Safety Following a Homologous Booster Dose of a SARS-CoV-2 recombinant spike protein vaccine (NVX-CoV2373): A Phase 2 Randomized Placebo-Controlled Trial. https://www.medrxiv.org/content/10.1101/2021.12.23.21267374v1.full.pdf. Accessed on 31 Dec 2021
9. Wrapp D, Wang N, Corbett KS, Goldsmith JA, Hsieh CL, Abiona O, Graham BS, McLellan JS. Cryo-EM structure of the 2019-nCoV spike in the prefusion conformation. Science. 2020 Mar 13;367(6483):1260-1263.
10. Coffman RL, Sher A, Seder RA. Vaccine adjuvants: putting innate immunity to work. Immunity. 2010 Oct 29;33(4):492-503.
11. Liu YV, Massare MJ, Barnard DL, Kort T, Nathan M, Wang L, Smith G. Chimeric severe acute respiratory syndrome coronavirus (SARS-CoV) S glycoprotein and influenza matrix 1 efficiently form virus-like particles (VLPs) that protect mice against challenge with SARS-CoV. Vaccine. 2011 Sep 2;29(38):6606-13.
12. Coleman CM, Venkataraman T, Liu YV, Glenn GM, Smith GE, Flyer DC, Frieman MB. MERS-CoV spike nanoparticles protect mice from MERS-CoV infection. Vaccine. 2017 Mar 14;35(12):1586-1589.
13. Bengtsson KL, Song H, Stertman L, et al. Matrix-M adjuvant enhances antibody, cellular and protective immune responses of a Zaire Ebola/Makona virus glycoprotein (GP) nanoparticle vaccine in mice. Vaccine. 2016 Apr 7;34(16):1927-35.
14. Keech C, Albert G, Cho I, Robertson A, Reed P, Neal S, et al. Phase 1-2 Trial of a SARS-CoV-2 Recombinant Spike Protein Nanoparticle Vaccine. N Engl J Med. 2020 Sep 2. doi: 10.1056/NEJMoa2026920. Epub ahead of print. PMID: 32877576.
15. Formica N, Mallory R, Albert G, et al. Different dose regimens of a SARS-CoV-2

recombinant spike protein vaccine (NVX-CoV2373) in younger and older adults: A phase 2 randomized placebo-controlled trial. PLoS Med. 2021 Oct 1;18(10):e1003769.

16. Heath PT, Galiza EP, Baxter DN, et al. Safety and Efficacy of NVX-CoV2373 Covid-19 Vaccine. N Engl J Med. 2021 Sep 23;385(13):1172-1183.
17. Toback S, Galiza E, Cosgrove C, et al. Safety, immunogenicity, and efficacy of a COVID-19 vaccine (NVX-CoV2373) co-administered with seasonal influenza vaccines: an exploratory substudy of a randomised, observer-blinded, placebo-controlled, phase 3 trial. Lancet Respir Med. 2021 Nov 17:S2213-2600(21)00409-4.
18. Shinde V, Bhikha S, Hoosain Z et al. Efficacy of NVX-CoV2373 Covid-19 Vaccine against the B.1.351 Variant. N Engl J Med. 2021 May 20;384(20):1899-1909.
19. Dunkle LM, Kotloff KL, Gay CL, et al. Efficacy and Safety of NVX-CoV2373 in Adults in the United States and Mexico. N Engl J Med. 2021 Dec 15:NEJMoa2116185. doi: 10.1056/NEJMoa2116185. Epub ahead of print.
20. Munro APS, Janani L, Cornelius V, et al. Safety and immunogenicity of seven COVID-19 vaccines as a third dose (booster) following two doses of ChAdOx1 nCov-19 or BNT162b2 in the UK (COV-BOOST): a blinded, multicentre, randomised, controlled, phase 2 trial. Lancet. 2021 Dec 18;398(10318):2258-2276. Erratum in: Lancet. 2021 Dec 18;398(10318):2246.

APPENDICES

I. Listings of AESI including PIMMCs and AESI related to COVID-19

II. COVID-19 disease symptoms and severity definition

III. Division of AIDS (DAIDS) Table for Grading the Severity of Adult and Pediatric Adverse Events, corrected version 2.1, July 2017

IV. Declaration of Helsinki

V. Sponsor's Signature Page

VI. Investigator's statement of compliance

VII. Study Sites and Principal Investigator Information

Appendix I

Listings of AESI including PIMMCs and AESI related to COVID-19

APPENDIX I: LISTINGS OF ADVERSE EVENTSOF SPECIALINTEREST

Because it has been hypothesized that immunizations with or without adjuvant may be associated with autoimmunity, sponsors need to instruct investigators to be especially vigilant regarding the Potential Immune-Mediated Medical Conditions(PIMMC) listed in the table below. Note that this is not specific to SARS-CoV-2 rS vaccine or Matrix-M1 adjuvant; and there is no current evidence to suggest that the study products in this study are, or are not, associated with these illnesses. The list is not intended to be exhaustive, nor does it exclude the possibility that other diagnoses may be AESI.

TABLE 1 POTENTIAL IMMUNE-MEDIATED MEDICAL CONDITIONS (PIMMC)

**Categories**	**Diagnoses (as MedDRA Preferred Terms)**
Neuroinflammatory Disorders:	Acute disseminated encephalomyelitis (including site specific variants: e.g., non-infectious encephalitis, encephalomyelitis, myelitis, myeloradiculomyelitis), cranial nerve disorders including paralyses/paresis (e.g., Bell's palsy), generalized convulsion, Guillain-Barre syndrome (including Miller Fisher syndrome and other variants), immune-mediated peripheral neuropathies and plexopathies (including chronic inflammatory demyelinating polyneuropathy, multifocal motor neuropathy and polyneuropathies associated with monoclonal gammopathy), myasthenia gravis, multiple sclerosis, narcolepsy, optic neuritis, transverse myelitis, uveitis
Musculoskeletal and Connective Tissue Disorders:	Antisynthetase syndrome, dermatomyositis, juvenile chronic arthritis (including Still's disease), mixed connective tissue disorder, polymyalgia rheumatic, polymyositis, psoriatic arthropathy, relapsing polychondritis, rheumatoid arthritis, scleroderma (including diffuse systemic form and CREST syndrome), spondyloarthritis (including ankylosing spondylitis, reactive arthritis [Reiter's Syndrome] and undifferentiated spondyloarthritis), systemic lupus erythematosus, systemic sclerosis, Sjogren's syndrome
Vasculidities:	Large vessels vasculitis (including giant cell arteritis such as Takayasu's arteritis and temporal arteritis), medium sized and/or small vessels vasculitis (including polyarteritis nodosa, Kawasaki's disease, microscopic polyangiitis, Wegener's granulomatosis, Churg–Strauss syndrome [allergic granulomatous angiitis], Buerger's disease [thromboangiitis obliterans], necrotizing vasculitis and anti-neutrophil cytoplasmic antibody [ANCA] positive vasculitis [type unspecified], Henoch-Schonlein purpura, Behcet's syndrome, leukocytoclastic vasculitis)
Gastrointestinal Disorders:	Crohn's disease, celiac disease, ulcerative colitis, ulcerative proctitis
Hepatic Disorders:	Autoimmune hepatitis, autoimmune cholangitis, primary sclerosing cholangitis, primary biliary cirrhosis
Renal Disorders:	Autoimmune glomerulonephritis (including IgA nephropathy, glomerulonephritis rapidly progressive, membranous glomerulonephritis, membranoproliferative glomerulonephritis, and mesangioproliferative glomerulonephritis
Cardiac Disorders:	Autoimmune myocarditis/cardiomyopathy

Categories	Diagnoses (as MedDRA Preferred Terms)
Skin Disorders:	Alopecia areata, psoriasis, vitiligo, Raynaud's phenomenon, erythema nodosum, autoimmune bullous skin diseases (including pemphigus, pemphigoid and dermatitis herpetiformis), cutaneous lupus erythematosus, morphoea, lichen planus, Stevens-Johnson syndrome, Sweet's syndrome
Hematologic Disorders:	Autoimmune hemolytic anemia, autoimmune thrombocytopenia, antiphospholipid syndrome, thrombocytopenia
Metabolic Disorders:	Autoimmune thyroiditis, Grave's or Basedow's disease, Hashimoto thyroiditis[a], diabetes mellitus type 1, Addison's disease
Other Disorders:	Goodpasture syndrome, idiopathic pulmonary fibrosis, pernicious anemia, sarcoidosis

a For Hashimoto thyroiditis: new onset only.

AESIs relevant to COVID-19 are listed in the table below. The list is not intended to be exhaustive, nor does it exclude the possibility that other diagnoses may be AESI. It is anticipated that additional AESI may be associated with COVID-19. Investigators should stay updated regarding such public health notifications.

TABLE 2. ADVERSE EVENTS OF SPECIAL INTEREST RELEVANT TOCOVID-19

Body System	Diagnoses[a]
Immunologic	Enhanced disease following immunization, cytokine release syndrome related to COVID-19[b], Multisystem inflammatory syndrome in children (MIS-C)
Respiratory	Acute respiratory distress syndrome (ARDS)
Cardiac	Acute cardiac injury including:<br>• Microangiopathy<br>• Heart failure and cardiogenic shock<br>• Stress cardiomyopathy<br>• Coronary artery disease<br>• Arrhythmia<br>• Myocarditis, pericarditis
Hematologic	Coagulation disorder<br>• Deep vein thrombosis<br>• Pulmonary embolus<br>• Cerebrovascular stroke<br>• Limbischemia<br>• Hemorrhagic disease<br>• Thrombotic complications
Renal	Acute kidney injury
Gastrointestinal	Liver injury
Neurologic	Guillain-Barré Syndrome, anosmia, ageusia, meningoencephalitis
Dermatologic	Chilblain-like lesions, single organ cutaneous vasulitis, erythema multiforme

Abbreviations: COVID-19 = coronavirus disease 2019; DAIDS = Division of AIDS.

a COVID-19 manifestations associated with more severe presentation and decompensation with consideration of enhanced disease potential (SPEAC2020).

b Cytokine release syndrome related to COVID-19 infection is a disorder characterized by nausea, headache, tachycardia, hypotension, rash, and/or shortness of breath (DAIDS2017)

Appendix II

COVID-19 disease symptoms and severity definition

APPENDIX II. COVID-19 disease symptoms and severe COVID-19 disease definition

Table 1. Qualifying Symptoms of Suspected COVID-19 Disease

- Fever or chills
- Cough
- Shortness of breath or difficulty breathing
- Fatigue
- Muscle or body aches
- Headache
- New loss of taste or smell
- Sore throat
- Congestion or runny nose
- Nausea or vomiting
- Diarrhea

Table 2. Definition of Severe COVID-19 Disease

≥ 1 of:

- Tachypnea: ≥ 30 breaths per minute at rest
- Resting heart rate ≥ 125 beats per minute
- SpO2: ≤ 93% on room air or PAO2/FiO2 < 300
- High flow oxygen therapy or NIV/NIPPV (e.g., CPAP or BiPAP)
- Mechanical ventilation or ECMO
- One or more major organ system dysfunction or failure (e.g., cardiac/circulatory, pulmonary, renal, hepatic, and/or neurological, to be defined by diagnostic testing/clinical syndrome/interventions), including any of the following:
  - ARDS
  - Acute renal failure
  - Acute hepatic failure
  - Acute right or left heart failure

  - Septic or cardiogenic shock (with shock defined as SBP < 90 mm Hg OR DBP < 60 mm Hg
  - Acute stroke (ischemic or hemorrhagic)
  - Acute thrombotic event: AMI, DVT, PE
  - Requirement for: vasopressors, systemic corticosteroids, or hemodialysis.
- Admission to an ICU
- Death

Abbreviations: AMI = acute myocardial infarction; ARDS = acute respiratory distress syndrome; BiPAP = bi-level positiveairway pressure; CPAP = continuous positive air pressure; DBP = diastolic bloodpressure;
DVT = deep vein thrombosis; ECMO = extracorporeal membrane oxygenation; FiO2 = fraction of inspiredoxygen; ICU = intensive care unit; NIV = non-invasive ventilation;NIPPV = non-invasive positive pressure ventilation; PAO2 = partial pressure of oxygen in the alveolus;PE = pulmonary embolism; SBP = systolic blood pressure; SpO2 = oxygen saturation.

Appendix III

Division of AIDS (DAIDS) Table for Grading the Severity of Adult and Pediatric Adverse Events, corrected version 2.1, July 2017

Division of AIDS (DAIDS) Table for Grading the Severity of Adult and Pediatric Adverse Events

Corrected Version 2.1
July 2017

Division of AIDS
National Institute of Allergy and Infectious Diseases
National Institutes of Health
US Department of Health and Human Services

Table of Contents

Glossary and Acronyms 1
Introduction 3
Instructions for Use 4
Major Clinical Conditions 7
    Cardiovascular 7
    Dermatologic 9
    Endocrine and Metabolic 10
    Gastrointestinal 12
    Musculoskeletal 14
    Neurologic 15
    Pregnancy, Puerperium, and Perinatal 17
    Psychiatric 18
    Respiratory 19
    Sensory 20
    Systemic 21
    Urinary 23
Site Reactions to Injections and Infusions 24
Laboratory Values 25
    Chemistries 25
    Hematology 29
    Urinalysis 31
Appendix A. Total Bilirubin Table for Term and Preterm Neonates 32

Glossary and Acronyms

AE	Adverse event; Any unfavorable and unintended sign (including an abnormal laboratory finding), symptom, or disease temporally associated with the use of a medical treatment or procedure regardless of whether it is considered related to the medical treatment or procedure.
ALT (SGPT)	Alanine aminotransferase (*serum glutamic pyruvic transaminase)*
ANC	Absolute neutrophil count
AST (SGOT)	Aspartate aminotransferase (*serum glutamic-oxaloacetic transaminase)*
AV	Atrioventricular
Basic Self-care Functions	Adult<br>Activities such as bathing, dressing, toileting, transfer or movement, continence, and feeding.<br><br>Young Children<br>Activities that are age and culturally appropriate, such as feeding one's self with culturally appropriate eating implements.
BMI z-score	Body mass index z- score; A body reference norm. Specifically, the number of standard deviations a participant's BMI differs from the average BMI for their age, sex, and ethnicity.
BMD t-score	Bone mineral density t-score; The number of standard deviations above or below the mean bone mineral density of a healthy 30 year old adult of the same sex and ethnicity as the participant.
BMD z-score	Bone mineral density z-score; The number of standard deviations a participant's BMD differs from the average BMD for their age, sex, and ethnicity.
BPAP	Bilevel positive airway pressure; A mode used during noninvasive positive pressure ventilation.
Chemical Pregnancy	A pregnancy in which a positive pregnancy test is followed by a negative pregnancy test without evidence of a clinical pregnancy loss.
CNS	Central nervous system
CPAP	Continuous positive airway pressure
DAERS	DAIDS Adverse Experience Reporting System; An internet-based system developed for clinical research sites to report Expedited Adverse Events (EAEs) to DAIDS. It facilitates timely EAE report submission and serves as a centralized location for accessing and processing EAE information for reporting purposes.
Disability	A substantial disruption of a person's ability to conduct normal life functions.
ECG	Electrocardiogram
eGFR	Estimated glomerular filtration rate
Hospitalization	Does not include the following hospital admissions: under 24 hours, unrelated to an adverse event (e.g., for labor and delivery, cosmetic surgery, social or administrative for temporary placement [for lack of a place to sleep]), protocol-specified, and for diagnosis or therapy of a condition that existed before the receipt of a study agent and which has not increased in severity or frequency.
INR	International normalized ratio

Glossary and Acronyms

Intervention	Medical, surgical, or other procedures recommended or provided by a healthcare professional for the treatment of an adverse event.
IV	Intravenous
IVIG	Intravenous immune globulin
LDL	Low density lipoprotein
LLN	Lower limit of normal
Life-threatening AE	Any adverse event that places the participant, in the view of the investigator, at immediate risk of death from the reaction when it occurred (i.e., it does not include a reaction that would have caused death if it had occurred in a more severe form).
NA	Not applicable
Participant ID	The identification number assigned to a study participant which is used to track study-related documentation, including any reported AEs.
PR Interval	The interval between the beginning of the P wave and the beginning of the QRS complex of an electrocardiogram that represents the time between the beginning of the contraction of the atria and the beginning of the contraction of the ventricles.
PT	Prothrombin time
PTT	Partial thromboplastin time
QTc Interval	The measure of time between the onset of ventricular depolarization and completion of ventricular repolarization corrected for ventricular rate.
RBC	Red blood cell
SI	Standard international unit
ULN	Upper limit of normal
Usual Social & Functional Activities	Activities which adults and children perform on a routine basis and those which are part of regular activities of daily living, for example:<br><br>Adults<br>Adaptive tasks and desirable activities, such as going to work, shopping, cooking, use of transportation, or pursuing a hobby.<br><br>Young Children<br>Activities that are age and culturally appropriate, such as social interactions, play activities, or learning tasks.
WBC	White blood cell
WHO	World Health Organization
WNL	Within normal limits

Introduction

The Division of AIDS (DAIDS) oversees more than 300 clinical trials domestically and internationally, which evaluate the safety and efficacy of therapeutic products, vaccines, and other preventive modalities. Adverse event (AE) data collected during these clinical trials form the basis for subsequent safety and efficacy analyses of pharmaceutical products and medical devices. Incorrect and inconsistent AE severity grading can lead to inaccurate data analyses and interpretation, which in turn can impact the safety and well-being of clinical trial participants and future patients using pharmaceutical products.

Over the years, DAIDS scientific knowledge and experience have expanded, necessitating revisions of the DAIDS grading table which serves as a guide for assessing the severity of AEs (including clinical and laboratory abnormalities) in participants enrolled in DAIDS-sponsored and -supported clinical trials. The *Division of AIDS (DAIDS) Table for Grading the Severity of Adult and Pediatric Adverse Events, Corrected Version 2.1 (July 2017)* updates and replaces version 2.1 (March 2017).

DAIDS is grateful to the DAIDS Grading Table Working Group, numerous government and non-government affiliated medical subject matter experts and reviewers who were instrumental in the revision of the DAIDS grading table.

Instructions for Use

General Considerations
The *Division of AIDS (DAIDS) Table for Grading the Severity of Adult and Pediatric Adverse Events, Version 2.1* consists of parameters, or AEs, with severity grading guidance that are to be used in DAIDS clinical trials for safety data reporting to maintain accuracy and consistency in the evaluation of AEs. The term "severe" is not the same as the term "serious" in classifying AEs. The severity of a specific event describes its intensity, and it is the intensity which is graded. Seriousness, which is not graded, relates to an outcome of an AE and is a regulatory definition.

Clinical sites are encouraged to report parameters in the DAIDS grading table as they are written to maintain data consistency across clinical trials. However, since some parameters can be reported with more specificity, clinical sites are encouraged to report parameters that convey additional clinical information. For example, diarrhea could be reported as neonatal diarrhea; seizures, as febrile seizures; and pain, as jaw pain.

The DAIDS grading table provides an AE severity grading scale ranging from grades 1 to 5 with descriptions for each AE based on the following general guidelines:

- Grade 1 indicates a mild event
- Grade 2 indicates a moderate event
- Grade 3 indicates a severe event
- Grade 4 indicates a potentially life-threatening event
- Grade 5 indicates death (*Note*: This grade is not specifically listed on each page of the grading table).

Other points to consider include:

- Use age and sex values as applicable.
- Unless noted, laboratory values are for term neonates. Preterm neonates should be assessed using local laboratory normal ranges.
- Where applicable, Standard International (SI) units are included in italics.

Selecting and Reporting a Primary AE Term
When selecting a primary AE term to report, sites should select the term that best describes what occurred to the participant. For example, a participant may present with itching, urticaria, flushing, angioedema of the face, and dyspnea. If the underlying diagnosis is determined to be an acute allergic reaction, sites should report "Acute Allergic Reaction" as the primary AE term.

Primary AE terms should be reported using the DAIDS Adverse Experience Reporting System (DAERS) only if they meet expedited reporting criteria. However, all primary AE terms should be reported using protocol-specific case report forms (CRFs). Because the reported information is stored in different databases (i.e., safety and clinical), sites should report primary AE terms using the same terminology for data consistency.

Instructions for Use

When reporting using DAERS, other clinically significant events associated with a primary AE term that more fully describe the nature, severity, or complications of the primary AE term should be entered in the "Other Events" section. However, the severity grade for these events must be lower than or equal to the severity grade of the primary AE term. In the example above, dyspnea and angioedema of the face may be entered in the "Other Events" section, because they are more descriptive and provide additional information on the severity of the acute allergic reaction. However, their severity grades must be lower than or equal to the severity grade of the primary AE term of "Acute Allergic Reaction".

Differences exist in the reporting and recording of information (e.g., signs and symptoms, clinically significant events) in DAERS and CRFs. Therefore, sites should refer to their protocols and CRF requirements for further instructions.

Grading Adult and Pediatric AEs
When a single parameter is not appropriate for grading an AE in both adult and pediatric populations, separate parameters with specified age ranges are provided. If no distinction between adult and pediatric populations has been made, the listed parameter should be used for grading an AE in both populations.

Reporting Pregnancy Outcomes
In the *Pregnancy, Puerperium, and Perinatal* section, all parameters are pregnancy outcomes and should be reported using the mother's participant ID. If an infant is not enrolled in the same study as the mother, any identified birth defects should be reported using the mother's participant ID. However, if an infant is enrolled in the same study as the mother or in another study, any identified birth defects should be reported using the infant's participant ID. Sites should refer to the applicable network standards for reporting abnormal pregnancy outcomes on the CRFs.

Determining Severity Grade for Parameters between Grades
If the severity of an AE could fall in either one of two grades (i.e., the severity of an AE could be either grade 2 or grade 3), sites should select the higher of the two grades.

Laboratory Values
General. An asymptomatic, abnormal laboratory finding without an accompanying AE should not be reported to DAIDS in an expedited timeframe unless it meets protocol-specific reporting requirements. Sites should refer to the applicable network standards for reporting abnormal laboratory findings on the clinical case report forms.

Values below Grade 1. Any laboratory value that is between the ULN and grade 1 (for high values) or the LLN and grade 1 (for low values) should not be graded or reported as an AE. Sites should consult the *Manual for Expedited Reporting of Adverse Events to DAIDS, Version 2.0* and their protocol when making an assessment of the need to report an AE.

Overlap of Local Laboratory Normal Values with Grading Table Ranges. When local laboratory normal values fall within grading table laboratory ranges, the severity grading is based on the ranges in the grading table unless there is a protocol-specific grading criterion for the laboratory

value. For example, “Magnesium, Low" has a grade 1 range of 1.2 to < 1.4 mEq/L, while a particular laboratory’s normal range for magnesium may be 1.3 to 2.8 mEq/L. If a study participant’s magnesium laboratory value is 1.3 mEq/L, the laboratory value should be graded as grade 1.

Appendix Usage
Appendix A takes priority over the main grading table in all assessments of total bilirubin for term and preterm neonates.

Using Addenda 1-3: Grading Tables Used in Microbicide Studies
In protocols involving topical application of products to the female and male genital tracts or rectum, strong consideration should be given to using Addenda 1-3 (see below) as the primary grading tables for these areas. Although these grading tables are used specifically in microbicide studies, they may be used in other protocols as adjuncts to the main grading table (i.e., the *Division of AIDS (AIDS) Table for Grading the Severity of Adult and Pediatric Adverse Events, Version 2.0*). It should be clearly stated in a protocol which addendum is being used as the primary grading table (and thus takes precedence over the main grading table) and which addendum is being used in a complementary fashion.

- Addendum 1 – Female Genital Grading Table for Use in Microbicide Studies- *http://rsc.tech-res.com/clinical-research-sites/safety-reporting/daids-grading-tables*
- Addendum 2 – Male Genital Grading Table for Use in Microbicide Studies – *http://rsc.tech-res.com/clinical-research-sites/safety-reporting/daids-grading-tables*
- Addendum 3 – Rectal Grading Table for Use in Microbicide Studies – *http://rsc.tech-res.com/clinical-research-sites/safety-reporting/daids-grading-tables*

Estimating Severity Grade for Parameters Not Identified in the Grading Table
The functional table below should be used to grade the severity of an AE that is not specifically identified in the grading table. In addition, all deaths related to an AE are to be classified as grade 5.

PARAMETER	GRADE 1 MILD	GRADE 2 MODERATE	GRADE 3 SEVERE	GRADE 4 POTENTIALLY LIFE-THREATENING
**Clinical** adverse event **NOT** identified elsewhere in the grading table	Mild symptoms causing no or minimal interference with usual social & functional activities with intervention not indicated	Moderate symptoms causing greater than minimal interference with usual social & functional activities with intervention indicated	Severe symptoms causing inability to perform usual social & functional activities with intervention or hospitalization indicated	Potentially life-threatening symptoms causing inability to perform basic self-care functions with intervention indicated to prevent permanent impairment, persistent disability, or death

Major Clinical Conditions

Cardiovascular

PARAMETER	GRADE 1 MILD	GRADE 2 MODERATE	GRADE 3 SEVERE	GRADE 4 POTENTIALLY LIFE-THREATENING
**Arrhythmia** (by ECG or physical examination) *Specify type, if applicable*	No symptoms AND No intervention indicated	No symptoms AND Non-urgent intervention indicated	Non-life-threatening symptoms AND Non-urgent intervention indicated	Life-threatening arrhythmia OR Urgent intervention indicated
**Blood Pressure Abnormalities**[1] ***Hypertension** (with the lowest reading taken after repeat testing during a visit)* *≥ 18 years of age*	140 to < 160 mmHg systolic OR 90 to < 100 mmHg diastolic	≥ 160 to < 180 mmHg systolic OR ≥ 100 to < 110 mmHg diastolic	≥ 180 mmHg systolic OR ≥ 110 mmHg diastolic	Life-threatening consequences in a participant not previously diagnosed with hypertension (e.g., malignant hypertension) OR Hospitalization indicated
< 18 years of age	> 120/80 mmHg	≥ 95th to < 99th percentile + 5 mmHg adjusted for age, height, and gender (systolic and/or diastolic)	≥ 99th percentile + 5 mmHg adjusted for age, height, and gender (systolic and/or diastolic)	Life-threatening consequences in a participant not previously diagnosed with hypertension (e.g., malignant hypertension) OR Hospitalization indicated
Hypotension	No symptoms	Symptoms corrected with oral fluid replacement	Symptoms AND IV fluids indicated	Shock requiring use of vasopressors or mechanical assistance to maintain blood pressure
**Cardiac Ischemia or Infarction** *Report only one*	NA	NA	New symptoms with ischemia (stable angina) OR New testing consistent with ischemia	Unstable angina OR Acute myocardial infarction
**Heart Failure**	No symptoms AND Laboratory or cardiac imaging abnormalities	Symptoms with mild to moderate activity or exertion	Symptoms at rest or with minimal activity or exertion (e.g., hypoxemia) OR Intervention indicated (e.g., oxygen)	Life-threatening consequences OR Urgent intervention indicated (e.g., vasoactive medications, ventricular assist device, heart transplant)

[1] Blood pressure norms for children < 18 years of age can be found in: Expert Panel on Integrated Guidelines for Cardiovascular Health and Risk Reduction in Children and Adolescents. *Pediatrics* 2011;128;S213; originally published online November 14, 2011; DOI: 10.1542/peds.2009-2107C.

Cardiovascular

PARAMETER	GRADE 1 MILD	GRADE 2 MODERATE	GRADE 3 SEVERE	GRADE 4 POTENTIALLY LIFE-THREATENING
**Hemorrhage** (with significant acute blood loss)	NA	Symptoms AND No transfusion indicated	Symptoms AND Transfusion of ≤ 2 units packed RBCs indicated	Life-threatening hypotension OR Transfusion of > 2 units packed RBCs (for children, packed RBCs > 10 cc/kg) indicated
**Prolonged PR Interval or AV Block** *Report only one* *> 16 years of age*	PR interval 0.21 to < 0.25 seconds	PR interval ≥ 0.25 seconds OR Type I 2nd degree AV block	Type II 2nd degree AV block OR Ventricular pause ≥ 3.0 seconds	Complete AV block
≤ 16 years of age	1st degree AV block (PR interval > normal for age and rate)	Type I 2nd degree AV block	Type II 2nd degree AV block OR Ventricular pause ≥ 3.0 seconds	Complete AV block
**Prolonged QTc Interval**[2]	0.45 to 0.47 seconds	> 0.47 to 0.50 seconds	> 0.50 seconds OR ≥ 0.06 seconds above baseline	Life-threatening consequences (e.g., Torsade de pointes, other associated serious ventricular dysrhythmia)
**Thrombosis or Embolism** *Report only one*	NA	Symptoms AND No intervention indicated	Symptoms AND Intervention indicated	Life-threatening embolic event (e.g., pulmonary embolism, thrombus)

[2] As per Bazett's formula.

Dermatologic

PARAMETER	GRADE 1 MILD	GRADE 2 MODERATE	GRADE 3 SEVERE	GRADE 4 POTENTIALLY LIFE-THREATENING
**Alopecia** (scalp only)	Detectable by study participant, caregiver, or physician AND Causing no or minimal interference with usual social & functional activities	Obvious on visual inspection AND Causing greater than minimal interference with usual social & functional activities	NA	NA
**Bruising**	Localized to one area	Localized to more than one area	Generalized	NA
**Cellulitis**	NA	Non-parenteral treatment indicated (e.g., oral antibiotics, antifungals, antivirals)	IV treatment indicated (e.g., IV antibiotics, antifungals, antivirals)	Life-threatening consequences (e.g., sepsis, tissue necrosis)
**Hyperpigmentation**	Slight or localized causing no or minimal interference with usual social & functional activities	Marked or generalized causing greater than minimal interference with usual social & functional activities	NA	NA
**Hypopigmentation**	Slight or localized causing no or minimal interference with usual social & functional activities	Marked or generalized causing greater than minimal interference with usual social & functional activities	NA	NA
**Petechiae**	Localized to one area	Localized to more than one area	Generalized	NA
**Pruritus**[3] (without skin lesions)	Itching causing no or minimal interference with usual social & functional activities	Itching causing greater than minimal interference with usual social & functional activities	Itching causing inability to perform usual social & functional activities	NA
**Rash** *Specify type, if applicable*	Localized rash	Diffuse rash OR Target lesions	Diffuse rash AND Vesicles or limited number of bullae or superficial ulcerations of mucous membrane limited to one site	Extensive or generalized bullous lesions OR Ulceration of mucous membrane involving two or more distinct mucosal sites OR Stevens-Johnson syndrome OR Toxic epidermal necrolysis

[3] For pruritus associated with injections or infusions, see the *Site Reactions to Injections and Infusions* section (page 23).

Endocrine and Metabolic

PARAMETER	GRADE 1 MILD	GRADE 2 MODERATE	GRADE 3 SEVERE	GRADE 4 POTENTIALLY LIFE-THREATENING
**Diabetes Mellitus**	Controlled without medication	Controlled with medication OR Modification of current medication regimen	Uncontrolled despite treatment modification OR Hospitalization for immediate glucose control indicated	Life-threatening consequences (e.g., ketoacidosis, hyperosmolar non-ketotic coma, end organ failure)
**Gynecomastia**	Detectable by study participant, caregiver, or physician AND Causing no or minimal interference with usual social & functional activities	Obvious on visual inspection AND Causing pain with greater than minimal interference with usual social & functional activities	Disfiguring changes AND Symptoms requiring intervention or causing inability to perform usual social & functional activities	NA
**Hyperthyroidism**	No symptoms AND Abnormal laboratory value	Symptoms causing greater than minimal interference with usual social & functional activities OR Thyroid suppression therapy indicated	Symptoms causing inability to perform usual social & functional activities OR Uncontrolled despite treatment modification	Life-threatening consequences (e.g., thyroid storm)
**Hypothyroidism**	No symptoms AND Abnormal laboratory value	Symptoms causing greater than minimal interference with usual social & functional activities OR Thyroid replacement therapy indicated	Symptoms causing inability to perform usual social & functional activities OR Uncontrolled despite treatment modification	Life-threatening consequences (e.g., myxedema coma)
**Lipoatrophy**[4]	Detectable by study participant, caregiver, or physician AND Causing no or minimal interference with usual social & functional activities	Obvious on visual inspection AND Causing greater than minimal interference with usual social & functional activities	Disfiguring changes	NA

[4] Definition: A disorder characterized by fat loss in the face, extremities, and buttocks.

Endocrine and Metabolic

PARAMETER	GRADE 1 MILD	GRADE 2 MODERATE	GRADE 3 SEVERE	GRADE 4 POTENTIALLY LIFE-THREATENING
**Lipohypertrophy**[5]	Detectable by study participant, caregiver, or physician AND Causing no or minimal interference with usual social & functional activities	Obvious on visual inspection AND Causing greater than minimal interference with usual social & functional activities	Disfiguring changes	NA

[5] Definition: A disorder characterized by abnormal fat accumulation on the back of the neck, breasts, and abdomen.

Gastrointestinal

PARAMETER	GRADE 1 MILD	GRADE 2 MODERATE	GRADE 3 SEVERE	GRADE 4 POTENTIALLY LIFE-THREATENING
**Anorexia**	Loss of appetite without decreased oral intake	Loss of appetite associated with decreased oral intake without significant weight loss	Loss of appetite associated with significant weight loss	Life-threatening consequences OR Aggressive intervention indicated (e.g., tube feeding, total parenteral nutrition)
**Ascites**	No symptoms	Symptoms AND Intervention indicated (e.g., diuretics, therapeutic paracentesis)	Symptoms recur or persist despite intervention	Life-threatening consequences
**Bloating or Distension**<br>*Report only one*	Symptoms causing no or minimal interference with usual social & functional activities	Symptoms causing greater than minimal interference with usual social & functional activities	Symptoms causing inability to perform usual social & functional activities	NA
**Cholecystitis**	NA	Symptoms AND Medical intervention indicated	Radiologic, endoscopic, or operative intervention indicated	Life-threatening consequences (e.g., sepsis, perforation)
**Constipation**	NA	Persistent constipation requiring regular use of dietary modifications, laxatives, or enemas	Obstipation with manual evacuation indicated	Life-threatening consequences (e.g., obstruction)
**Diarrhea**<br>*≥ 1 year of age*	Transient or intermittent episodes of unformed stools OR Increase of ≤ 3 stools over baseline per 24-hour period	Persistent episodes of unformed to watery stools OR Increase of 4 to 6 stools over baseline per 24-hour period	Increase of ≥ 7 stools per 24-hour period OR IV fluid replacement indicated	Life-threatening consequences (e.g., hypotensive shock)
< 1 year of age	Liquid stools (more unformed than usual) but usual number of stools	Liquid stools with increased number of stools OR Mild dehydration	Liquid stools with moderate dehydration	Life-threatening consequences (e.g., liquid stools resulting in severe dehydration, hypotensive shock)
**Dysphagia or Odynophagia**<br>*Report only one and specify location*	Symptoms but able to eat usual diet	Symptoms causing altered dietary intake with no intervention indicated	Symptoms causing severely altered dietary intake with intervention indicated	Life-threatening reduction in oral intake
**Gastrointestinal Bleeding**	Not requiring intervention other than iron supplement	Endoscopic intervention indicated	Transfusion indicated	Life-threatening consequences (e.g., hypotensive shock)

Gastrointestinal

PARAMETER	GRADE 1 MILD	GRADE 2 MODERATE	GRADE 3 SEVERE	GRADE 4 POTENTIALLY LIFE-THREATENING
**Mucositis or Stomatitis** *Report only one and specify location*	Mucosal erythema	Patchy pseudomembranes or ulcerations	Confluent pseudomembranes or ulcerations OR Mucosal bleeding with minor trauma	Life-threatening consequences (e.g., aspiration, choking) OR Tissue necrosis OR Diffuse spontaneous mucosal bleeding
**Nausea**	Transient (< 24 hours) or intermittent AND No or minimal interference with oral intake	Persistent nausea resulting in decreased oral intake for 24 to 48 hours	Persistent nausea resulting in minimal oral intake for > 48 hours OR Rehydration indicated (e.g., IV fluids)	Life-threatening consequences (e.g., hypotensive shock)
**Pancreatitis**	NA	Symptoms with hospitalization not indicated	Symptoms with hospitalization indicated	Life-threatening consequences (e.g., circulatory failure, hemorrhage, sepsis)
**Perforation** (colon or rectum)	NA	NA	Intervention indicated	Life-threatening consequences
**Proctitis**	Rectal discomfort with no intervention indicated	Symptoms causing greater than minimal interference with usual social & functional activities OR Medical intervention indicated	Symptoms causing inability to perform usual social & functional activities OR Operative intervention indicated	Life-threatening consequences (e.g., perforation)
**Rectal Discharge**	Visible discharge	Discharge requiring the use of pads	NA	NA
**Vomiting**	Transient or intermittent AND No or minimal interference with oral intake	Frequent episodes with no or mild dehydration	Persistent vomiting resulting in orthostatic hypotension OR Aggressive rehydration indicated (e.g., IV fluids)	Life-threatening consequences (e.g., hypotensive shock)

Musculoskeletal

PARAMETER	GRADE 1 MILD	GRADE 2 MODERATE	GRADE 3 SEVERE	GRADE 4 POTENTIALLY LIFE-THREATENING
**Arthralgia**	Joint pain causing no or minimal interference with usual social & functional activities	Joint pain causing greater than minimal interference with usual social & functional activities	Joint pain causing inability to perform usual social & functional activities	Disabling joint pain causing inability to perform basic self-care functions
**Arthritis**	Stiffness or joint swelling causing no or minimal interference with usual social & functional activities	Stiffness or joint swelling causing greater than minimal interference with usual social & functional activities	Stiffness or joint swelling causing inability to perform usual social & functional activities	Disabling joint stiffness or swelling causing inability to perform basic self-care functions
**Myalgia** (generalized)	Muscle pain causing no or minimal interference with usual social & functional activities	Muscle pain causing greater than minimal interference with usual social & functional activities	Muscle pain causing inability to perform usual social & functional activities	Disabling muscle pain causing inability to perform basic self-care functions
**Osteonecrosis**	NA	No symptoms but with radiographic findings AND No operative intervention indicated	Bone pain with radiographic findings OR Operative intervention indicated	Disabling bone pain with radiographic findings causing inability to perform basic self-care functions
**Osteopenia**[6] *≥ 30 years of age*	BMD t-score -2.5 to -1	NA	NA	NA
< 30 years of age	BMD z-score -2 to -1	NA	NA	NA
**Osteoporosis**[6] *≥ 30 years of age*	NA	BMD t-score < -2.5	Pathologic fracture (e.g., compression fracture causing loss of vertebral height)	Pathologic fracture causing life-threatening consequences
< 30 years of age	NA	BMD z-score < -2	Pathologic fracture (e.g., compression fracture causing loss of vertebral height)	Pathologic fracture causing life-threatening consequences

[6] BMD t and z scores can be found in: Kanis JA on behalf of the World Health Organization Scientific Group (2007). Assessment of osteoporosis at the primary health-care level. Technical Report. World Health Organization Collaborating Centre for Metabolic Bone Diseases, University of Sheffield, UK. 2007: Printed by the University of Sheffield.

Neurologic

PARAMETER	GRADE 1 MILD	GRADE 2 MODERATE	GRADE 3 SEVERE	GRADE 4 POTENTIALLY LIFE-THREATENING
**Acute CNS Ischemia**	NA	NA	Transient ischemic attack	Cerebral vascular accident (e.g., stroke with neurological deficit)
**Altered Mental Status** (for Dementia, see *Cognitive, Behavioral, or Attentional Disturbance* below)	Changes causing no or minimal interference with usual social & functional activities	Mild lethargy or somnolence causing greater than minimal interference with usual social & functional activities	Confusion, memory impairment, lethargy, or somnolence causing inability to perform usual social & functional activities	Delirium OR Obtundation OR Coma
**Ataxia**	Symptoms causing no or minimal interference with usual social & functional activities OR No symptoms with ataxia detected on examination	Symptoms causing greater than minimal interference with usual social & functional activities	Symptoms causing inability to perform usual social & functional activities	Disabling symptoms causing inability to perform basic self-care functions
**Cognitive, Behavioral, or Attentional Disturbance** (includes dementia and attention deficit disorder)<br>*Specify type, if applicable*	Disability causing no or minimal interference with usual social & functional activities OR Specialized resources not indicated	Disability causing greater than minimal interference with usual social & functional activities OR Specialized resources on part-time basis indicated	Disability causing inability to perform usual social & functional activities OR Specialized resources on a full-time basis indicated	Disability causing inability to perform basic self-care functions OR Institutionalization indicated
**Developmental Delay**<br>*< 18 years of age*<br>*Specify type, if applicable*	Mild developmental delay, either motor or cognitive, as determined by comparison with a developmental screening tool appropriate for the setting	Moderate developmental delay, either motor or cognitive, as determined by comparison with a developmental screening tool appropriate for the setting	Severe developmental delay, either motor or cognitive, as determined by comparison with a developmental screening tool appropriate for the setting	Developmental regression, either motor or cognitive, as determined by comparison with a developmental screening tool appropriate for the setting
**Headache**	Symptoms causing no or minimal interference with usual social & functional activities	Symptoms causing greater than minimal interference with usual social & functional activities	Symptoms causing inability to perform usual social & functional activities	Symptoms causing inability to perform basic self-care functions OR Hospitalization indicated OR Headache with significant impairment of alertness or other neurologic function

Neurologic

PARAMETER	GRADE 1 MILD	GRADE 2 MODERATE	GRADE 3 SEVERE	GRADE 4 POTENTIALLY LIFE-THREATENING
**Neuromuscular Weakness** (includes myopathy and neuropathy)<br>*Specify type, if applicable*	Minimal muscle weakness causing no or minimal interference with usual social & functional activities <u>OR</u> No symptoms with decreased strength on examination	Muscle weakness causing greater than minimal interference with usual social & functional activities	Muscle weakness causing inability to perform usual social & functional activities	Disabling muscle weakness causing inability to perform basic self-care functions <u>OR</u> Respiratory muscle weakness impairing ventilation
**Neurosensory Alteration** (includes paresthesia and painful neuropathy)<br>*Specify type, if applicable*	Minimal paresthesia causing no or minimal interference with usual social & functional activities <u>OR</u> No symptoms with sensory alteration on examination	Sensory alteration or paresthesia causing greater than minimal interference with usual social & functional activities	Sensory alteration or paresthesia causing inability to perform usual social & functional activities	Disabling sensory alteration or paresthesia causing inability to perform basic self-care functions
**Seizures**<br>***New Onset Seizure***<br>*≥ 18 years of age*	NA	NA	1 to 3 seizures	Prolonged and repetitive seizures (e.g., status epilepticus) <u>OR</u> Difficult to control (e.g., refractory epilepsy)
< 18 years of age (includes new or pre-existing febrile seizures)	Seizure lasting < 5 minutes with < 24 hours postictal state	Seizure lasting 5 to < 20 minutes with < 24 hours postictal state	Seizure lasting ≥ 20 minutes <u>OR</u> > 24 hours postictal state	Prolonged and repetitive seizures (e.g., status epilepticus) <u>OR</u> Difficult to control (e.g., refractory epilepsy)
Pre-existing Seizure	NA	Increased frequency from previous level of control without change in seizure character	Change in seizure character either in duration or quality (e.g., severity or focality)	Prolonged and repetitive seizures (e.g., status epilepticus) <u>OR</u> Difficult to control (e.g., refractory epilepsy)
**Syncope**	Near syncope without loss of consciousness (e.g., pre-syncope)	Loss of consciousness with no intervention indicated	Loss of consciousness <u>AND</u> Hospitalization or intervention required	NA

Pregnancy, Puerperium, and Perinatal

PARAMETER	GRADE 1 MILD	GRADE 2 MODERATE	GRADE 3 SEVERE	GRADE 4 POTENTIALLY LIFE-THREATENING
**Stillbirth** (report using mother's participant ID)<br>*Report only one*	NA	NA	Fetal death occurring at ≥ 20 weeks gestation	NA
**Preterm Birth** (report using mother's participant ID)	Live birth at 34 to < 37 weeks gestational age	Live birth at 28 to < 34 weeks gestational age	Live birth at 24 to < 28 weeks gestational age	Live birth at < 24 weeks gestational age
**Spontaneous Abortion or Miscarriage**[7] (report using mother's participant ID)<br>*Report only one*	Chemical pregnancy	Uncomplicated spontaneous abortion or miscarriage	Complicated spontaneous abortion or miscarriage	NA

[7] Definition: A pregnancy loss occurring at < 20 weeks gestational age.

Psychiatric

PARAMETER	GRADE 1 MILD	GRADE 2 MODERATE	GRADE 3 SEVERE	GRADE 4 POTENTIALLY LIFE-THREATENING
**Insomnia**	Mild difficulty falling asleep, staying asleep, or waking up early causing no or minimal interference with usual social & functional activities	Moderate difficulty falling asleep, staying asleep, or waking up early causing more than minimal interference with usual social & functional activities	Severe difficulty falling asleep, staying asleep, or waking up early causing inability to perform usual social & functional activities requiring intervention or hospitalization	NA
**Psychiatric Disorders** (includes anxiety, depression, mania, and psychosis) *Specify disorder*	Symptoms with intervention not indicated OR Behavior causing no or minimal interference with usual social & functional activities	Symptoms with intervention indicated OR Behavior causing greater than minimal interference with usual social & functional activities	Symptoms with hospitalization indicated OR Behavior causing inability to perform usual social & functional activities	Threatens harm to self or others OR Acute psychosis OR Behavior causing inability to perform basic self-care functions
**Suicidal Ideation or Attempt** *Report only one*	Preoccupied with thoughts of death AND No wish to kill oneself	Preoccupied with thoughts of death AND Wish to kill oneself with no specific plan or intent	Thoughts of killing oneself with partial or complete plans but no attempt to do so OR Hospitalization indicated	Suicide attempted

Respiratory

PARAMETER	GRADE 1 MILD	GRADE 2 MODERATE	GRADE 3 SEVERE	GRADE 4 POTENTIALLY LIFE-THREATENING
**Acute Bronchospasm**	Forced expiratory volume in 1 second or peak flow reduced to ≥ 70 to < 80% <u>OR</u> Mild symptoms with intervention not indicated	Forced expiratory volume in 1 second or peak flow 50 to < 70% <u>OR</u> Symptoms with intervention indicated <u>OR</u> Symptoms causing greater than minimal interference with usual social & functional activities	Forced expiratory volume in 1 second or peak flow 25 to < 50% <u>OR</u> Symptoms causing inability to perform usual social & functional activities	Forced expiratory volume in 1 second or peak flow < 25% <u>OR</u> Life-threatening respiratory or hemodynamic compromise <u>OR</u> Intubation
**Dyspnea or Respiratory Distress**<br>*Report only one*	Dyspnea on exertion with no or minimal interference with usual social & functional activities <u>OR</u> Wheezing <u>OR</u> Minimal increase in respiratory rate for age	Dyspnea on exertion causing greater than minimal interference with usual social & functional activities <u>OR</u> Nasal flaring <u>OR</u> Intercostal retractions <u>OR</u> Pulse oximetry 90 to < 95%	Dyspnea at rest causing inability to perform usual social & functional activities <u>OR</u> Pulse oximetry < 90%	Respiratory failure with ventilator support indicated (e.g., CPAP, BPAP, intubation)

Sensory

PARAMETER	GRADE 1 MILD	GRADE 2 MODERATE	GRADE 3 SEVERE	GRADE 4 POTENTIALLY LIFE-THREATENING
**Hearing Loss**<br>*≥ 12 years of age*	NA	Hearing aid or intervention not indicated	Hearing aid or intervention indicated	Profound bilateral hearing loss (> 80 dB at 2 kHz and above) <u>OR</u> Non-serviceable hearing (i.e., >50 dB audiogram and <50% speech discrimination)
< 12 years of age (based on a 1, 2, 3, 4, 6 and 8 kHz audiogram)	> 20 dB hearing loss at ≤ 4 kHz	> 20 dB hearing loss at > 4 kHz	> 20 dB hearing loss at ≥ 3 kHz in one ear with additional speech language related services indicated (where available) <u>OR</u> Hearing loss sufficient to indicate therapeutic intervention, including hearing aids	Audiologic indication for cochlear implant and additional speech-language related services indicated (where available)
**Tinnitus**	Symptoms causing no or minimal interference with usual social & functional activities with intervention not indicated	Symptoms causing greater than minimal interference with usual social & functional activities with intervention indicated	Symptoms causing inability to perform usual social & functional activities	NA
**Uveitis**	No symptoms <u>AND</u> Detectable on examination	Anterior uveitis with symptoms <u>OR</u> Medical intervention indicated	Posterior or pan-uveitis <u>OR</u> Operative intervention indicated	Disabling visual loss in affected eye(s)
**Vertigo**	Vertigo causing no or minimal interference with usual social & functional activities	Vertigo causing greater than minimal interference with usual social & functional activities	Vertigo causing inability to perform usual social & functional activities	Disabling vertigo causing inability to perform basic self-care functions
**Visual Changes** (assessed from baseline)	Visual changes causing no or minimal interference with usual social & functional activities	Visual changes causing greater than minimal interference with usual social & functional activities	Visual changes causing inability to perform usual social & functional activities	Disabling visual loss in affected eye(s)

Systemic

PARAMETER	GRADE 1 MILD	GRADE 2 MODERATE	GRADE 3 SEVERE	GRADE 4 POTENTIALLY LIFE-THREATENING
**Acute Allergic Reaction**	Localized urticaria (wheals) with no medical intervention indicated	Localized urticaria with intervention indicated OR Mild angioedema with no intervention indicated	Generalized urticaria OR Angioedema with intervention indicated OR Symptoms of mild bronchospasm	Acute anaphylaxis OR Life-threatening bronchospasm OR Laryngeal edema
**Chills**	Symptoms causing no or minimal interference with usual social & functional activities	Symptoms causing greater than minimal interference with usual social & functional activities	Symptoms causing inability to perform usual social & functional activities	NA
**Cytokine Release Syndrome**[8]	Mild signs and symptoms AND Therapy (i.e., antibody infusion) interruption not indicated	Therapy (i.e., antibody infusion) interruption indicated AND Responds promptly to symptomatic treatment OR Prophylactic medications indicated for ≤ 24 hours	Prolonged severe signs and symptoms OR Recurrence of symptoms following initial improvement	Life-threatening consequences (e.g., requiring pressor or ventilator support)
**Fatigue or Malaise** *Report only one*	Symptoms causing no or minimal interference with usual social & functional activities	Symptoms causing greater than minimal interference with usual social & functional activities	Symptoms causing inability to perform usual social & functional activities	Incapacitating symptoms of fatigue or malaise causing inability to perform basic self-care functions
**Fever** (non-axillary temperatures only)	38.0 to < 38.6°C or 100.4 to < 101.5°F	≥ 38.6 to < 39.3°C or ≥ 101.5 to < 102.7°F	≥ 39.3 to < 40.0°C or ≥ 102.7 to < 104.0°F	≥ 40.0°C or ≥ 104.0°F
**Pain**[9] (not associated with study agent injections and not specified elsewhere) *Specify location*	Pain causing no or minimal interference with usual social & functional activities	Pain causing greater than minimal interference with usual social & functional activities	Pain causing inability to perform usual social & functional activities	Disabling pain causing inability to perform basic self-care functions OR Hospitalization indicated

[8] Definition: A disorder characterized by nausea, headache, tachycardia, hypotension, rash, and/or shortness of breath.
[9] For pain associated with injections or infusions, see the *Site Reactions to Injections and Infusions* section (page 23).

Systemic

**Serum Sickness**[10]	Mild signs and symptoms	Moderate signs and symptoms AND Intervention indicated (e.g., antihistamines)	Severe signs and symptoms AND Higher level intervention indicated (e.g., steroids or IV fluids)	Life-threatening consequences (e.g., requiring pressor or ventilator support)
**Underweight**[11] *> 5 to 19 years of age*	WHO BMI z-score < -1 to -2	WHO BMI z-score < -2 to -3	WHO BMI z-score < -3	WHO BMI z-score < -3 with life-threatening consequences
2 to 5 years of age	WHO Weight-for-height z-score < -1 to -2	WHO Weight-for-height z-score < -2 to -3	WHO Weight-for-height z-score < -3	WHO Weight-for-height z-score < -3 with life-threatening consequences
< 2 years of age	WHO Weight-for-length z-score < -1 to -2	WHO Weight-for-length z-score < -2 to -3	WHO Weight-for-length z-score < -3	WHO Weight-for-length z-score < -3 with life-threatening consequences
**Unintentional Weight Loss** (excludes postpartum weight loss)	NA	5 to < 9% loss in body weight from baseline	≥ 9 to < 20% loss in body weight from baseline	≥ 20% loss in body weight from baseline OR Aggressive intervention indicated (e.g., tube feeding, total parenteral nutrition)

[10] Definition: A disorder characterized by fever, arthralgia, myalgia, skin eruptions, lymphadenopathy, marked discomfort, and/or dyspnea.
[11] WHO reference tables may be accessed by clicking the desired age range or by accessing the following URLs: http://www.who.int/growthref/who2007_bmi_for_age/en/ for participants > 5 to 19 years of age and http://www.who.int/childgrowth/standards/chart_catalogue/en/ for those ≤ 5 years of age.

Urinary

PARAMETER	GRADE 1 MILD	GRADE 2 MODERATE	GRADE 3 SEVERE	GRADE 4 POTENTIALLY LIFE-THREATENING
**Urinary Tract Obstruction**	NA	Signs or symptoms of urinary tract obstruction without hydronephrosis or renal dysfunction	Signs or symptoms of urinary tract obstruction with hydronephrosis or renal dysfunction	Obstruction causing life-threatening consequences

Site Reactions to Injections and Infusions

PARAMETER	GRADE 1 MILD	GRADE 2 MODERATE	GRADE 3 SEVERE	GRADE 4 POTENTIALLY LIFE-THREATENING
**Injection Site Pain or Tenderness**<br>*Report only one*	Pain or tenderness causing no or minimal limitation of use of limb	Pain or tenderness causing greater than minimal limitation of use of limb	Pain or tenderness causing inability to perform usual social & functional activities	Pain or tenderness causing inability to perform basic self-care function <u>OR</u> Hospitalization indicated
**Injection Site Erythema or Redness**[12]<br>*Report only one*<br>*> 15 years of age*	2.5 to < 5 cm in diameter <u>OR</u> 6.25 to < 25 cm^2 surface area <u>AND</u> Symptoms causing no or minimal interference with usual social & functional activities	≥ 5 to < 10 cm in diameter <u>OR</u> ≥ 25 to < 100 cm^2 surface area <u>OR</u> Symptoms causing greater than minimal interference with usual social & functional activities	≥ 10 cm in diameter <u>OR</u> ≥ 100 cm^2 surface area <u>OR</u> Ulceration <u>OR</u> Secondary infection <u>OR</u> Phlebitis <u>OR</u> Sterile abscess <u>OR</u> Drainage <u>OR</u> Symptoms causing inability to perform usual social & functional activities	Potentially life-threatening consequences (e.g., abscess, exfoliative dermatitis, necrosis involving dermis or deeper tissue)
≤ 15 years of age	≤ 2.5 cm in diameter	> 2.5 cm in diameter with < 50% surface area of the extremity segment involved (e.g., upper arm or thigh)	≥ 50% surface area of the extremity segment involved (e.g., upper arm or thigh) <u>OR</u> Ulceration <u>OR</u> Secondary infection <u>OR</u> Phlebitis <u>OR</u> Sterile abscess <u>OR</u> Drainage	Potentially life-threatening consequences (e.g., abscess, exfoliative dermatitis, necrosis involving dermis or deeper tissue)
**Injection Site Induration or Swelling**<br>*Report only one*<br>*> 15 years of age*	Same as for **Injection Site Erythema or Redness,** *> 15 years of age*	Same as for **Injection Site Erythema or Redness,** *> 15 years of age*	Same as for **Injection Site Erythema or Redness,** *> 15 years of age*	Same as for **Injection Site Erythema or Redness,** *> 15 years of age*
≤ 15 years of age	Same as for **Injection Site Erythema or Redness,** *≤ 15 years of age*	Same as for **Injection Site Erythema or Redness,** *≤ 15 years of age*	Same as for **Injection Site Erythema or Redness,** *≤ 15 years of age*	Same as for **Injection Site Erythema or Redness,** *≤ 15 years of age*
**Injection Site Pruritus**	Itching localized to the injection site that is relieved spontaneously or in < 48 hours of treatment	Itching beyond the injection site that is not generalized <u>OR</u> Itching localized to the injection site requiring ≥ 48 hours treatment	Generalized itching causing inability to perform usual social & functional activities	NA

[12] Injection Site Erythema or Redness should be evaluated and graded using the greatest single diameter or measured surface area.

*Laboratory Values**
Chemistries

PARAMETER	GRADE 1 MILD	GRADE 2 MODERATE	GRADE 3 SEVERE	GRADE 4 POTENTIALLY LIFE-THREATENING
**Acidosis**	NA	pH ≥ 7.3 to < LLN	pH < 7.3 without life-threatening consequences	pH < 7.3 with life-threatening consequences
**Albumin, Low** (g/dL; *g/L*)	3.0 to < LLN<br>*30 to < LLN*	≥ 2.0 to < 3.0<br>*≥ 20 to < 30*	< 2.0<br>*< 20*	NA
**Alkaline Phosphatase, High**	1.25 to < 2.5 x ULN	2.5 to < 5.0 x ULN	5.0 to < 10.0 x ULN	≥ 10.0 x ULN
**Alkalosis**	NA	pH > ULN to ≤ 7.5	pH > 7.5 without life-threatening consequences	pH > 7.5 with life-threatening consequences
**ALT or SGPT, High**<br>*Report only one*	1.25 to < 2.5 x ULN	2.5 to < 5.0 x ULN	5.0 to < 10.0 x ULN	≥ 10.0 x ULN
**Amylase (Pancreatic) or Amylase (Total), High**<br>*Report only one*	1.1 to < 1.5 x ULN	1.5 to < 3.0 x ULN	3.0 to < 5.0 x ULN	≥ 5.0 x ULN
**AST or SGOT, High**<br>*Report only one*	1.25 to < 2.5 x ULN	2.5 to < 5.0 x ULN	5.0 to < 10.0 x ULN	≥ 10.0 x ULN
**Bicarbonate, Low** (mEq/L; *mmol/L*)	16.0 to < LLN<br>*16.0 to < LLN*	11.0 to < 16.0<br>*11.0 to < 16.0*	8.0 to < 11.0<br>*8.0 to < 11.0*	< 8.0<br>*< 8.0*
**Bilirubin**<br>***Direct Bilirubin[13], High***<br>*> 28 days of age*	NA	NA	> ULN with other signs and symptoms of hepatotoxicity.	> ULN with life-threatening consequences (e.g., signs and symptoms of liver failure)
≤ 28 days of age	ULN to ≤ 1 mg/dL	> 1 to ≤ 1.5 mg/dL	> 1.5 to ≤ 2 mg/dL	> 2 mg/dL
Total Bilirubin, High<br>*> 28 days of age*	1.1 to < 1.6 x ULN	1.6 to < 2.6 x ULN	2.6 to < 5.0 x ULN	≥ 5.0 x ULN
≤ 28 days of age	See Appendix A. Total Bilirubin for Term and Preterm Neonates	See Appendix A. Total Bilirubin for Term and Preterm Neonates	See Appendix A. Total Bilirubin for Term and Preterm Neonates	See Appendix A. Total Bilirubin for Term and Preterm Neonates

*Reminder: An asymptomatic abnormal laboratory finding without an accompanying AE should not be reported to DAIDS in an expedited time frame unless it meets protocol-specific reporting requirements.

[13] Direct bilirubin > 1.5 mg/dL in a participant < 28 days of age should be graded as grade 2, if < 10% of the total bilirubin.

Chemistries

PARAMETER	GRADE 1 MILD	GRADE 2 MODERATE	GRADE 3 SEVERE	GRADE 4 POTENTIALLY LIFE-THREATENING
**Calcium, High** (mg/dL; *mmol/L*) *≥ 7 days of age*	10.6 to < 11.5<br>*2.65 to < 2.88*	11.5 to < 12.5<br>*2.88 to < 3.13*	12.5 to < 13.5<br>*3.13 to < 3.38*	≥ 13.5<br>*≥ 3.38*
< 7 days of age	11.5 to < 12.4<br>*2.88 to < 3.10*	12.4 to < 12.9<br>*3.10 to < 3.23*	12.9 to < 13.5<br>*3.23 to < 3.38*	≥ 13.5<br>*≥ 3.38*
**Calcium (Ionized), High** (mg/dL; *mmol/L*)	> ULN to < 6.0<br>*> ULN to < 1.5*	6.0 to < 6.4<br>*1.5 to < 1.6*	6.4 to < 7.2<br>*1.6 to < 1.8*	≥ 7.2<br>*≥ 1.8*
**Calcium, Low** (mg/dL; *mmol/L*) *≥ 7 days of age*	7.8 to < 8.4<br>*1.95 to < 2.10*	7.0 to < 7.8<br>*1.75 to < 1.95*	6.1 to < 7.0<br>*1.53 to < 1.75*	< 6.1<br>*< 1.53*
< 7 days of age	6.5 to < 7.5<br>*1.63 to < 1.88*	6.0 to < 6.5<br>*1.50 to < 1.63*	5.50 to < 6.0<br>*1.38 to < 1.50*	< 5.50<br>*< 1.38*
**Calcium (Ionized), Low** (mg/dL; *mmol/L*)	< LLN to 4.0<br>*< LLN to 1.0*	3.6 to < 4.0<br>*0.9 to < 1.0*	3.2 to < 3.6<br>*0.8 to < 0.9*	< 3.2<br>*< 0.8*
**Cardiac Troponin I, High**	NA	NA	NA	Levels consistent with myocardial infarction or unstable angina as defined by the local laboratory
**Creatine Kinase, High**	3 to < 6 x ULN	6 to < 10x ULN	10 to < 20 x ULN	≥ 20 x ULN
**Creatinine, High** **Report only one*	1.1 to 1.3 x ULN	> 1.3 to 1.8 x ULN <u>OR</u> Increase to 1.3 to < 1.5 x participant's baseline	> 1.8 to < 3.5 x ULN <u>OR</u> Increase to 1.5 to < 2.0 x participant's baseline	≥ 3.5 x ULN <u>OR</u> Increase of ≥ 2.0 x participant's baseline
**Creatinine Clearance[14] or eGFR, Low** **Report only one*	NA	< 90 to 60 ml/min or ml/min/1.73 m^2 <u>OR</u> 10 to < 30% decrease from participant's baseline	< 60 to 30 ml/min or ml/min/1.73 m^2 <u>OR</u> 30 to < 50% decrease from participant's baseline	< 30 ml/min or ml/min/1.73 m^2 <u>OR</u> ≥ 50% decrease from participant's baseline or dialysis needed
**Glucose** (mg/dL; *mmol/L*) ***Fasting, High***	110 to 125<br>*6.11 to < 6.95*	> 125 to 250<br>*6.95 to < 13.89*	> 250 to 500<br>*13.89 to < 27.75*	≥ 500<br>*≥ 27.75*
Nonfasting, High	116 to 160<br>*6.44 to < 8.89*	> 160 to 250<br>*8.89 to < 13.89*	> 250 to 500<br>*13.89 to < 27.75*	≥ 500<br>*≥ 27.75*

14 Use the applicable formula (i.e., Cockcroft-Gault in mL/min or Schwartz, MDRD, CKD-Epi in mL/min/1.73m2). Sites should choose the method defined in their study and when not specified, use the method most relevant to the study population.

*Reminder: Choose the method that selects for the higher grade.

Chemistries

PARAMETER	GRADE 1 MILD	GRADE 2 MODERATE	GRADE 3 SEVERE	GRADE 4 POTENTIALLY LIFE-THREATENING
**Glucose, Low** (mg/dL; *mmol/L*) *≥ 1 month of age*	55 to 64<br>*3.05 to <3.55*	40 to < 55<br>*2.22 to < 3.05*	30 to < 40<br>*1.67 to < 2.22*	< 30<br>*< 1.67*
< 1 month of age	50 to 54<br>*2.78 to < 3.00*	40 to < 50<br>*2.22 to < 2.78*	30 to < 40<br>*1.67 to < 2.22*	< 30<br>*< 1.67*
**Lactate, High**	ULN to < 2.0 x ULN without acidosis	≥ 2.0 x ULN without acidosis	Increased lactate with pH < 7.3 without life-threatening consequences	Increased lactate with pH < 7.3 with life-threatening consequences
**Lipase, High**	1.1 to < 1.5 x ULN	1.5 to < 3.0 x ULN	3.0 to < 5.0 x ULN	≥ 5.0 x ULN
**Lipid Disorders** (mg/dL; *mmol/L*) ***Cholesterol, Fasting, High*** *≥ 18 years of age*	200 to < 240<br>*5.18 to < 6.19*	240 to < 300<br>*6.19 to < 7.77*	≥ 300<br>*≥ 7.77*	NA
< 18 years of age	170 to < 200<br>*4.40 to < 5.15*	200 to < 300<br>*5.15 to < 7.77*	≥ 300<br>*≥ 7.77*	NA
LDL, Fasting, High *≥ 18 years of age*	130 to < 160<br>*3.37 to < 4.12*	160 to < 190<br>*4.12 to < 4.90*	≥ 190<br>*≥ 4.90*	NA
> 2 to < 18 years of age	110 to < 130<br>*2.85 to < 3.34*	130 to < 190<br>*3.34 to < 4.90*	≥ 190<br>*≥ 4.90*	NA
Triglycerides, Fasting, High	150 to 300<br>*1.71 to 3.42*	>300 to 500<br>*>3.42 to 5.7*	>500 to < 1,000<br>*>5.7 to 11.4*	> 1,000<br>*> 11.4*
**Magnesium[15], Low** (mEq/L; *mmol/L*)	1.2 to < 1.4<br>*0.60 to < 0.70*	0.9 to < 1.2<br>*0.45 to < 0.60*	0.6 to < 0.9<br>*0.30 to < 0.45*	< 0.6<br>*< 0.30*
**Phosphate, Low** (mg/dL; *mmol/L*) *> 14 years of age*	2.0 to < LLN<br>*0.65 to < LLN*	1.4 to < 2.0<br>*0.45 to < 0.65*	1.0 to < 1.4<br>*0.32 to < 0.45*	< 1.0<br>*< 0.32*
1 to 14 years of age	3.0 to < 3.5<br>*0.97 to < 1.13*	2.5 to < 3.0<br>*0.81 to < 0.97*	1.5 to < 2.5<br>*0.48 to < 0.81*	< 1.5<br>*< 0.48*
< 1 year of age	3.5 to < 4.5<br>*1.13 to < 1.45*	2.5 to < 3.5<br>*0.81 to < 1.13*	1.5 to < 2.5<br>*0.48 to < 0.81*	< 1.5<br>*< 0.48*
**Potassium, High** (mEq/L; *mmol/L*)	5.6 to < 6.0<br>*5.6 to < 6.0*	6.0 to < 6.5<br>*6.0 to < 6.5*	6.5 to < 7.0<br>*6.5 to < 7.0*	≥ 7.0<br>*≥ 7.0*
**Potassium, Low** (mEq/L; *mmol/L*)	3.0 to < 3.4<br>*3.0 to < 3.4*	2.5 to < 3.0<br>*2.5 to < 3.0*	2.0 to < 2.5<br>*2.0 to < 2.5*	< 2.0<br>*< 2.0*

[15] To convert a magnesium value from mg/dL to mmol/L, laboratories should multiply by 0.4114.

Chemistries

PARAMETER	GRADE 1 MILD	GRADE 2 MODERATE	GRADE 3 SEVERE	GRADE 4 POTENTIALLY LIFE-THREATENING
**Sodium, High** (mEq/L; *mmol/L*)	146 to < 150<br>*146 to < 150*	150 to < 154<br>*150 to < 154*	154 to < 160<br>*154 to < 160*	≥ 160<br>*≥ 160*
**Sodium, Low** (mEq/L; *mmol/L*)	130 to < 135<br>*130 to < 135*	125 to < 130<br>*125 to < 130*	121 to < 125<br>*121 to < 125*	≤ 120<br>*≤ 120*
**Uric Acid, High** (mg/dL; *mmol/L*)	7.5 to < 10.0<br>*0.45 to < 0.59*	10.0 to < 12.0<br>*0.59 to < 0.71*	12.0 to < 15.0<br>*0.71 to < 0.89*	≥ 15.0<br>*≥ 0.89*

Hematology

PARAMETER	GRADE 1 MILD	GRADE 2 MODERATE	GRADE 3 SEVERE	GRADE 4 POTENTIALLY LIFE-THREATENING
**Absolute CD4+ Count, Low** (cell/mm^3; *cells/L*)<br>*> 5 years of age (not HIV infected)*	300 to < 400<br>*300 to < 400*	200 to < 300<br>*200 to < 300*	100 to < 200<br>*100 to < 200*	< 100<br>*< 100*
**Absolute Lymphocyte Count, Low** (cell/mm^3; *cells/L*)<br>*> 5 years of age (not HIV infected)*	600 to < 650<br>*0.600 x 10^9 to < 0.650 x 10^9*	500 to < 600<br>*0.500 x 10^9 to < 0.600 x 10^9*	350 to < 500<br>*0.350 x 10^9 to < 0.500 x 10^9*	< 350<br>*< 0.350 x 10^9*
**Absolute Neutrophil Count (ANC), Low** (cells/mm^3; *cells/L*)<br>*> 7 days of age*	800 to 1,000<br>*0.800 x 10^9 to 1.000 x 10^9*	600 to 799<br>*0.600 x 10^9 to 0.799 x 10^9*	400 to 599<br>*0.400 x 10^9 to 0.599 x 10^9*	< 400<br>*< 0.400 x 10^9*
2 to 7 days of age	1,250 to 1,500<br>*1.250 x 10^9 to 1.500 x 10^9*	1,000 to 1,249<br>*1.000 x 10^9 to 1.249 x 10^9*	750 to 999<br>*0.750 x 10^9 to 0.999 x 10^9*	< 750<br>*< 0.750 x 10^9*
≤ 1 day of age	4,000 to 5,000<br>*4.000 x 10^9 to 5.000 x 10^9*	3,000 to 3,999<br>*3.000 x 10^9 to 3.999 x 10^9*	1,500 to 2,999<br>*1.500 x 10^9 to 2.999 x 10^9*	< 1,500<br>*< 1.500 x 10^9*
**Fibrinogen, Decreased** (mg/dL; *g/L*)	100 to < 200<br>*1.00 to < 2.00*<br>OR<br>0.75 to < 1.00 x LLN	75 to < 100<br>*0.75 to < 1.00*<br>OR<br>≥ 0.50 to < 0.75 x LLN	50 to < 75<br>*0.50 to < 0.75*<br>OR<br>0.25 to < 0.50 x LLN	< 50<br>*< 0.50*<br>OR<br>< 0.25 x LLN<br>OR Associated with gross bleeding
**Hemoglobin[16], Low** (g/dL; *mmol/L*)[17]<br>*≥ 13 years of age (male only)*	10.0 to 10.9<br>*6.19 to 6.76*	9.0 to < 10.0<br>*5.57 to < 6.19*	7.0 to < 9.0<br>*4.34 to < 5.57*	< 7.0<br>*< 4.34*
≥ 13 years of age (female only)	9.5 to 10.4<br>*5.88 to 6.48*	8.5 to < 9.5<br>*5.25 to < 5.88*	6.5 to < 8.5<br>*4.03 to < 5.25*	< 6.5<br>*< 4.03*

[16] Male and female sex are defined as sex at birth. For transgender participants ≥13 years of age who have been on hormone therapy for more than 6 consecutive months, grade hemoglobin based on the gender with which they identify (i.e., a transgender female should be graded using the female sex at birth hemoglobin laboratory values).

[17] The most commonly used conversion factor to convert g/dL to mmol/L is 0.6206. For grading hemoglobin results obtained by an analytic method with a conversion factor other than 0.6206, the result must be converted to g/dL using appropriate conversion factor for the particular laboratory.

Hematology

PARAMETER	GRADE 1 MILD	GRADE 2 MODERATE	GRADE 3 SEVERE	GRADE 4 POTENTIALLY LIFE-THREATENING
57 days of age to < 13 years of age (male and female)	9.5 to 10.4<br>*5.88 to 6.48*	8.5 to < 9.5<br>*5.25 to < 5.88*	6.5 to < 8.5<br>*4.03 to < 5.25*	< 6.5<br>*< 4.03*
36 to 56 days of age (male and female)	8.5 to 9.6<br>*5.26 to 5.99*	7.0 to < 8.5<br>*4.32 to < 5.26*	6.0 to < 7.0<br>*3.72 to < 4.32*	< 6.0<br>*< 3.72*
22 to 35 days of age (male and female)	9.5 to 11.0<br>*5.88 to 6.86*	8.0 to < 9.5<br>*4.94 to < 5.88*	6.7 to < 8.0<br>*4.15 to < 4.94*	< 6.7<br>*< 4.15*
8 to ≤ 21 days of age (male and female)	11.0 to 13.0<br>*6.81 to 8.10*	9.0 to < 11.0<br>*5.57 to < 6.81*	8.0 to < 9.0<br>*4.96 to < 5.57*	< 8.0<br>***< 4.96***
≤ 7 days of age (male and female)	13.0 to 14.0<br>*8.05 to 8.72*	10.0 to < 13.0<br>*6.19 to < 8.05*	9.0 to < 10.0<br>*5.59 to < 6.19*	< 9.0<br>*< 5.59*
**INR, High** (not on anticoagulation therapy)	1.1 to < 1.5 x ULN	1.5 to < 2.0 x ULN	2.0 to < 3.0 x ULN	≥ 3.0 x ULN
**Methemoglobin** (% hemoglobin)	5.0 to < 10.0%	10.0 to < 15.0%	15.0 to < 20.0%	≥ 20.0%
**PTT, High** (not on anticoagulation therapy)	1.1 to < 1.66 x ULN	1.66 to < 2.33 x ULN	2.33 to < 3.00 x ULN	≥ 3.00 x ULN
**Platelets, Decreased** (cells/mm^3; *cells/L*)	100,000 to < 125,000<br>*100.000 x 10^9 to < 125.000 x 10^9*	50,000 to < 100,000<br>*50.000 x 10^9 to < 100.000 x 10^9*	25,000 to < 50,000<br>*25.000 x 10^9 to < 50.000 x 10^9*	< 25,000<br>*< 25.000 x 10^9*
**PT, High** (not on anticoagulation therapy	1.1 to < 1.25 x ULN	1.25 to < 1.50 x ULN	1.50 to < 3.00 x ULN	≥ 3.00 x ULN
**WBC, Decreased** (cells/mm^3; *cells/L*)<br>*> 7 days of age*	2,000 to 2,499<br>*2.000 x 10^9 to 2.499 x 10^9*	1,500 to 1,999<br>*1.500 x 10^9 to 1.999 x 10^9*	1,000 to 1,499<br>*1.000 x 10^9 to 1.499 x 10^9*	< 1,000<br>*< 1.000 x 10^9*
≤ 7 days of age	5,500 to 6,999<br>*5.500 x 10^9 to 6.999 x 10^9*	4,000 to 5,499<br>*4.000 x 10^9 to 5.499 x 10^9*	2,500 to 3,999<br>*2.500 x 10^9 to 3.999 x 10^9*	< 2,500<br>*< 2.500 x 10^9*

Urinalysis

PARAMETER	GRADE 1 MILD	GRADE 2 MODERATE	GRADE 3 SEVERE	GRADE 4 POTENTIALLY LIFE-THREATENING
**Glycosuria** (random collection tested by dipstick)	Trace to 1+ or ≤ 250 mg	2+ or > 250 to ≤ 500 mg	> 2+ or > 500 mg	NA
**Hematuria** (not to be reported based on dipstick findings or on blood believed to be of menstrual origin)	6 to < 10 RBCs per high power field	≥ 10 RBCs per high power field	Gross, with or without clots OR With RBC casts OR Intervention indicated	Life-threatening consequences
**Proteinuria** (random collection tested by dipstick)	1+	2+	3+ or higher	NA

Appendix A.
Total Bilirubin Table for Term and Preterm Neonates

PARAMETER	GRADE 1<br>MILD	GRADE 2<br>MODERATE	GRADE 3<br>SEVERE	GRADE 4<br>POTENTIALLY LIFE-THREATENING
**Total Bilirubin**[18]**, High**<br>(mg/dL; *µmol/L*)[19]				
Term Neonate[20]<br>*< 24 hours of age*	4 to < 7<br>*68.4 to < 119.7*	7 to < 10<br>*119.7 to < 171*	10 to < 17<br>*171* to *< 290.7*	≥ 17<br>*≥ 290.7*
24 to < 48 hours of age	5 to < 8<br>*85.5 to < 136.8*	8 to < 12<br>*136.8 to < 205.2*	12 to < 19<br>*205.2* to *< 324.9*	≥ 19<br>*≥ 324.9*
48 to < 72 hours of age	8.5 to < 13<br>*145.35 to < 222.3*	13 to < 15<br>*222.3 to < 256.5*	15 to < 22<br>*256.5* to *< 376.2*	≥ 22<br>*≥ 376.2*
72 hours to < 7 days of age	11 to < 16<br>*188.1 to < 273.6*	16 to < 18<br>*273.6 to < 307.8*	18 to < 24<br>*307.8* to *< 410.4*	≥ 24<br>*≥ 410.4*
7 to 28 days of age (breast feeding)	5 to < 10<br>*85.5 to < 171*	10 to < 20<br>*171 to < 342*	20 to < 25<br>*342 to < 427.5*	≥ 25<br>*≥ 427.5*
7 to 28 days of age (not breast feeding)	1.1 to < 1.6 x ULN	1.6 to < 2.6 x ULN	2.6 to < 5.0 x ULN	≥ 5.0 x ULN
Preterm Neonate[20]<br>*35 to < 37 weeks gestational age*	Same as for ***Total Bilirubin, High, Term Neonate*** (based on days of age).	Same as for ***Total Bilirubin, High, Term Neonate*** (based on days of age).	Same as for ***Total Bilirubin, High, Term Neonate*** (based on days of age).	Same as for ***Total Bilirubin, High, Term Neonate*** (based on days of age).
32 to < 35 weeks gestational age and < 7 days of age	NA	NA	10 to < 14<br>*171 to < 239.4*	≥ 14<br>*≥ 239.4*
28 to < 32 weeks gestational age and < 7 days of age	NA	NA	6 to < 10<br>*102.6 to < 171*	≥ 10<br>*≥ 171*
< 28 weeks gestational age and < 7 days of age	NA	NA	5 to < 8<br>*85.5 to < 136.8*	≥ 8<br>*≥ 136.8*
7 to 28 days of age (breast feeding)	5 to < 10<br>*85.5 to < 171*	10 to < 20<br>*171 to < 342*	20 to < 25<br>*342 to < 427.5*	≥ 25<br>*≥ 427.5*
7 to 28 days of age (not breast feeding)	1.1 to < 1.6 x ULN	1.6 to < 2.6 x ULN	2.6 to < 5.0 x ULN	≥ 5.0 x ULN

[18] Severity grading for total bilirubin in neonates is complex because of rapidly changing total bilirubin normal ranges in the first week of life followed by the benign phenomenon of breast milk jaundice after the first week of life. Severity grading in this appendix corresponds approximately to cut-offs for indications for phototherapy at grade 3 and for exchange transfusion at grade 4.

[19] A laboratory value of 1 mg/dL is equivalent to 17.1 µmol/L.

[20] Definitions: Term is defined as ≥ 37 weeks gestational age; near-term, as ≥ 35 weeks gestational age; preterm, as < 35 weeks gestational age; and neonate, as 0 to 28 days of age.

Appendix IV

Declaration of Helsinki

WMA Declaration of Helsinki - Ethical Principles for Medical Research Involving Human Subjects

Adopted by the 18th WMA General Assembly, Helsinki, Finland, June 1964
and amended by the:
29th WMA General Assembly, Tokyo, Japan, October 1975
35th WMA General Assembly, Venice, Italy, October 1983
41st WMA General Assembly, Hong Kong, September 1989
48th WMA General Assembly, Somerset West, Republic of South Africa, October 1996
52nd WMA General Assembly, Edinburgh, Scotland, October 2000
53rd WMA General Assembly, Washington DC, USA, October 2002 (Note of Clarification added)
55th WMA General Assembly, Tokyo, Japan, October 2004 (Note of Clarification added)
59th WMA General Assembly, Seoul, Republic of Korea, October 2008
64th WMA General Assembly, Fortaleza, Brazil, October 2013

Preamble

1. The World Medical Association (WMA) has developed the Declaration of Helsinki as a statement of ethical principles for medical research involving human subjects, including research on identifiable human material and data.

The Declaration is intended to be read as a whole and each of its constituent paragraphs should be applied with consideration of all other relevant paragraphs.

2. Consistent with the mandate of the WMA, the Declaration is addressed primarily to physicians. The WMA encourages others who are involved in medical research involving human subjects to adopt these principles.

General Principles

3. The Declaration of Geneva of the WMA binds the physician with the words,

“The health of my patient will be my first consideration,” and the International Code of Medical Ethics declares that, “A physician shall act in the patient's best interest when providing medical care.”

4. It is the duty of the physician to promote and safeguard the health, well-being and rights of patients, including those who are involved in medical research. The physician's knowledge and conscience are dedicated to the fulfilment of this duty.

5. Medical progress is based on research that ultimately must include studies involving human subjects.

6. The primary purpose of medical research involving human subjects is to understand the causes, development and effects of diseases and improve preventive, diagnostic and therapeutic interventions (methods, procedures and treatments). Even the best proven interventions must be evaluated continually through research for their safety, effectiveness, efficiency, accessibility and quality.

7. Medical research is subject to ethical standards that promote and ensure respect for all human subjects and protect their health and rights.

8. While the primary purpose of medical research is to generate new knowledge, this goal can never take precedence over the rights and interests of individual research subjects.

9. It is the duty of physicians who are involved in medical research to protect the life, health, dignity, integrity, right to self-determination, privacy, and confidentiality of personal information of research subjects. The responsibility for the protection of research subjects must always rest with the physician or other health care professionals and never with the research subjects, even though they have given consent.

10. Physicians must consider the ethical, legal and regulatory norms and standards for research involving human subjects in their own countries as well as applicable international norms and standards. No national or international ethical, legal or regulatory requirement should reduce or eliminate any of the protections for research subjects set forth in this Declaration.

11. Medical research should be conducted in a manner that minimises possible harm to the environment.

12. Medical research involving human subjects must be conducted only by

individuals with the appropriate ethics and scientific education, training and qualifications. Research on patients or healthy volunteers requires the supervision of a competent and appropriately qualified physician or other health care professional.

13. Groups that are underrepresented in medical research should be provided appropriate access to participation in research.

14. Physicians who combine medical research with medical care should involve their patients in research only to the extent that this is justified by its potential preventive, diagnostic or therapeutic value and if the physician has good reason to believe that participation in the research study will not adversely affect the health of the patients who serve as research subjects.

15. Appropriate compensation and treatment for subjects who are harmed as a result of participating in research must be ensured.

Risks, Burdens and Benefits

16. In medical practice and in medical research, most interventions involve risks and burdens.

Medical research involving human subjects may only be conducted if the importance of the objective outweighs the risks and burdens to the research subjects.

17. All medical research involving human subjects must be preceded by careful assessment of predictable risks and burdens to the individuals and groups involved in the research in comparison with foreseeable benefits to them and to other individuals or groups affected by the condition under investigation.

Measures to minimise the risks must be implemented. The risks must be continuously monitored, assessed and documented by the researcher.

18. Physicians may not be involved in a research study involving human subjects unless they are confident that the risks have been adequately assessed and can be satisfactorily managed.

When the risks are found to outweigh the potential benefits or when there is conclusive proof of definitive outcomes, physicians must assess whether to continue, modify or immediately stop the study.

Vulnerable Groups and Individuals

19. Some groups and individuals are particularly vulnerable and may have an increased likelihood of being wronged or of incurring additional harm.

All vulnerable groups and individuals should receive specifically considered protection.

20. Medical research with a vulnerable group is only justified if the research is responsive to the health needs or priorities of this group and the research cannot be carried out in a non-vulnerable group. In addition, this group should stand to benefit from the knowledge, practices or interventions that result from the research.

Scientific Requirements and Research Protocols

21. Medical research involving human subjects must conform to generally accepted scientific principles, be based on a thorough knowledge of the scientific literature, other relevant sources of information, and adequate laboratory and, as appropriate, animal experimentation. The welfare of animals used for research must be respected.

22. The design and performance of each research study involving human subjects must be clearly described and justified in a research protocol.

The protocol should contain a statement of the ethical considerations involved and should indicate how the principles in this Declaration have been addressed. The protocol should include information regarding funding, sponsors, institutional affiliations, potential conflicts of interest, incentives for subjects and information regarding provisions for treating and/or compensating subjects who are harmed as a consequence of participation in the research study.

In clinical trials, the protocol must also describe appropriate arrangements for post-trial provisions.

Research Ethics Committees

23. The research protocol must be submitted for consideration, comment, guidance and approval to the concerned research ethics committee before the study begins. This committee must be transparent in its functioning, must be independent of the researcher, the sponsor and any other undue influence and must be duly qualified. It must take into consideration the laws and regulations of the country or countries in which the research is to be performed as well as applicable international norms and

standards but these must not be allowed to reduce or eliminate any of the protections for research subjects set forth in this Declaration.

The committee must have the right to monitor ongoing studies. The researcher must provide monitoring information to the committee, especially information about any serious adverse events. No amendment to the protocol may be made without consideration and approval by the committee. After the end of the study, the researchers must submit a final report to the committee containing a summary of the study's findings and conclusions.

Privacy and Confidentiality

24. Every precaution must be taken to protect the privacy of research subjects and the confidentiality of their personal information.

Informed Consent

25. Participation by individuals capable of giving informed consent as subjects in medical research must be voluntary. Although it may be appropriate to consult family members or community leaders, no individual capable of giving informed consent may be enrolled in a research study unless he or she freely agrees.

26. In medical research involving human subjects capable of giving informed consent, each potential subject must be adequately informed of the aims, methods, sources of funding, any possible conflicts of interest, institutional affiliations of the researcher, the anticipated benefits and potential risks of the study and the discomfort it may entail, post-study provisions and any other relevant aspects of the study. The potential subject must be informed of the right to refuse to participate in the study or to withdraw consent to participate at any time without reprisal. Special attention should be given to the specific information needs of individual potential subjects as well as to the methods used to deliver the information.

After ensuring that the potential subject has understood the information, the physician or another appropriately qualified individual must then seek the potential subject's freely-given informed consent, preferably in writing. If the consent cannot be expressed in writing, the non-written consent must be formally documented and witnessed.

All medical research subjects should be given the option of being informed about the general outcome and results of the study.

27. When seeking informed consent for participation in a research study the physician must be particularly cautious if the potential subject is in a dependent relationship with the physician or may consent under duress. In such situations the informed consent must be sought by an appropriately qualified individual who is completely independent of this relationship.

28. For a potential research subject who is incapable of giving informed consent, the physician must seek informed consent from the legally authorised representative. These individuals must not be included in a research study that has no likelihood of benefit for them unless it is intended to promote the health of the group represented by the potential subject, the research cannot instead be performed with persons capable of providing informed consent, and the research entails only minimal risk and minimal burden.

29. When a potential research subject who is deemed incapable of giving informed consent is able to give assent to decisions about participation in research, the physician must seek that assent in addition to the consent of the legally authorised representative. The potential subject's dissent should be respected.

30. Research involving subjects who are physically or mentally incapable of giving consent, for example, unconscious patients, may be done only if the physical or mental condition that prevents giving informed consent is a necessary characteristic of the research group. In such circumstances the physician must seek informed consent from the legally authorised representative. If no such representative is available and if the research cannot be delayed, the study may proceed without informed consent provided that the specific reasons for involving subjects with a condition that renders them unable to give informed consent have been stated in the research protocol and the study has been approved by a research ethics committee. Consent to remain in the research must be obtained as soon as possible from the subject or a legally authorised representative.

31. The physician must fully inform the patient which aspects of their care are related to the research. The refusal of a patient to participate in a study or the patient's decision to withdraw from the study must never adversely affect the patient-physician relationship.

32. For medical research using identifiable human material or data, such as research on material or data contained in biobanks or similar repositories, physicians must seek informed consent for its collection, storage and/or reuse. There may be exceptional situations where consent would be impossible or impracticable to obtain

for such research. In such situations the research may be done only after consideration and approval of a research ethics committee.

Use of Placebo

33. The benefits, risks, burdens and effectiveness of a new intervention must be tested against those of the best proven intervention(s), except in the following circumstances:

Where no proven intervention exists, the use of placebo, or no intervention, is acceptable; or

Where for compelling and scientifically sound methodological reasons the use of any intervention less effective than the best proven one, the use of placebo, or no intervention is necessary to determine the efficacy or safety of an intervention

and the patients who receive any intervention less effective than the best proven one, placebo, or no intervention will not be subject to additional risks of serious or irreversible harm as a result of not receiving the best proven intervention.

Extreme care must be taken to avoid abuse of this option.

Post-Trial Provisions

34. In advance of a clinical trial, sponsors, researchers and host country governments should make provisions for post-trial access for all participants who still need an intervention identified as beneficial in the trial. This information must also be disclosed to participants during the informed consent process.

Research Registration and Publication and Dissemination of Results

35. Every research study involving human subjects must be registered in a publicly accessible database before recruitment of the first subject.

36. Researchers, authors, sponsors, editors and publishers all have ethical obligations with regard to the publication and dissemination of the results of research. Researchers have a duty to make publicly available the results of their research on human subjects and are accountable for the completeness and accuracy of their reports. All parties should adhere to accepted guidelines for ethical reporting. Negative and inconclusive as well as positive results must be published or otherwise made

publicly available. Sources of funding, institutional affiliations and conflicts of interest must be declared in the publication. Reports of research not in accordance with the principles of this Declaration should not be accepted for publication.

Unproven Interventions in Clinical Practice

37. In the treatment of an individual patient, where proven interventions do not exist or other known interventions have been ineffective, the physician, after seeking expert advice, with informed consent from the patient or a legally authorised representative, may use an unproven intervention if in the physician's judgement it offers hope of saving life, re-establishing health or alleviating suffering. This intervention should subsequently be made the object of research, designed to evaluate its safety and efficacy. In all cases, new information must be recorded and, where appropriate, made publicly available.

© World Medical Association, Inc. - All Rights reserved.
© Asociación médica mundial - Todos los derechos reservados.
© L'Association Médicale Mondiale - Tous droits réservés.

Appendix V

Sponsor's Signature Page

PROTOCOL APPROVAL PAGE

Protocol No.: COVOVAX-Booster

Version No.: 4.0

Date: 21 Apr 2022

Amendment No.: 3

Study Title: A phase 3, observer-blind, randomized, controlled study to evaluate the safety and immunogenicity of a booster dose of COVOVAX in Indian adults who have received primary vaccination against COVID-19

[redacted]

Appendix VI

Investigator’s statement of compliance

STATEMENT OF COMPLIANCE

Study Title: A Phase 3, Observer-Blind, Randomized, Controlled Study to Evaluate the Safety and Immunogenicity of a Booster Dose of Covovax in Indian Adults Who Have Received Primary Vaccination Against Covid-19

Protocol No.: COVOVAX-Booster

Version: 4.0 Dated 21 Apr 2022

This study will be conducted in compliance with the approved clinical trial protocol, institution ethics committee and informed consent regulations and ICH GCP guidelines. The study will be conducted according to current revision of the Declaration of Helsinki (Revised Fortaleza, 2013). In addition, most current version of local regulatory and ethical requirements 'Guidelines for Clinical Trials on Pharmaceutical Products in India – GCP Guidelines' issued by Central Drugs Standard Control Organization (CDSCO), Ministry of Health, Government of India in 2005, 'Requirements and guidelines for permission to import and / or manufacture of new drugs for sale or to undertake clinical trials' (New Drugs and Clinical Trials Rules, 2019) and it's amended rules and 'Ethical Guidelines for Biomedical Research on Human Subjects' issued by Indian Council of Medical Research will be adhered to.

Principal Investigator	Signature	Date

Appendix VII

Study Sites and Principal Investigator Information

GENERAL INFORMATION: PRINCIPAL INVESTIGATORS AND STUDY SITES

**Title**	A phase 3, observer-blind, randomized, controlled study to evaluate the safety and immunogenicity of a booster dose of COVOVAX in Indian adults who have received primary vaccination against COVID-19
**Protocol No.**	COVOVAX-Booster
**Version and Date**	Version: 4.0 Dated 21 Apr 2022
**Phase**	3
**Sponsor**	**SERUM INSTITUTE OF INDIA PVT. LTD.**<br>212/2, Off Soli Poonawalla Road,<br>Hadapsar, Pune, Maharashtra-411028, India<br>Phone: +91 20 26602384
[redacted]	[redacted]

	[redacted]
	[redacted]
	[redacted]
	[redacted]
